# New Route to Glycosylated Porphyrins via Aromatic Nucleophilic Substitution (S_N_Ar)—Synthesis and Cellular Uptake Studies

**DOI:** 10.3390/ijms231911321

**Published:** 2022-09-26

**Authors:** Mariusz Rosa, Natalia Jędryka, Sandra Skorupska, Ilona Grabowska-Jadach, Maciej Malinowski

**Affiliations:** Faculty of Chemistry, Warsaw University of Technology, Ul. Noakowskiego 3, 00-664 Warsaw, Poland

**Keywords:** glycoporphyrins, photosensitisers, SNAr, nucleophilic aromatic substitution, cellular uptake, photodynamic therapy, carbohydrates, porphyrins

## Abstract

Glycoporphyrins are group of compounds of high value for the purpose of photodynamic therapy and other biomedical applications. Despite great progress in the field, new diversity-oriented syntheses of carbohydrate-porphyrin hybrids are increasingly desired. Herein, we present efficient, mild, and metal-free conditions for synthesis of glycoporphyrins. The versatile nature of the S_N_Ar procedure is presented in 16 examples. Preliminary biological studies have been conducted on the cytotoxicity and cellular uptake of the final molecules.

## 1. Introduction

For over 150 years, nucleophilic aromatic substitution (S_N_Ar) has been recognised as an essential tool for nucleophilic functionalisation of aromatic compounds [1,2,3]. Nowadays, a similar type of reactivity is achieved and developed via organometallic transformations [4,5,6,7]. However, for electron deficient compounds, S_N_Ar still remains a promising synthetic choice, since this type of reactivity does not require the presence of any metal catalyst [8]. It might be especially beneficial for the synthesis of products for medical uses, because traces of metals might change the biological response to the final molecules.

The S_N_Ar reactivity type has been already recognised as a reliable strategy for functionalisation of sugars or porphyrinoids. It is known method for *O*-arylation of anomeric position in carbohydrates [9,10,11], However, the examples of aromatic nucleophilic substitutions of different positions in sugars are particularly limited [12,13,14,15]. It is especially intriguing since this type of reactivity might lead to unnatural *O*-arylated products more resistant to hydrolysis than their C-1 *O*-arylated equivalents. Similarly to carbohydrate chemistry, the S_N_Ar functionalisation of porphyrins has been widely exploited at particular positions, namely, the *meso*- or *β*-position [16]. Peripheral *meso*-phenyl ring functionalisation has been reported [17,18,19,20,21,22,23,24,25], mostly on perfluorinated derivatives, while for nitroporphyrins S_N_Ar has been suggested as a side-reactivity only [26,27]. Hence, it is a promising challenge for both group of compounds—sugars and porphyrins—to functionalise and combine them via the unexplored side. As a result, access to new derivatives with an interesting set of features would be possible.

The biomedical application of porphyrinoids is being regularly investigated and developed because they are considered great candidates for photosensitisers (PS) in photodynamic therapy (PDT) [28,29,30]. However, despite their promising photophysical potential, most of these compounds suffer from low solubility in the physiological milieu, which limits their possible application. What is more, the new generation of photosensitisers are desired to have an increased cellular uptake in cancer cells [31,32,33]. In both aspects, the expected improvement of PS properties might be achieved by introduction of sugar moiety onto the porphyrin system [31,34,35,36]. The involvement of the Warburg effect might provide better selectivity towards cancer cells; furthermore, the presence of polar moieties should increase amphiphilicity, hence providing better solubility in polar solvents.

Glycoporphyrins have already proved their meaningful potential and possible applications in modern science, such as catalytic activity in asymmetric reactions [37,38,39], as well as their biomedical perspectives, including antiviral properties [28,40] or photodynamic inactivation effects [41,42,43]. Despite significant progress in the field [44,45,46], the chemical synthesis of new sugar–porphyrin hybrids still remains a challenging task and the literature does not cover many general methodologies towards libraries of this type of compounds. This is probably due to the differences in chemical nature of both the counterparts to be combined. Sugars are polyhydroxylated hydrophilic aliphatic compounds, while, on the other hand, porphyrin chemistry deals with lipophilic aromatic molecules. To remain efficient, new methodologies should be a fair compromise the between chemical properties of both moieties. Recently, we have published a new route to glycoporphyrins via the Sonogashira reaction [47]. In our current research, we decided to develop a new procedure of synthesis of sugar–porphyrin hybrids without the presence of any metal catalyst. Taking all of this into account, we decided to study nucleophilic aromatic substitution as a general tool to introduce carbohydrate units onto the porphyrin system.

**Scheme 1:**
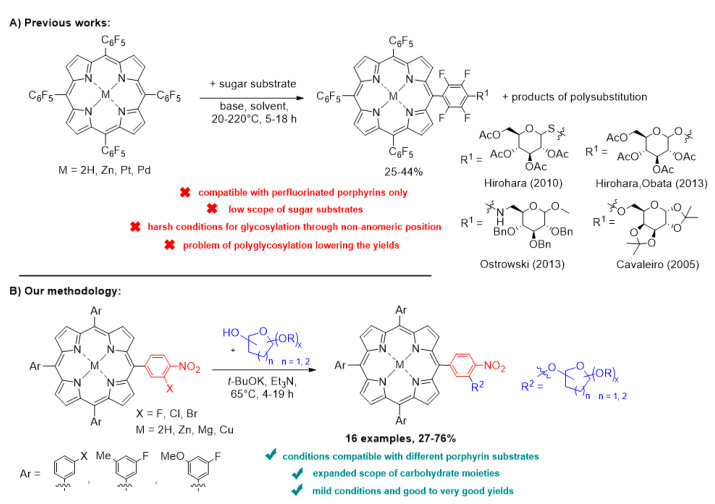
Our strategy for synthesis of glycoporphyrins in comparison with known strategies of S_N_Ar reactions performed on porphyrins.

The potential of the S_N_Ar reaction in the synthesis of glycoporphyrins has been presented only on the perfluorinated porphyrins (Scheme 1A) [40,48,49,50]. However, in the described strategies, the sugar substrate scopes are very limited, and some procedures required harsh conditions. What is more, the perfluorinated porphyrins easily underwent polysubstitution, which lowered the yield and created a challenge of isolating the monoglycosylated hybrid. Last but not least, despite the great chemical attractiveness of this model starting material, the use of perfluorinated compounds raises the important issues of their potential risk for human health and the problem of efficient environmental removal and remediation [51,52,53].

Considering the aforementioned state of knowledge, we decided to develop a methodology that would be more diverse and would allow the combination of carbohydrates with non-perfluorinated porphyrins. Herein, we present mild and efficient conditions for glycosylation of porphyrins via S_N_Ar reactivity—a convenient, diversity-oriented strategy (Scheme 1B). The resulting glycoporphyrin hybrids are an interesting example of amphiphilic compounds with a limited amount of halogen atoms and with increased cellular uptake for the purpose of PDT.

## 2. Results and Discussion

### 2.1. Initial Studies and Optimisation of the S_N_Ar Process

**Scheme 2:**
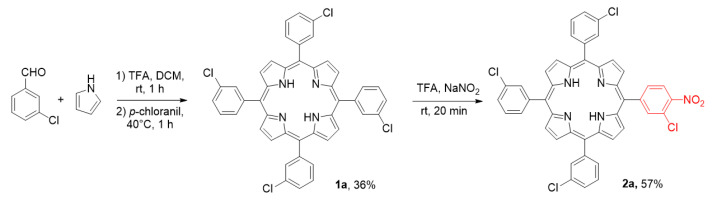
Synthesis of model porphyrin; TFA—trifluoroacetic acid.

Since aromatic nucleophilic substitution has been already recognised for perfluorinated porphyrins, we began our research from selection of model compound that would allow us to limit the number of halogen atoms in the final products. As a convenient substrate, we decided to explore the nitroporphyrin **2a** because its synthesis is relatively simple (Scheme 2) and has been investigated in the past research of our group [54,55]. The porphyrin core has been synthesised with Lindsey’s procedure [56]. The nitration procedure has been elaborated in recent studies of our group [57]. By applying this strategy, we could easily obtain substrate **2a**, a convenient starting material for S_N_Ar studies, since the halogen atom in this compound should be activated enough by the strong electron withdrawing effect of the nitro group at the ortho-position.

On the opposite side, as a model carbohydrate moiety for studies, we decided to use commercially available 1,2;3,4-di-*O*-isopropylidene-α-d-galactopyranose (**3a**). The choice of sugar derivative was influenced by two factors. First of all, sugar derivative **3a** possess the primary hydroxyl group, and the S_N_Ar should therefore proceed in milder conditions. Furthermore, the beneficial effect of galactose moiety recognition by cancer cells has already been proven in the literature [35,36]. It mostly exploits the fact that galectin-1 is overexpressed in tumours and has a high affinity to *β*-galactose-containing oligosaccharides. Hence, the molecules bearing a galactose moiety should be more preferentially targeted by cancer cells over normal tissues. Thus, the new methodology giving access to this type of galactoporphyrin was reasonable in terms of chemical and biomedical use.

**Scheme 3:**
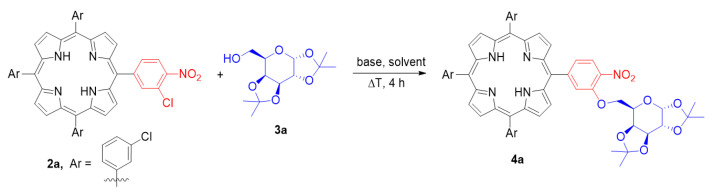
Optimisation of S_N_Ar reaction on model compounds.

With the model compounds, we started studies on the process of S_N_Ar with the carbohydrate derivative as a substrate (Scheme 3; for detailed studies, see Supplementary Materials). As an initial experiment, we decided to test Williamson-type conditions, but no conversion was observed (Table 1, entry 1). The change of base on sodium hydroxide, used in great excess, led to obtaining product **4a** at low yield (Table 1, entry 2). We observed low stability of the solvent in these conditions and active formation of side products from hydroxide attack on the activated C-3 position. Therefore, we decided to find an alternative base for the reaction. The application of *t*-BuOK was a promising choice; it resulted in product formation and no side product was observed (Table 1, entry 3). However, the porphyrin **2a** conversion remained low. We found that a high excess of base was essential when DMF was used as a solvent. Decreasing the amount of *t*-BuOK resulted in a significant reduction of the yield; only traces of product were detected (Table 1, entry 4). To improve the yield, we decided to screen reaction solvents (Table 1, entries 5–7). Interestingly, the most promising solvent was triethylamine, leading to product **4a** with a very good 70% yield (Table 1, entry 7). This might be due to an important role of solvent in determining the basicity of *t*-BuOK and/or by modification of reaction kinetics by providing good solubility of both starting materials. What is more, on the contrary to other solvents tested, only in Et_3_N did we not observe formation of other unidentified side products. The beneficial effect of the sub-stoichiometric amount of triethylamine on S_N_Ar reactivity has already been reported for more acidic thiols [58]. In order to provide the most optimal use of all reagents, we decided to further test our reaction system by making it compatible with milder conditions. We discovered that reaction proceeds efficiently at lower temperatures as well (65 °C and 50 °C), furnishing the product with good yields of 72% and 65%, respectively (Table 1, entries 8,9). Moreover, increasing the concentration of reagents allowed us to reduce the excess of the base and sugar substrate (Table 1, entries 10, 11), leading to an optimal yield of 76%. Further decreasing the amount of base resulted in a significant drop in the yield (Table 1, entry 12).

### 2.2. Investigation on the Reaction Scope

**Scheme 4:**
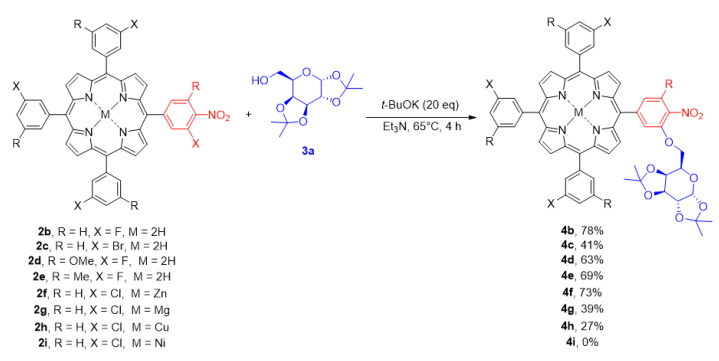
The S_N_Ar glycosylation of diverse porphyrins under developed conditions.

The optimal conditions were then applied to test the reaction scope. We were curious about the versatility of our methodology with regard to the structure of the peripheral aromatic ring. In our recent projects, we synthesised ortho-halo nitroporphyrins that were adequate starting materials to challenge the developed S_N_Ar procedure [57]. With nitro compounds in hand, we tested several different porphyrins (Scheme 4), proving the versatile character of these conditions. The halogen reactivity followed the classical order. The most reactive was porphyrin **2b** bearing a fluorine atom as a leaving group, leading to glycoporphyrin **4b** with a very good 78% yield. Interestingly, bromide derivative was an active substrate as well; however, the reaction was less efficient and product **4c** was obtained at a 41% yield. The substituents at the C-5 position of phenyl rings did not affect the glycosylation process, yielding products **4d** and **4e** at very good yields of 63% and 69%, respectively. Then, we investigated metalloporphyrin derivatives in our procedure. We observed that the most suitable porphyrin chelate was zinc(II) complex, leading to product **4f** at a 73% yield. Magnesium porphyrin derivative was less stable, which resulted in a drop of the yield to 39% of product **4g**. Surprisingly, lower conversion was observed for porphyrin complexed with more electronegative metals. Copper(II) porphyrin derivative led to the product **4h** at a 27% yield, and no improvement was detected for prolongated reaction times. Moreover, glycosylation via S_N_Ar did not occur under developed conditions for porphyrin complexed with nickel(II). The exact nature of this phenomenon is under careful investigation since, to the best of our knowledge, a similar tendency in the reactivity of porphyrin complexes has not yet been reported in the literature.

**Scheme 5:**
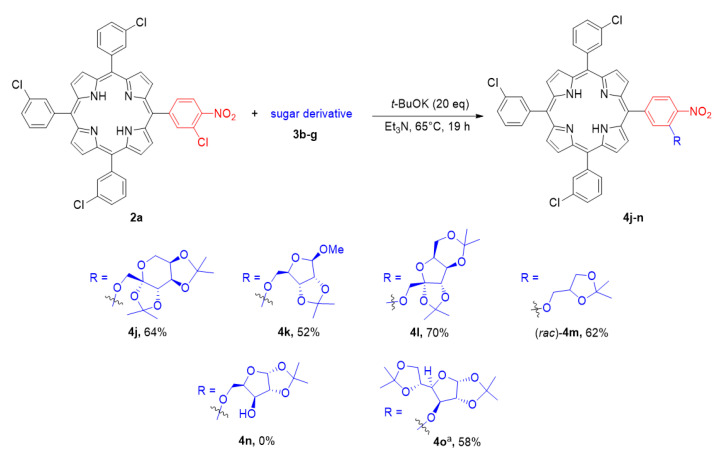
The scope of the S_N_Ar process with regards to the sugar reagent. ^a^ The reaction was conducted at 75 °C for 48 h.

The next step of our research was devoted to studies on the limitations of our methodology with regard to the selection of carbohydrate moiety (Scheme 5). We have observed that the reaction proceeds with the best yields when only the primary hydroxyl group of the sugar reagent is available for the reaction. Contrary to the previous results, the prolongation of the reaction time to 19 h resulted in the yield improvement of about 5–30%, so this modification was applied for most of the examples in this series. By applying the aforementioned procedure, we glycosylated porphyrin **2a** with the d-fructose derivative **3b**, leading to product **4j** at a good yield of 64%. The conditions were also suitable to introduce d-ribose (**4k**, 52%), l-sorbose (**4l**, 70%), and solketal ((*rac*)-**4m**, 62%) moieties. The limitation for our glycosylation method is the presence of only one hydroxyl function at the sugar molecule. We observed a lack of porphyrin conversion if the carbohydrate substrate was a d-xylose derivative bearing two hydroxyl groups. In this case, no product (**4n**) was formed in the S_N_Ar process. The transformation of the secondary hydroxyl group was possible, giving a satisfying yield of glucose–porphyrin hybrid (**4o**, 58%). However, due to the steric hindrance and therefore lower reactivity, the reaction temperature should be raised to 75 °C and the reaction time should be prolonged to 48 h.

**Scheme 6:**
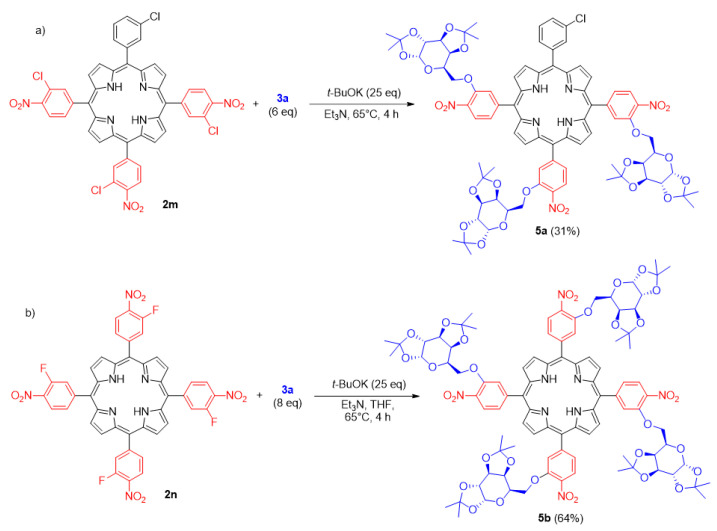
(**a**,**b**) The examples of porphyrin polysubstitution.

We also challenged the developed S_N_Ar conditions with the polyglycosylation process. We discovered that the procedure is adequate to introduce more than one carbohydrate unit onto the porphyrin peripheral positions. In our studies, we obtained tri- and tetra-substituted glycoporphyrins (**5a** and **5b**, Scheme 6). The lower reactivity of porphyrins with chlorine atoms was insignificant for the monoglycosylation (scope studies, Scheme 3 and Scheme 4), whereas it multiplied and became an important factor in the case of polysubstitution. The product **5a** was obtained at an average yield of 31%, while the more reactive nitro compound **2n** with fluorine atoms led to the hybrid **5b** at a very good overall yield of 64%. Nevertheless, both experiments proved that with only a little adaptation of the developed procedure (some amount of THF to improve solubility of **2n**), the process of polyglycosylation might be achieved via the same methodology.

**Figure 1:**
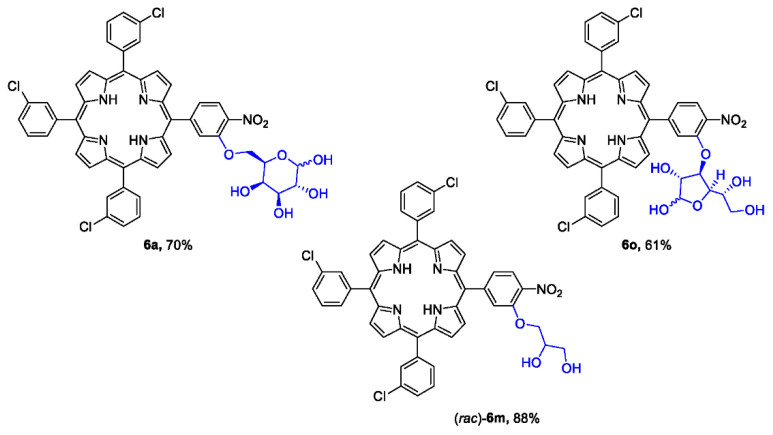
The structures of final products and the yields of the deprotection steps. The reaction was conducted at 50 °C for 3 h in 35% HCl_(aq)_.

Biological studies were essential for the deprotected forms of glycoporphyrins to confirm their utility in PDT. Hence, the protecting group removal procedure was an important procedure to develop. We tested several different methods of hydroxyl group deprotection. We observed that typical conditions (a diluted aqueous solution of hydrochloric acid) [59] were insufficient for this type of compound. Moreover, the use of nucleophilic solvents other than water was undesired, since the anomeric position of the sugar moiety was reactive under these conditions. The most efficient deprotection was observed when glycoporphyrins were treated with concentrated hydrochloric acid at an elevated temperature. We applied this procedure for our model compounds **4a**, (*rac*)-**4m**, and **4o,** and the final hydroxylated products (**6a**, (*rac*)-**6m**, **6o**) were obtained at good and very good yields (Figure 1). Due to the chemical nature of the final molecules, excepting compound (*rac*)-**6m**, products were obtained as a mixture of anomers and/or as an equilibrium between their furanose/pyranose forms.

The developed procedure for the S_N_Ar reaction was suitable for the synthesis of a range of glycoporphyrins. We then investigated their deprotected forms in biological studies of cytotoxicity and cellular uptake.

### 2.3. Biological Studies

As the next step of our research, we decided to conduct some preliminary biological experiments in order to determine the possible application of the obtained compounds in photodynamic therapy. For these studies, we selected three deprotected hybrids (**6a**, **6o**, (*rac*)-**6m**), with porphyrin **2b** as a reference compound without a sugar moiety. Compound **2b** was selected from the set of parent porphyrins because among all the unmodified porphyrins, it was the one with the highest solubility in the minimal amount of DMSO required to prepare the solution for biological assays.

#### 2.3.1. Cytotoxicity Assessment

**Figure 2:**
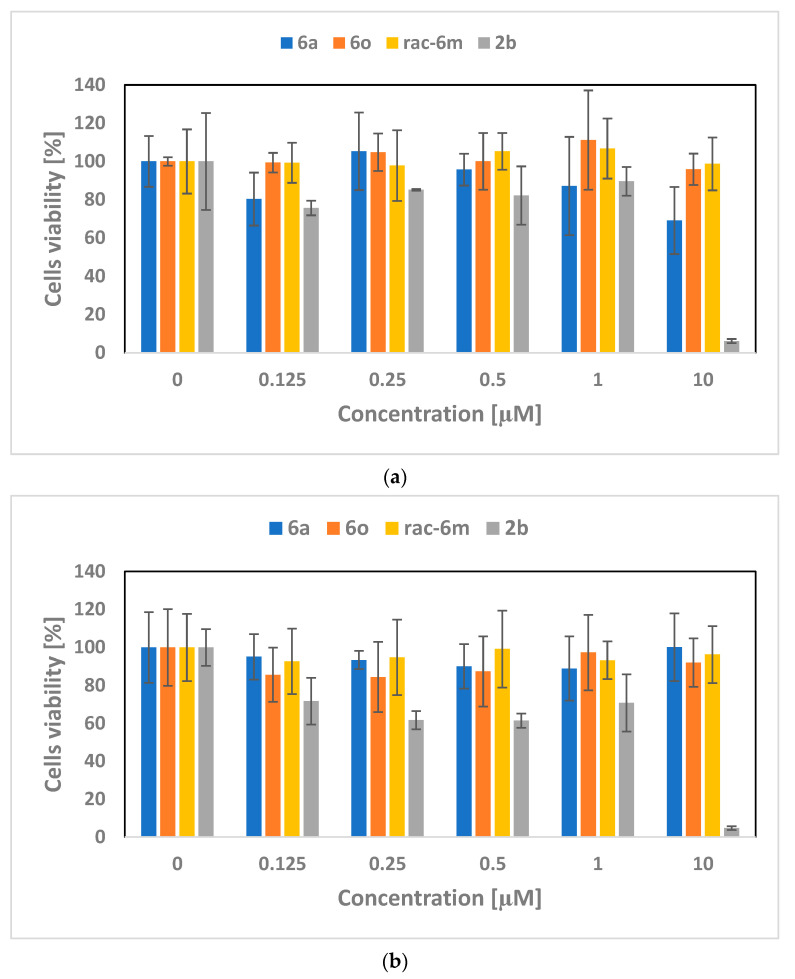
Porphyrin cytotoxicity tests on the cell line: (**a**) A375, (**b**) HaCaT.

The cytotoxicity of selected porphyrins (**6a**, **6o**, (*rac*)-**6m**, **2b**) was investigated on skin cell lines: tumour A375 and normal HaCaT (Figure 2a,b). In the case of the HaCaT cells, no significant reduction in cell viability was noticed after incubation with porphyrins with attached moieties—galactose (**6a**), glucose (**6o**), and glycerine ((*rac*)-**6m**); viability was above 80% for the entire range of concentrations tested. Incubation of normal cell cultures with unmodified porphyrin (**2b**) resulted in greater cell death. HaCaT viability decreased to approx. 60% after incubation with porphyrin solutions in the concentration range of 0.125–1 µM, and as little as below 10% for 10 µM. Similar results were obtained with the cancer cell line. Porphyrins **6o** and (*rac*)-**6m** were not cytotoxic. Incubation of A375 cells with **6a** porphyrin caused a decrease in viability to about 80% for the highest concentration. Again, unmodified porphyrin caused tumour cell mortality at the level of approx. 90% for the highest concentration tested.

On the basis of the obtained results, it can be noticed that porphyrins with attached units—glucose, galactose, and glycerine—do not show a cytotoxic effect in A375 and HaCaT cells in the studied concentration range, unlike **2b** (unmodified) porphyrin, which causes a high rate of cell death after incubation with 10 µM solution.

#### 2.3.2. Cellular Uptake

**Figure 3:**
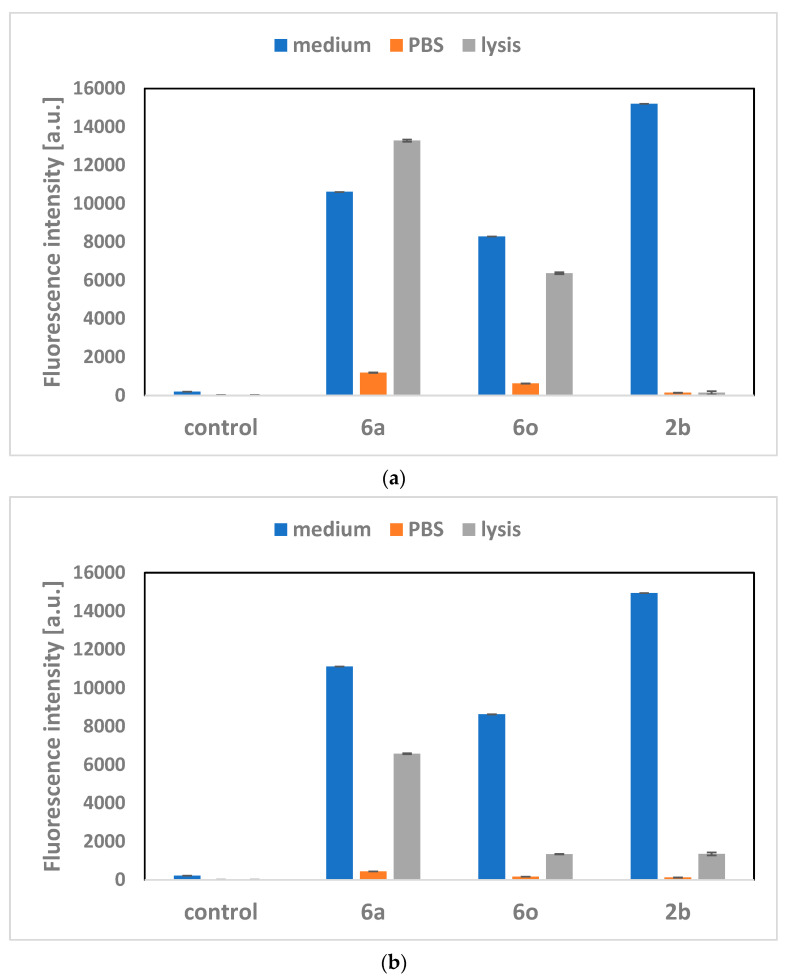
Penetration of porphyrins into cells: (**a**) A549 and (**b**) MCF-7, after incubation of the cells culture with a porphyrin solution. The measured fluorescence intensity of the ‘medium’ samples corresponds to the fluorescence intensity of the porphyrins administered to the cells in the culture medium at the highest concentration (10 µM). ‘PBS’ measurements were made after removing the medium from the cell culture and washing cells with PBS (phosphate-buffered saline). ‘Lysis’ assays refer to the fluorescence intensity of the cells’ lysate after incubation with porphyrins. Based on these measurements, we can find what percentage of the porphyrins administered to the cells (‘medium’ samples) penetrated into them (‘lysis’ samples).

Due to the possibility of using porphyrins as photosensitisers in PDT therapy, their penetration into the cells is an important feature. Porphyrin **2b** is characterised by the fact that it is practically impossible for it to penetrate the cell membrane. The aim of the research on the biological activity of porphyrins was also to check how their accumulation capacity inside cells changes after porphyrin modification. The studies were performed on selected porphyrins (**6a** and **6o**) that were incubated with two different cell lines (Figure 3a,b). Due to the potential use of porphyrins in anticancer therapy, the studies used two cancer cell lines originating from the lungs and breasts. The conducted research confirms that porphyrins **6a** and **6o** (galactose and glucose moieties) penetrate into A549 and MCF-7 cells and accumulate in them. For both **6a** and **6o**, accumulation occurs to a greater extent in lung cells (A549) than in breast cells (MCF-7). It was observed that more porphyrin **6a** enters the cells compared to porphyrin **6o**. Based on these results, it can be concluded that both porphyrins can potentially be used as photosensitisers in PDT therapy.

## 3. Methods and Materials

The detailed description of all synthetic procedures and spectroscopic data of all new compounds are included in Supplementary Materials.

### 3.1. General Procedure for Synthesis of Porphyrin-Sugar Hybrids

In a sealed tube (6 mL), porphyrin (0.013 mmol, 1.0 eq), sugar substrate (0.026 mmol, 2.0 eq), 0.35 mL of triethylamine, and then 29.5 mg of potassium *t*-butoxide (0.260 mmol, 20.0 eq) were added. The reaction mixture was stirred for 4 h at 65 °C. Then, it was cooled to room temperature and transferred to a separatory funnel with 20 mL of chloroform. The organic layer was washed with water (3 × 20 mL) and dried with anhydrous MgSO_4_. The drying agent was filtered off; then, solvent was evaporated on a rotary evaporator. The crude product was purified by column chromatography using the indicated eluent.

### 3.2. Cell Cultures

Experiments were carried out on four human cell lines: keratinocyte—HaCaT (purchased from ThermoFisher (Waltham, MA, USA)), malignant melanoma—A375 (purchased from ATCC (Manassas, VA, USA)), breast adenocarcinoma—MCF-7 (purchased from ATCC (Manassas, VA, USA)), and lung carcinoma—A549 (purchased from ATCC (Manassas, VA, USA)). The culture medium for HaCaT, A375, and MCF-7 cells was based on Dulbecco’s modified Eagle’s medium (DMEM, Biowest (Nuaille, France)), supplemented with 10% of fetal bovine serum (FBS, Biowest (Nuaille, France)), 1% 25 mM l-glutamine (Biowest (Nuaille, France)), 1% 100 mM penicillin, and streptomycin (Biowest (Nuaille, France)). The culture medium for A549 differed only in the base; it consisted of minimum essential medium (MEM, Biowest (Nuaille, France)) with the same additives as before. The cells were sub-cultured every two days using Tryple Express (Gibco (Waltham, MA, USA)) solution to detachment. Cells were kept at 37 °C and 5% CO_2_ in a humidified atmosphere.

### 3.3. Cytotoxicity Test

The cytotoxicity of the tested compounds was determined by MTT reduction test. The cells were seeded into a 96-well plate and the day after were incubated with selected compounds at 37 °C and 5% CO_2_ in a humidified atmosphere for 24 h. Subsequently, a supernatant was removed and tetrazolium dye (3-(4,5-dimethylthiazol-2-yl)-2,5-diphenyltetrazolium bromide (Sigma-Aldrich (St. Louis, MO, USA)) solution (0.5 mg/mL in phosphate-buffered saline) was added—100 μL per well. After 4 h of incubation at 37 °C and 5% CO_2_ in a humidified atmosphere, the supernatant was carefully removed. Then, dimethyl sulfoxide solution (DMSO, Sigma-Aldrich (St. Louis, MO, USA)) was added (100 μL per well) to dissolve the formed formazan crystals. Next, absorbance at 570 nm was measured using a multi-mode reader (Cytation 3, BioTek (Santa Clara, CA, USA)). The results were presented as percentage of viable cells compared to the control sample.

### 3.4. Ability of Compounds to Penetrate Cells

The penetration of the test compounds was assessed according to the following procedure. After seeding the cells in a 96-well plate for 24 h, they were incubated overnight with selected compounds. Subsequently, the fluorescence intensity was measured (excitation wavelength 430 nm, emission wavelength 660 nm). In the next step, the medium was removed, the cells were washed twice with a phosphate buffer solution, and the fluorescence intensity was measured again. The cells were then lysed with a 3% Triton X 100 solution (Sigma-Aldrich (St. Louis, MO, USA)) for 50 min and the fluorescence intensity was measured.

## 4. Conclusions

A new methodology for glycoporphyrin synthesis using the S_N_Ar reaction has been presented. Following the procedure, 16 new examples of glycoporphyrins were obtained, proving the versatile character of this method and providing an interesting alternative to the known procedures of glycoporphyrin synthesis. The presented method does not require use of any metal catalysts; however, a high excess of base is essential to the efficient transformation. The biological studies proved that the synthesised hybrids might be promising agents for PDT. They featured low cytotoxicity and, moreover, the porphyrins bearing a sugar substituent (**6a**, **6o**) accumulated in cells significantly more (A549, MCF-7) than unglycosylated porphyrin **2b**. Future research will focus on further determination of the usefulness of these compounds in photodynamic therapy and the development of new procedures of glycoporphyrin synthesis.

## Figures and Tables

**Table 1:** Optimisation of S_N_Ar reaction between model porphyrin **2a** and carbohydrate **3a** ^1^.

Entry	Solvent	Base (Equiv.)	Temperature [°C]	Yield [%]
1.	DMF	NaH (100)	100	0
2.	DMF	NaOH (200)	80	17
3.	DMF	*t*-BuOK (200)	80	15
4.	DMF	*t*-BuOK (60)	80	traces
5.	*t*-BuOH	*t*-BuOK (60)	80	0
6.	THF	*t*-BuOK (60)	80	traces
7.	Et_3_N	*t*-BuOK (60)	80	70
8.	Et_3_N	*t*-BuOK (60)	65	72
9.	Et_3_N	*t*-BuOK (60)	50	65
10.	Et_3_N^2^	*t*-BuOK (20)	65	74
**11.**	**Et_3_N ** ^2,3^	***t-* BuOK (20)**	**65**	**76**
12.	Et_3_N ^2,3^	*t*-BuOK (10)	65	45

^1^ Typical conditions: porphyrin **2** (0.013 mmol), carbohydrate **3a** (6 eq), solvent (1 mL), ΔT, 4 h. ^2^ Reaction in 0.35 mL of Et_3_N. ^3^ Reaction with 2 eq of sugar **3a**.

## Data Availability

A copy of the NMR spectra of the new compounds is available in the Supplementary Materials.

## Supplementary Materials

The supporting information can be downloaded at: https://www.mdpi.com/article/10.3390/ijms231911321/s1. References [26,60,61,62,63] are cited in the Supplementary Materials.
