# Supplementary material for: New Route to Glycosylated Porphyrins via Aromatic Nucleophilic Substitution (SNAr)—Synthesis and Cellular Uptake Studies
Source: Int J Mol Sci. 2022 Sep 26;23(19):11321. doi: 10.3390/ijms231911321 (PMC9570116; doi:10.3390/ijms231911321)

Supplementary Materials

New Route to Glycosylated Porphyrins via the Aromatic Nucleophilic Substitution (S_NAr) – Synthesis and Cellular Uptake Studies

Mariusz Rosa, Natalia Jędryka, Sandra Skorupska, Ilona Grabowska-Jadach and Maciej Malinowski*

Faculty of Chemistry, Warsaw University of Technology, ul. Noakowskiego 3, 00-664 Warsaw, Poland.

corresponding author e-mail: maciej.malinowski@pw.edu.pl

Table of content

General experimental methods 2

Optimisation studies of S_NAr reaction of porphyrin 2a 3

Procedures and characterisation of starting materials 4

Characterization and procedures for the synthesis of porphyrin-sugar hybrids 11

Characterization and procedures for deprotected sugar hybrids 25

NMR and UV-VIS spectra of new compounds 28

General experimental methods

Nitration, synthesis of porphyrin-sugar hybrids and deprotection of sugar moieties were carried out using sealed tubes. Chloroform was dried over $CaCl_2$ and distilled. Triethylamine was stored over KOH. Other solvents were used without further purification. The nitrocompounds **2b**-**2e** have been synthesized following similar procedure as described for **2a**.[1] The sugar starting materials: **3a**, **3d**, **3e** and **3g** have been purchased from Carbosynth or Sigma Aldrich. Compounds **3b**, **3c** and **3f** were synthesized as described herein. Commercially available chemicals were used as received unless otherwise stated. Reactions were monitored by thin-layer chromatography on aluminium foil plates pre-coated with silica gel (DC Alurolle, Kieselgel 60, F-254; Merck AG) which were observed under visible light and rendered by ultraviolet. Column chromatography was performed with Silica gel (230-400 mesh). ^{1}H NMR (500 MHz), ^{13}C NMR (125 MHz), ^{19}F NMR (471 MHz) were recorded at 293 K with a Varian NMR System. Chemical shifts are given in ppm (δ) and are referenced to the internal solvent signal or to TMS used as an internal standard. Multiplicities are declared as follows: s (singlet), br s (broad singlet), d (doublet), t (triplet), dd (doublet of doublets), ddd (doublet of doublet of doublets), m (multiplet). Coupling constants *J* are given in Hz. Mass spectra were measured with a Synapt G2-S HDMS (Waters, ESI-TOF) spectrometer (ESI method and APCI method); *m/z* intensity values for peaks are given as % of relative intensity. UV/Vis spectra were measured with the LLG-*uniSPEC* 2 Spectrophotometer.

[1] M. Rosa, M. Malinowski, S. Ostrowski, Modification for Nitration of Halo-Substituted Meso-Tetraarylporphyrins: A Convenient Scale-up in Small Amount of Solvent. *Curr. Org. Synth.*, Manuscript submitted for publication.

Optimisation studies of S_NAr reaction of porphyrin 2a[a]

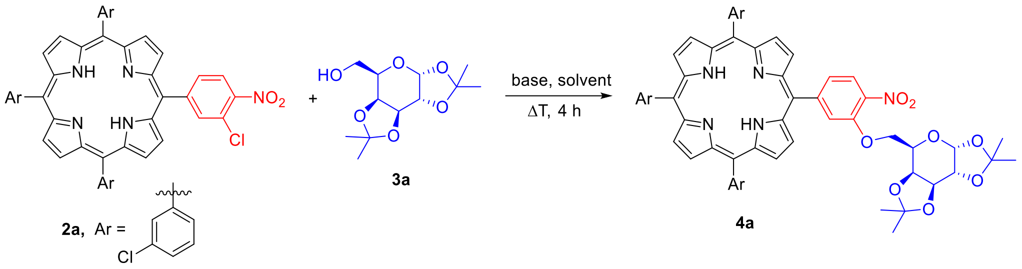

Entry	Solvent	Base (eq)	Temperature [˚C]	Yield [%]
**1**	DMF	NaH (100)	100	0
**2**	NMP	NaOH (200)	80	0
**3**	DMF	NaOH (200)	80	17
**4**	DMF	*t*-BuOK (200)	80	15
**5**	DMF	*t*-BuOK (60)	80	traces
**6**	NMP	*t*-BuOK (60)	80	0
**7**	DMSO	*t*-BuOK (60)	80	0
**8**	*t*-BuOH	*t*-BuOK (60)	80	0
**9**	THF	*t*-BuOK (60)	80	traces
**10**	Et_3N	*t*-BuOK (60)	80	70
**11**	Et_3N	*t*-BuOK (60)	65	72
**12**	Et_3N	*t*-BuOK (60)	50	65
**13**	Et_3N	*t*-BuOK (20)	65	42
**14**	Et_3N[b]	*t*-BuOK (20)	65	74
**15**	**Et_3N[b,c]**	***t*-BuOK (20)**	**65**	**76**
**16**	Et_3N[b,c]	*t*-BuOK (10)	65	45
**17**	Et_3N[b,c]	DBU (20)	65	traces
**18**	Et_3N[b,c]	TMG (20)	65	0
**19**	Et_3N[b,c]	NaH (20)	65	0
**20**	Et_3N[b,c]	Cs_2CO_3 (20)	65	0

[a] Typical conditions: porphyrin **2a** (0.013 mmol), carbohydrate **3a** (6eq), solvent (1 mL), 4h. [b] Reaction in 0.35 ml of Et_3N. [c] Reaction with 2 eq of sugar **3a**.

Procedures and characterisation of starting materials

*5,10,15,20-tetrakis-(3-chlorophenyl)porphyrin (**1a**)*

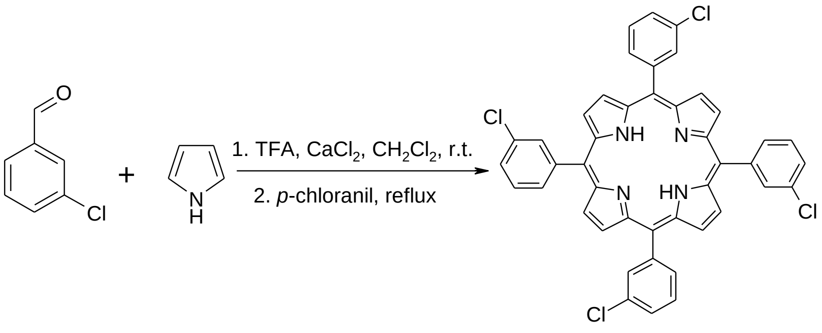

1a

300 mL of CH_2Cl_2 (distilled over $CaCl_2$) was added to a three-necked 500 mL round-bottomed flask equipped with a reflux condenser. Flask was shielded from the ambient light. Then 3-chloro-benzaldealdehyde, 1.53 g (1.8 mL; 16.75 mmol), pyrrole 1.24 g (1.2 mL; 18.43 mmol) and 1.31 g $CaCl_2$ were added. The mixture was stirred for 5 minutes and 2.0 mL TFA (10.45 mmol) was added. Reaction was stirred for 45 min at room temperature. After that time *p*-chloranil 3.00 g (12.20 mmol) was added and the mixture was refluxed for 10 min. Then TFA was neutralized with an excess of Na_2CO_3 and solvent was evaporated off. Product was purified by column chromatography (eluent: chloroform/*n*-hexane, 3:1) yielding purple solid of porphyrin **1a**: 1.13 g, 36%. Spectroscopic data were in agreement with those of the literature.[2]

*5-(3-chloro-4-nitrophenyl)-10,15,20-tris(3-chlorophenyl)porphyrin (**2a**)*[1]

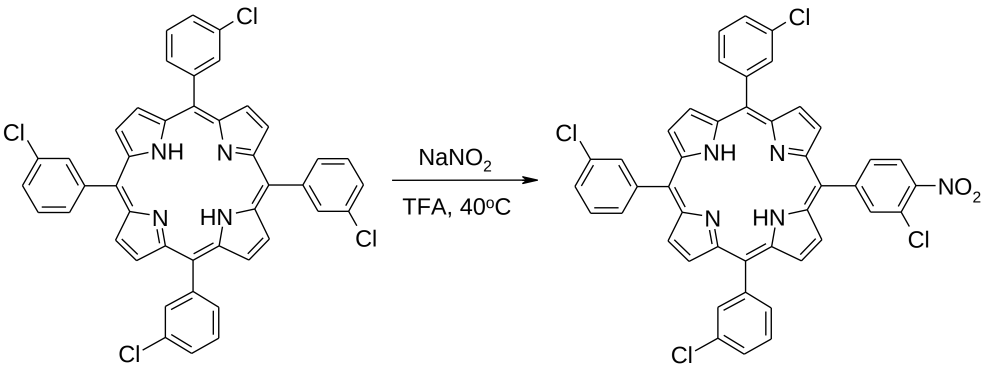

1a **2a**

The porphyrin **1a** (200.8 mg, 0.267 mmol) and 147 mg of sodium nitrite (2.13 mmol) were dissolved in 2.4 mL of TFA in a 10 mL sealed tube. The mixture was vigorously stirred (1000 rpm) for 20 min at 40°C. Then reaction was quenched with 2.0 mL of water, diluted with 80 mL of chloroform, transferred to a separatory funnel and washed with 10% aqueous solution of sodium carbonate (50 mL). The organic layer was then subsequently washed 2x75 mL of water and dried over $MgSO_4$. Then drying agent was filtered off, solvent was evaporated off and a product was separated by column chromatography (eluent: $CHCl_3$/*n*-hexane, 2:1) yielding

[2] S. Ostrowski, B. Łopuszyńska, *Synth. Commun.*, **2003**, *33*, 4101.

purple solid of porphyrin **2a**: 122.0 mg, 57%. Spectroscopic data were in agreement with those of the literature.[2]

*[5-(3-chloro-4-nitrophenyl)-10,15,20-tris(3-chlorophenyl)porphyrinato] zinc(II) (**2f**)*

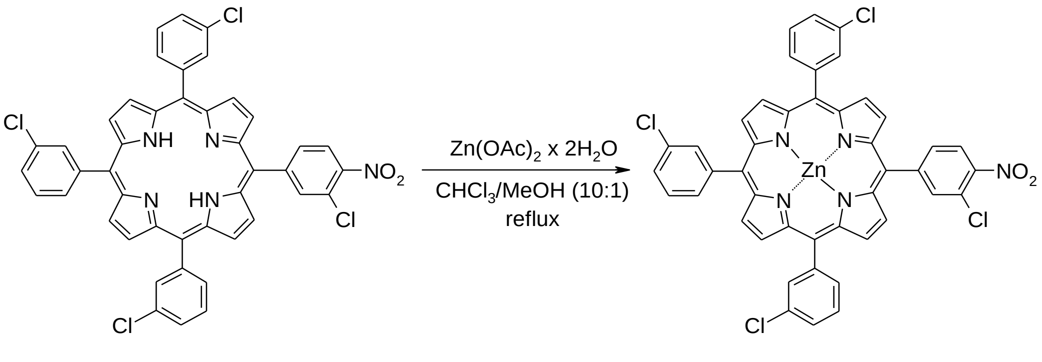

2a **2f**

To a 50 mL round bottomed flask equipped with a reflux condenser 30.0 mg of porphyrin **2a** (0.038 mmol), 150 mg of zinc(II) acetate dihydrate (0.683 mmol), 10 mL of chloroform and 1 mL of methanol were added. The mixture was stirred at reflux for 15 minutes. After cooling to room temperature solvents were evaporated off. Crude product was purified by column chromatography (eluent: chloroform/*n*-hexane, 2:1) yielding purple solid of porphyrin **2f**: 32.0 mg, 99%. Spectroscopic data were in agreement with those of the literature.[3]

*[5-(3-chloro-4-nitrophenyl)-10,15,20-tris(3-chlorophenyl)porphyrinato] magnesium(II) (**2g**)*

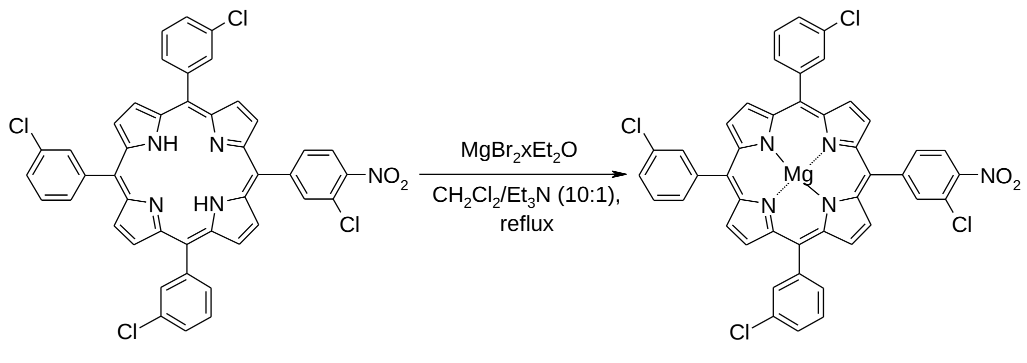

2a **2g**

To a 50 mL round bottomed flask equipped with a reflux condenser 30 mg of porphyrin **2a** (0.038 mmol), 150 mg of magnesium bromide ethyl etherate (0.581 mmol), 10 mL of methylene chloride and 1 mL of triethylamine were added. Mixture was stirred at reflux for 30 minutes. After cooling to r.t. solvents were evaporated off. Crude product was purified by column chromatography (eluent: methylene chloride/methanol/triethylamine, 200:2:1) yielding purple solid of porphyrin **2g**: 26.8 mg, 87%.

^{1}H NMR (500 MHz, DMSO-d$_6$) δ [ppm] = 8.87-8.77 (m, 8H, H$^\beta$); 8.60-8.56 (m, 1H, H-Ar); 8.49 (d, 1H, *J* = 8.1 Hz, H-Ar); 8.41-8.36 (m, 1H, H-Ar); 8.23 (br s, 3H, H-Ar); 8.19-8.13 (m, 3H, H-Ar); 7.93-7.88 (m, 3H, H-Ar); 7.85-7.79 (m, 3H, H-Ar).

---

[3] S. Ostrowski, S. Grzyb, A. Mikus, *Helv. Chim. Acta*, **2007**, *90*, 2000.

MS (ESI) m/z (% rel. int.): 825 (3); 824 (8); 823 (18); 822 (32); 821 (62); 820 (61); 819 (100), 818 (37); 817 (51) (isotope $[M]^+$).

HRMS (ESI): $C_{44}H_{23}N_5O_2Cl_4Mg^+$ $[M]^+$ (*m/z*): calc. 817.0456; found 817.0446.

UV-VIS ($CHCl_3$) λ_{max} [nm] (log ε): 605.4 (4.05); 564.6 (4.47); 427.2 (5.59, Soret band).

*[5-(3-chloro-4-nitrophenyl)-10,15,20-tris(3-chlorophenyl)porphyrinato] copper(II) (**2h**)*

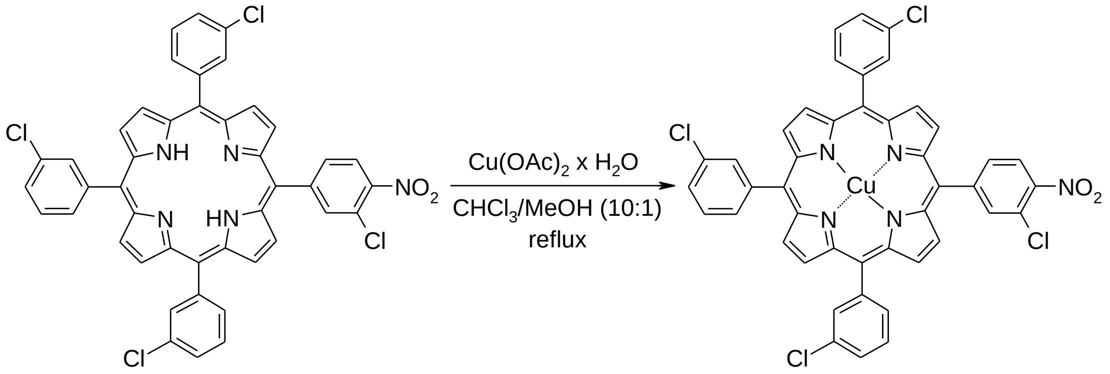

To a 50 mL round bottomed flask equipped with a reflux condenser 30.0 mg of porphyrin **2a** (0.038 mmol), 150 mg of copper(II) acetate hydrate (0.751 mmol), 10 mL of chloroform and 1 mL of methanol were added. Mixture was stirred at reflux for 15 minutes. After cooling to r.t. solvents were evaporated off. Crude product was purified by column chromatography (eluent: chloroform/*n*-hexane 2:1) yielding pink/purple solid of porphyrin **2h**: 32.0 mg, 99%. Spectroscopic data were in agreement with those of the literature.[4]

[5-(3-chloro-4-nitrophenyl)-10,15,20-tris(3-chlorophenyl)porphyrinato] nickel(II) (**2i**)

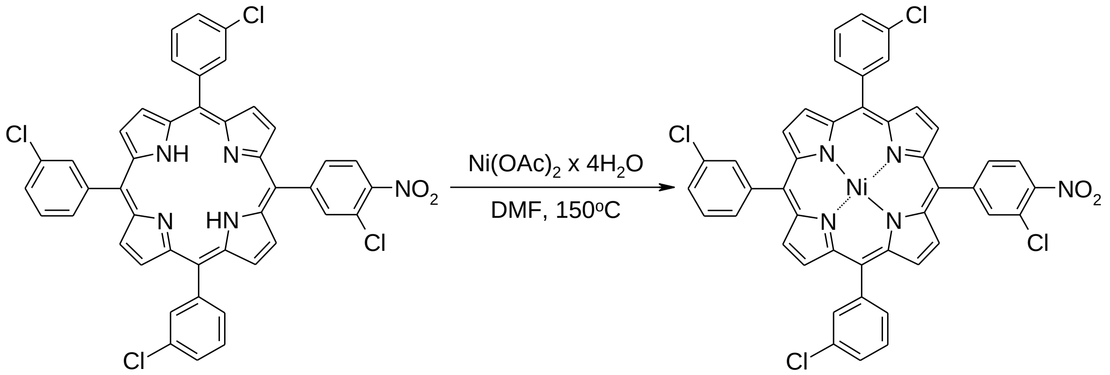

To a 10 mL sealed tube 30 mg of porphyrin **2a** (0.038 mmol), 150 mg of nickel(II) acetate tetrahydrate (0.603 mmol) and 3 mL of DMF were added. Mixture was stirred at 150°C for 1h. After cooling to r.t. mixture was poured into 30 mL of brine. Formed precipitate was filtered and washed with water 2x20 mL then dried over air for 18 hours. Crude product was purified

[4] S. Ostrowski, A. Mikus, B. Łopuszyńska, *Tetrahedron*, **2004**, *60*, 11951.

by column chromatography (eluent: chloroform/*n*-hexane, 1:1) yielding purple solid of porphyrin **2i**: 11.7 mg, 36%.

^{1}H NMR (500 MHz, $CDCl_3$) δ [ppm] = 8.80 (d, 2H, *J* = 5.0 Hz, $H^β$, AM system), 8.76 (s, 4H, $H^β$), 8.68 (d, 2H, *J* = 5.0 Hz, $H^β$, AM system), 8.25-8.21 (m, 2H, H-Ar), 8.08 (dd, 1H, *J* = 8.2 Hz, *J* = 1.6 Hz, H-Ar), 8.02 (m, 3H, H-Ar), 7.91-7.90 (m, 3H, H-Ar), 7.76-7.71 (m, 3H, H-Ar), 7.67-7.61 (m, 3H, H-Ar)

MS (ESI) m/z (% rel. int.): 859 (11); 858 (16); 857 (18); 856 (22); 855 (22); 854 (43); 853 (21); 852 (25) [isotope $(M+H)^+$].

HRMS (ESI): $C_{44}H_{24}N_5O_2Cl_4Ni^+$ $[M+H]^+$ (*m*/*z*): calc.: 852.0038; found: 852.0018

UV-VIS ($CHCl_3$) λ_{max} [nm] (log ε): 538.6 (0.245), 415.6 (2.755, Soret band)

5,10,15-tris(3-chloro-4-nitrophenyl)porphyrin-20-(3-chlorophenyl)porphyrin ***(2m)***

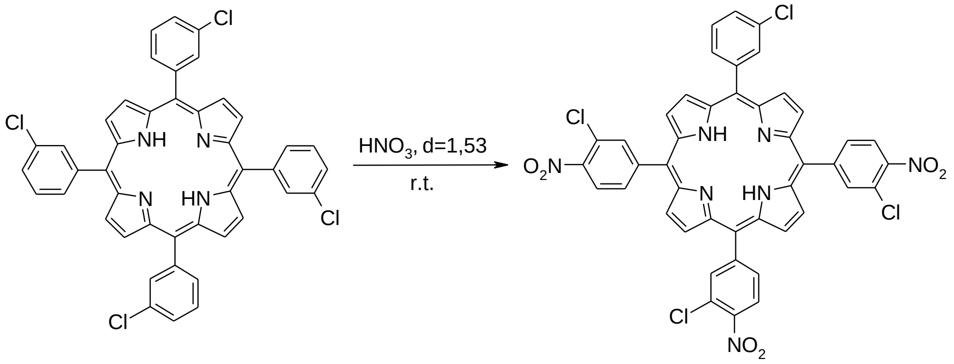

1a **2m**

The porphyrin **1a** 51.0 mg, 0.068 mmol was placed in a 10 mL round bottomed flask. Then 1.0 mL of yellow fuming nitric acid, d=1,53g/mL, (1,53g, 24,282 mmol) was added *via* syringe through a rubber septum. Reaction was stirred for 4 min at room temperature. Then mixture was diluted with 10 mL of chloroform, transferred to a separatory funnel and washed with water (3x10 mL). The organic layer was then dried over $MgSO_4/Na_2CO_3$. Then drying agent was filtered off, solvent was evaporated off and a product was separated by column chromatography (eluent: $CHCl_3$) yielding purple solid of porphyrin **2m**: 14.0 mg, 23% and other unpurified polynitroporphyrins. Spectroscopic data were in agreement with those of the literature.[4]

*5,10,15,20-tetrakis(4-fluoro-3-nitrophenyl)porphyrin (**2n**)*

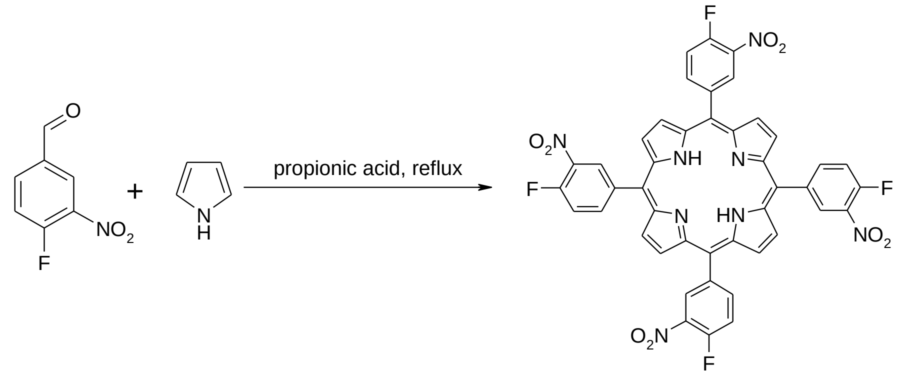

2n

In a 50 mL round bottomed flask 11.5 mL of propionic acid, 505 mg of 4-fluoro-3-nitrobenzaldehyde (2.98 mmol) and 208 mg of pyrrole (3.10 mmol) were added. Mixture was heated to reflux and stirred intensively for 1h. After that time, mixture was cooled to room temperature and 50 mL of methanol was added. The mixture was kept at room temperature for 15 min. Then precipitate was filtered off and washed with 150 mL of methanol. Product was purified by column chromatography (eluent: chloroform) yielding purple solid of porphyrin **2n**: 36.4 mg, 6%.

^{1}H NMR (500 MHz, DMSO-d_6) δ [ppm] = 9.02 (br s, 8H, H^{β}); 8.97-8.92 (m, 4H, H-Ar); 8.68-8.62 (m, 4H, H-Ar); 8.09-8.04 (m, 4H, H-Ar); -3.02 (s, 2H, 2xNH).

MS (ESI) m/z (% rel. int.): 870 (2); 869 (11); 868 (41); 867 (100) (isotope $[M+H]^+$).

HRMS (APCI): $C_{44}H_{23}N_8O_8F_4^+$ $[M+H]^+$ (*m/z*): calc. 867.1575; found 867.1567.

UV-VIS ($CHCl_3$) λ_{max} [nm] (log ε): 646.8 (3.24); 590.2 (3.74); 548.8 (4.71); 515.0 (4.24); 422.0 (5.44, Soret band).

*2,3:4,5-di-O-isopropylidene-β-D-fructopyranose (**3b**)*

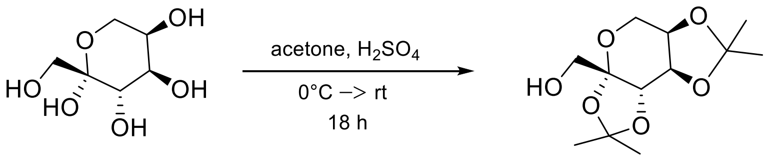

D-fructose **3b**

To a stirred solution of acetone (39 mL) and 95% H_2SO_4 (1.95 ml), D-fructose (2.0 g, 11.1 mmol) was added in portions at 0°C. The resulting mixture was left to warm to the room temperature and stirred overnight. After that time, sulfuric acid was neutralised with 10% NaOH (15 mL). Then the mixture was concentrated and extracted with methylene chloride (2x25 mL). Organic phase was washed with water (2x15 mL), dried over $MgSO_4$, filtrated and concentrated leading to a crude yellow solid (2.7 g). The pure product **3b** was obtained after recrystallization from a mixture (1:1) of petroleum ether (PE) and diethyl ether (for 2.7 g of crude product, 13.5 mL of PE and 13.5 mL of Et_2O were used). The solvents were heated to boiling then the mixture was left in a fridge for 10 min, resulting in formation of white crystals.

The solids were filtered off, washed with PE furnishing the pure **3b** (1.48 g, 51%). Spectroscopic data were in agreement with those of the literature.[5]

*1-O-methyl-2,3-O-isopropylidene-β-D-ribofuranose (**3c**)*

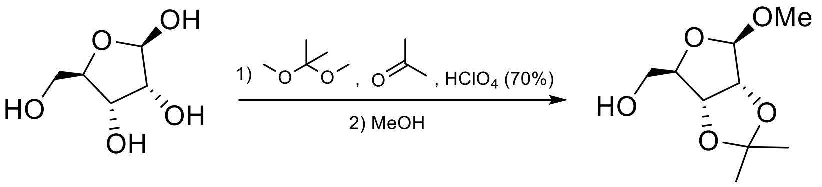

D-ribose 3c

D-Ribose (1.0 g, 6.66 mmol) was suspended in a mixture of acetone (6 mL) and 2,2-dimethoxypropane (2 mL) then stirred for 5 min at room temperature. After that time, the mixture was cooled to 0°C and $HClO_4$ (70%, 0.32 mL, 3.68 mmol) was added dropwise. The mixture was continued stirring and simultaneously warmed to a room temperature in 35 min. After that time, methanol (1.6 mL) was added and the reaction was continued for 2 hours at room temperature. Then the mixture was neutralised with a saturated solution of Na_2CO_3 (5 mL) and solids were removed by filtration. The filtrate was extracted with EtOAc (3x6 mL). The organic layer was washed with brine (4 mL), dried over $MgSO_4$, filtered and evaporated off. The crude product was purified by column chromatography (hexane→hexane/ethyl acetate, 75:25) giving pure **3c** as a colorless oil (464 mg, 34%). R_f = 0.19 (hexane/ethyl acetate, 75:25). Spectroscopic data were in agreement with those of the literature.[6]

*1,2-O-isopropylidene-α-D-xylofuranose (**3f**)*

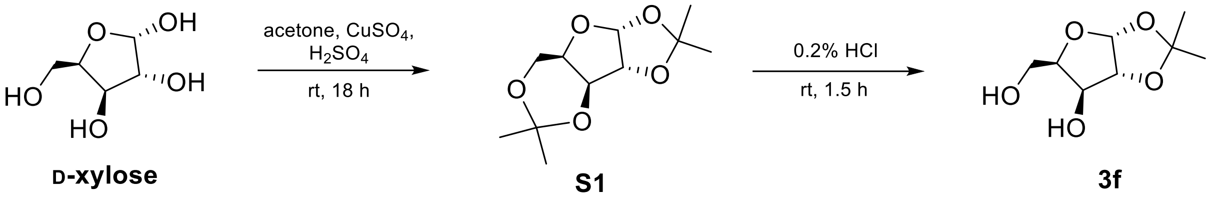

D-xylose S1 3f

Step I.

D-Xylose (5.03 g, 33.5 mmol), anhydrous $CuSO_4$ (9.36 g, 58.64 mmol) were suspended in 150 mL of acetone and stirred for 5 min. Then 1 ml of 95% H_2SO_4 (18.76 mmol) was added. The resulting mixture was stirred for 18 hours at room temperature. After that time solid Na_2CO_3 was added and the mixture was vigorously stirred for 15 min. Then solids were removed by filtration and filtrate was concentrated giving **S1** (6.19 g, 80%) with analytical purity. Spectroscopic data were in agreement with those of the literature.[7]

^{1}H NMR ($CDCl_3$, 500 MHz), δ (ppm) = 6.00 (d, J= 3.9 Hz, 1H); 4.52 (d, J= 3.6 Hz, 1H); 4.30 (d, J= 2.4 Hz, 1H); 4.10-4.02 (m, 3H); 1.50 (s, 3H); 1.45 (s, 3H); 1.39 (s, 3H); 1.33 (s, 3H).

[5] A. Yildirim, *Catal. Lett.*, **2020**, *150*, 2566

[6] J. Lv, C.-Y. Liu, Y.-F. Guo, G.-J. Feng, H. Dong, *Eur. J. Org. Chem.*, **2022**, e202101565

[7] S. Gupta, S. Bera, D. Mondal, *J. Org. Chem.*, **2020**, *85*, 2635

Step II.

6.19 g of sugar **S1** (26.87 mmol) was stirred with 62 mL 0.2% HCl for 90 minutes at room temperature. Then, the mixture was neutralized with solid $NaHCO_3$ and vigorously stirred for 10 minutes. Afterwards, the mixture was transferred to a separatory funnel and extracted with dichloromethane (3x70 mL). The combined organic phase was dried with $MgSO_4$. The drying agent was filtered off, and the filtrate was concentrated. The product was purified by column chromatography (hexane→hexane/ethyl acetate, 1:1) giving pure **3f** (1.73 g, 34%) as colorless oil. Spectroscopic data were in agreement with those of the literature.[8]

R_f = 0.16 (hexane/ethyl acetate, 1:1)

^{1}H NMR ($CDCl_3$, 500 MHz), δ (ppm) = 5.98 (d, *J* = 3.7 Hz, 1H); 4.52 (d, *J* = 3.6 Hz, 1H); 4.33 (t, *J* = 3.0 Hz, 1H); 4.20 – 4.11 (m, 2H); 4.05 (ddd, *J* = 12.4, 7.8, 2.2 Hz, 1H); 3.94 (dd, *J* = 3.7, 1.1 Hz, 1H); 1.49 (s, 3H); 1.32 (s, 3H).

[8] S. B. Ferreira, A. C. R. Sodero, M. F. C. Cardoso, E. S. Lima, C. R. Kaiser, F. P. Silva, Jr., V. F. Ferreira, J. *Med. Chem.*, **2010**, *53*, 2364

Characterization and procedures for the synthesis of porphyrin-sugar hybrids

General procedure for synthesis of porphyrin-sugar hybrids

In a sealed tube (6 mL) porphyrin (0,013 mmol, 1.0 eq), sugar substrate (0.026 mmol, 2.0 eq), 0.35 mL of triethylamine and then 29.5 mg of potassium *tert*-butoxide (0.260 mmol, 20.0 eq) were added. The reaction mixture was stirred for 4 h at 65°C. Then it was cooled to r.t and transferred to a separatory funnel with 20 mL of chloroform. Organic layer was washed with water 3x20 mL and dried with anhydrous $MgSO_4$. The drying agent was filtered off, then solvent was evaporated on rotary evaporator. Crude product was purified by column chromatography using the indicated eluent.

*5-[3-(1,2:3,4-di-O-isopropylidene-α-D-galactopyranosyl-6-oxy)-4-nitrophenyl]-10,15,20-tris(3-chlorophenyl)porphyrin (**4a**)*

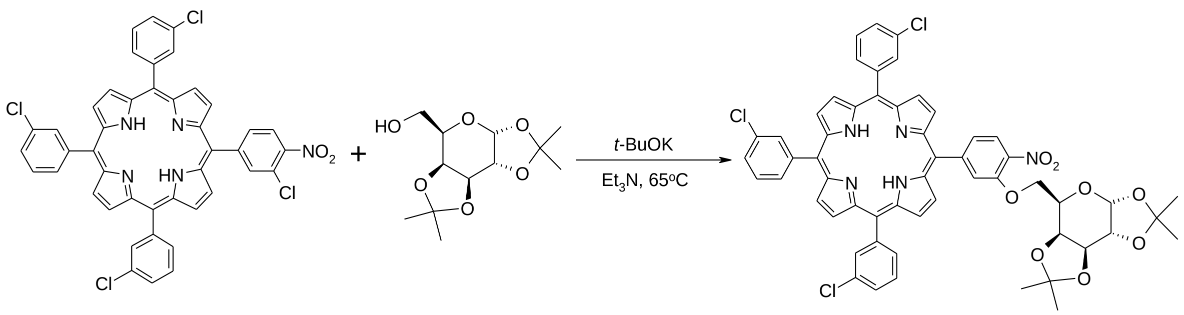

2a **3a** **4a**

Following general procedure for the synthesis of porphyrin-sugar hybrids. Starting from 10.3 mg of porphyrin **2a** (0.013 mmol), 6.8 mg of sugar **3a** (0.026 mmol), 0.35 mL of triethylamine and 29.5 mg of potassium *tert*-butoxide (0.263 mmol). The work-up performed as in general procedure. Final product was isolated by column chromatography using $CHCl_3$/MeOH 99:1 as an eluent giving purple solid of porphyrin **4a** (9.8 mg, 74%).

^{1}H NMR (500 MHz, $CDCl_3$) δ [ppm] = 8.94-8.84 (m, 8H, H^β); 8.28 (d, J = 7.9 Hz, 1H, H-Ar); 8.24 (s, 3H, H-Ar); 8.12 (d, J = 7.6 Hz, 3H, H-Ar); 8.04 (br s, 1H, H-Ar); 7.91 (d, J = 7.7 Hz, 1H, H-Ar); 7.84-7.79 (m, 3H, H-Ar); 7.75-7.68 (m, 3H, H-Ar); 5.51 (d, J = 4.7 Hz, 1H, H-gal); 4.70 (dd, J = 8.0 Hz, J = 2.4 Hz, 1H, H-gal); 4.50 (d, J = 8.0 Hz, 1H, H-gal); 4.45-4.37 (m, 3H, H-gal); 4.35 (dd, J = 4.8 Hz, J = 2.5 Hz, 1H, H-gal); 1.63 (s, 3H, CH_3), 1.36 (s, 3H, CH_3); 1.34-1.30 (m, 6H, 2x CH_3); -2.85 (s, 2H).

^{13}C NMR (125 MHz, $CDCl_3$) δ [ppm] = 150.8; 148.7; 143.7; 143.6; 139.6; 134.5; 133.2; 132.8; 128.5; 128.4; 128.2; 127.1; 124.2; 121.6; 119.4; 119.3; 119.2; 117.7; 109.7; 109.2; 96.4; 77.4; 71.0; 70.8; 70.7; 68.7; 66.6; 26.3; 26.0, 25.1, 24.5.

MS (ESI) m/z (% rel. int.): 1026 (4); 1025 (13); 1024 (33); 1023 (42); 1022 (100); 1021 (40); 1020 (81) (isotope $[M+H]^+$).

HRMS (ESI): $C_{56}H_{45}N_5O_8Cl_3^+$ $[M+H]^+$ (*m/z*): calc. 1020.2334; found 1020.2334.

UV-VIS ($CHCl_3$) λ_{max} [nm] (log ε): 644.6 (3.13); 588.4 (3.59); 549.6 (3.67); 515.0 (4.14); 419.2 (5.33, Soret band).

*5-[3-(1,2:3,4-di-O-isopropylidene-α-D-galactopyranosyl-6-oxy)-4-nitrophenyl]-10,15,20-tris(3-fluorophenyl)porphyrin (**4b**)*

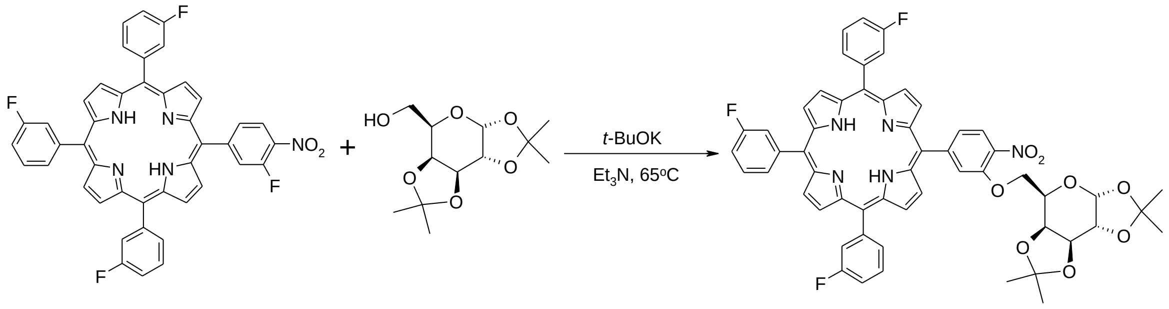

2b **3a** **4b**

Following general procedure for the synthesis of porphyrin-sugar hybrids. Starting from 9.5 mg of porphyrin **2b** (0.013 mmol), 6.8 mg of sugar **3a** (0.026 mmol), 0.35 mL of triethylamine and 29.5 mg of potassium *tert*-butoxide (0.263 mmol). The work-up performed as in general procedure. Final product was isolated by column chromatography using $CHCl_3$/MeOH 98:2 as an eluent giving purple solid of porphyrin **4b** (9.5 mg, 75%).

^{1}H NMR (500 MHz, $CDCl_3$) δ [ppm] = 8.95-8.85 (m, 8H); 8.28 (d, J = 8.1 Hz, 1H, H-Ar); 8.07-8.01 (m, 4H, H-Ar); 8.00-7.95 (m, 3H); 7.92 (d, 1H, J = 8.0Hz, H-Ar); 7.78-7.71 (m, 3H, H-Ar); 7.57-7.51 (m, 3H); 5.52 (d, 1H, J = 4.9 Hz, H-gal); 4.70 (dd, J = 8.0 Hz, J = 2.5 Hz, 1H, H-gal); 4.51 (d, 1H, J = 8.0 Hz, H-gal), 4.45-4.39 (m, 3H); 4.36 (dd, 1H, J = 4.9 Hz, J = 2.5 Hz, H-gal), 1.64 (s, 3H, CH_3); 1.37 (s, 3H, CH_3); 1.33 (s, 3H, CH_3); 1.32 (s, 3H, CH_3); -2.83 (s, 2H, NH)

^{19}F NMR (471 MHz, $CDCl_3$) δ [ppm] = -[115.0 –114.7] (m, 3F)

^{13}C NMR (125 MHz, $CDCl_3$) δ [ppm] = 161.5 (d, J = 247.4 Hz); 150.8; 148.7; 144.1-143.9 (m); 143.9; 139.6; 137.4; 132.5; 130.7; 128.5-128.2 (m); 127.1; 124.2; 121.8; 121.7; 121.6; 119.5 (d, J = 1.8 Hz); 119.4-119.2 (m); 117.6; 115.3 (d, J = 21.1 Hz); 109.7; 109.2; 96.4; 71.0; 70.8; 70.7; 68.7; 66.6; 26.3; 26.0; 25.1; 24.5.

MS (ESI) m/z (% rel. int.): 976 (1); 975 (4); 974 (16); 973 (57); 972 (100) (isotope $[M+H]^+$);

996 (1); 995 (5); 994 (7) (isotope $[M+Na]^+$).

HRMS (ESI): $C_{56}H_{45}N_5O_8F_3^+$ $[M+H]^+$ (*m/z*): calc. 972.3220; found 972.3232.

UV-VIS ($CHCl_3$) λ_{max} [nm] (log ε): 644.0 (2.59); 588.6 (3.54); 548.8 (3.63); 514.4 (4.22); 419.0 (5.54, Soret band).

*5-[3-(1,2:3,4-di-O-isopropylidene-α-D-galactopyranosyl-6-oxy)-4-nitrophenyl]-10,15,20-tris(3-bromophenyl)porphyrin (**4c**)*

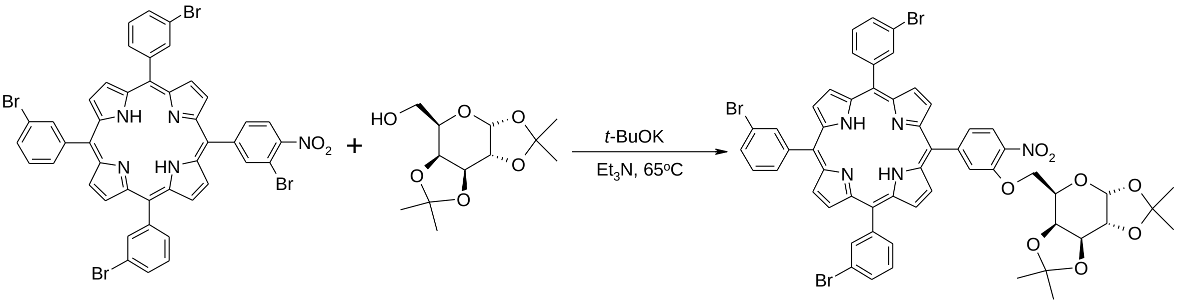

2c **3a** **4c**

Following general procedure for the synthesis of porphyrin-sugar hybrids. Starting from 12.7 mg of porphyrin **2c** (0.013 mmol), 6.8 mg of sugar **3a** (0.026 mmol), 0.35 mL of triethylamine and 29.5 mg of potassium *tert*-butoxide (0.263 mmol). The work-up performed as in general procedure. Final product was isolated by column chromatography using $CHCl_3$/MeOH 99:1 as an eluent giving purple solid of porphyrin **4c** (6.2 mg, 41%).

^{1}H NMR (500 MHz, $CDCl_3$) δ [ppm] = 8.92-8.84 (m, 8H, H^{β}); 8.39 (br s, 3H, H-Ar); 8.28 (d, 1H, J = 8.1 Hz, H-Ar); 8.16 (m, 3H, H-Ar); 8.03 (br s, 1H, H-Ar); 7.98-7.94 (m, 3H, H-Ar); 7.93-7.88 (m, 1H, H-Ar); 7.68-7.62 (m, 3H, H-Ar), 5.52-5.48 (m, 1H, H-gal); 4.69 (dd, 1H, J = 8.0 Hz, J = 2.5 Hz, H-gal); 4.49 (d, 1H, J = 8.0 Hz, H-gal); 4.44-4.36 (m, 3H, H-gal); 4.34 (dd, J = 4.8 Hz, J = 2.4 Hz, 1H, H-gal), 1.62 (s, 3H, CH_3); 1.35 (s, 3H, CH_3); 1.32-1.28 (m, 6H, 2xCH_3); -2.88 (s, 2H, NH).

^{13}C NMR (125 MHz, $CDCl_3$) δ [ppm] = 150.8; 148.7; 143.9; 139.6; 137.3; 133.3; 131.4; 131.4; 128.4; 127.9; 127.1; 124.2; 121.6; 121.4; 119.4; 119.2; 119.2; 117.7; 114.2; 109.7; 109.2; 96.4; 71.0; 70.8; 70.7; 68.7; 66.6; 26.3; 26.0; 25.1; 24.5.

MS (ESI) m/z (% rel. int.): 1160 (7); 1159 (22); 1158 (45); 1157 (52); 1156 (100); 1155 (55); 1154 (94); 1153 (17); 1152 (26) (isotope $[M+H]^+$);

1181 (5); 1180 (8); 1179 (13); 1178 (22); 1177 (14); 1176 (20); 1175 (5); 1174 (7) (isotope $[M+Na]^+$).

HRMS (ESI): $C_{56}H_{45}N_5O_8Br_3^+$ $[M+H]^+$ (*m/z*): calc. 1152.0818; found 1152.0801.

UV-VIS ($CHCl_3$) λ_{max} [nm] (log ε): 645.0 (3.24); 589.8 (3,68); 550.6 (3.79); 515.4 (4.27); 419.8 (5.46, Soret band).

*5-[3-(1,2:3,4-di-O-isopropylidene-α-D-galactopyranosyl-6-oxy)-5-methoxy-4-nitrophenyl]-10,15,20-tris(3-fluoro-5-methoxyphenyl)porphyrin (**4d**)*

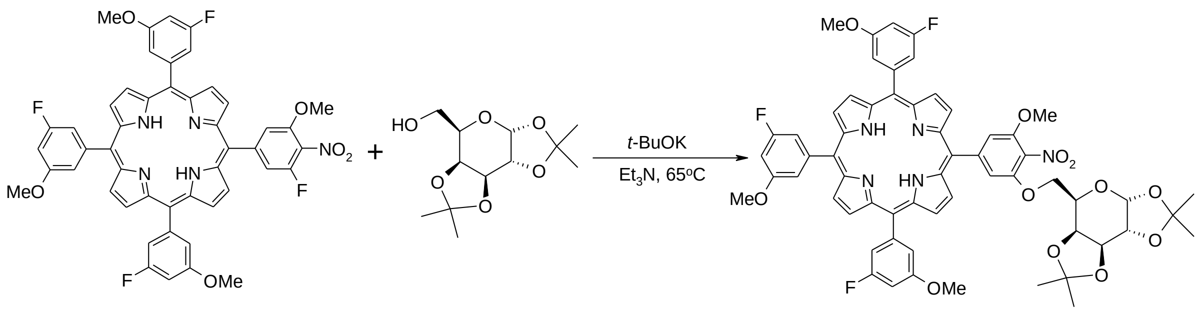

2d **3a** **4d**

Following general procedure for the synthesis of porphyrin-sugar hybrids. Starting from 11.1 mg of porphyrin **2d** (0.013 mmol), 6.8 mg of sugar **3a** (0.026 mmol), 0.35 mL of triethylamine and 29.5 mg of potassium *tert*-butoxide (0.263 mmol). The work-up performed as in general procedure. Final product was isolated by column chromatography using $CHCl_3$/MeOH 98:2 as an eluent giving purple solid of porphyrin **4d** (8.9 mg, 63%).

^{1}H NMR (500 MHz, $CDCl_3$) δ [ppm] = 8.97-8.88 (m, 8H, H^β); 7.61-7.54 (m, 7H, H-Ar); 7.52 (s, 1H, H-Ar); 7.13-7.07 (m, 3H, H-Ar); 5.47 (d, 1H, J = 4.9 Hz, H-gal); 4.64 (dd, 1H, J = 8.0 Hz, J = 2.3 Hz, H-gal); 4.42-4.30 (m, 4H, H-gal); 4.29-4.24 (m, 1H, H-gal); 4.02-3.98 (m, 12H, 4xO-CH_3); 1.56 (s, 3H, CH_3); 1.33 (s, 3H, CH_3); 1.32-1.29 (m, 6H, 2xCH_3); -2,89 (s, 2H, NH).

^{13}C NMR (125 MHz, $CDCl_3$) δ [ppm] = 162.0 (d, J = 246.1 Hz); 159.3-159.1 (m); 149.8 (J = 96.7 Hz); 145.6; 144.5-144.2 (m); 132.5-130.0 (m), 132.1; 119.4 (d, J = 2.3 Hz); 119.4-119.2 (m); 118.2; 117.3-116.9 (m); 114.8 (d, J = 22.3 Hz); 112.6 (d, J = 125.1Hz); 109.6; 109.1; 101.9-101.4 (m); 96.3; 70.8; 70.7; 70.6; 48.5; 68.4; 66.3; 57.0; 56.0; 26.3; 26.00; 25.1; 24.4.

MS (ESI) m/z (% rel. int.): 1095 (2); 1094 (15); 1093 (59); 1092 (100); (isotope $[M+H]^+$).

HRMS (ESI): $C_{60}H_{53}N_5O_{12}F_3^+$ $[M+H]^+$ (*m/z*): calc. 1092.3643; found 1092.3661.

UV-VIS ($CHCl_3$) λ_{max} [nm] (log ε): 642.4 (3.69); 588.2 (4.14); 546.8 (4.08); 513.4 (4.66); 419.0 (5.95, Soret band).

*5-[3-(1,2:3,4-di-O-isopropylidene-α-D-galactopyranosyl-6-oxy)-5-methyl-4-nitrophenyl]-10,15,20-tris(3-fluroro-5-methylphenyl)porphyrin (**4e**)*

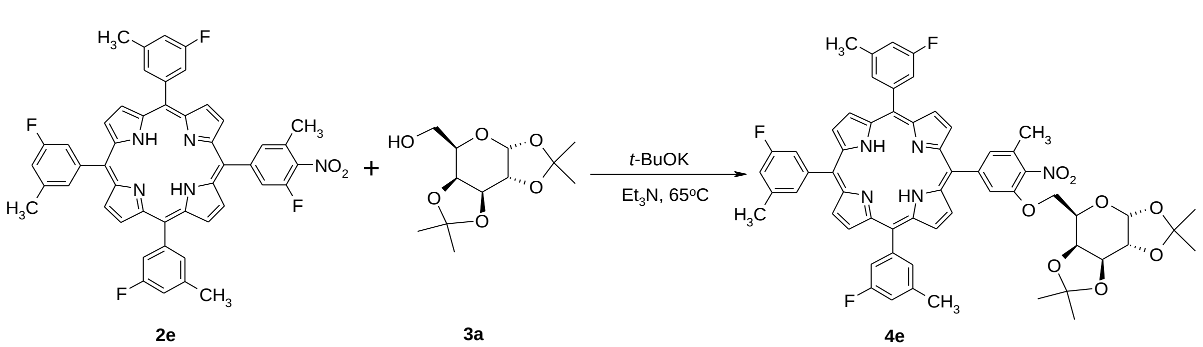

2e **3a** **4e**

Following the general procedure for the synthesis of porphyrin-sugar hybrids. Starting from 10.2 mg of porphyrin **2e** (0.013 mmol), 6.8 mg of sugar **3a** (0.026 mmol), 0.35 mL of triethylamine and 29.5 mg of potassium *tert*-butoxide (0.263 mmol). The work-up performed as in general procedure. Final product was isolated by column chromatography using $CHCl_3$/MeOH, 99:1 as an eluent giving purple solid of porphyrin **4e** (9.2 mg, 69%).

^{1}H NMR (500 MHz, $CDCl_3$) δ [ppm] = 8.92-8.85 (m, 8H, H^β); 7.85-7.79 (m, 4H, H-Ar); 7.78-7.70 (m, 4H, H-Ar); 7.37-7.32 (m, 3H, H-Ar); 5.47 (d, 1H, *J* = 4.9 Hz, H-gal); 4.68-4.63 (m, 1H, H-gal); 4.43-4.30 (m, 4H, H-gal); 4.29-4.24 (m, 1H, H-gal); 2.69-2.64 (m, 9H, CH_3); 2.61 (s, 3H, CH_3); 1.58-1.55 (m, 3H, CH_3); 1.33 (s, 3H, CH_3); 1.32-1.28 (m, 6H, CH_3); -2.88 (s, 2H, NH).

^{19}F NMR (471 MHz, $CDCl_3$) δ [ppm] = -[121.2 – 120.9] (m, 3F).

^{13}C NMR (125 MHz, $CDCl_3$) δ [ppm] = 161.5; (d, *J* = 246.7 Hz); 148.6; 145.1; 143.8-143.5 (m); 142.0; 138.7-138.5 (m) 131.7; 129.7-129.4 (m); 119.6-119.5 (m); 119.5-119.3 (m); 119.0 (d, *J* = 21.1 Hz); 118.3; 118.0; 116.0-115.6 (m); 109.6; 109.1; 96.4; 70.9; 70.8; 70.7; 68.4; 66.4; 26.3; 26.0; 25.1; 24.4; 21.7; 17.6.

MS (ESI) m/z (% rel. int.): 1031 (1); 1030 (12); 1029 (55); 1028 (100) (isotope $[M+H]^+$).

HRMS (ESI): $C_{60}H_{53}N_5O_8F_3^+$ $[M+H]^+$ (*m/z*): calc. 1028.3846; found 1028.3862.

UV-VIS ($CHCl_3$) λ_{max} [nm] (log ε): 644.2 (3.24); 589.4 (3.61); 548.4 (3.66); 514.4 (4.16); 417.4 (5.30, Soret band).

*[5-[3-(1,2:3,4-di-O-isopropylidene-α-D-galactopyranosyl-6-oxy)-4-nitrophenyl]-10,15,20-tris(3-chlorophenyl)porphyrinato] zinc(II) (**4f**)*

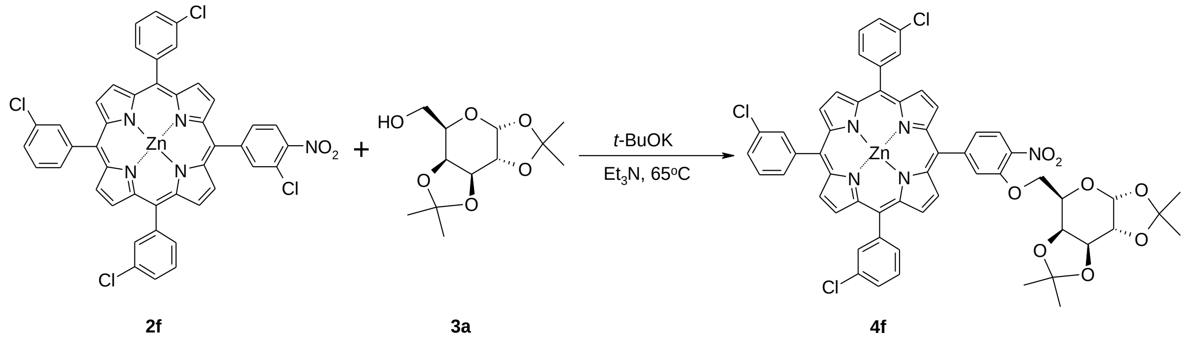

2f **3a** **4f**

Following the general procedure for the synthesis of porphyrin-sugar hybrids. Starting from 11.2 mg of porphyrin **2f** (0.013 mmol), 6.8 mg of sugar **3a** (0.026 mmol), 0.35 mL of triethylamine and 29.5 mg of potassium *tert*-butoxide (0.263 mmol). The work-up performed as in general procedure. Final product was isolated by column chromatography using $CHCl_3$/MeOH, 98:2 as an eluent giving purple solid of porphyrin **4f** (10.4 mg, 74%).

^{1}H NMR (500 MHz, $CDCl_3$) δ [ppm] = 9.00-8.92 (m, 8H, H^β); 8.28 (m, 1H, H-Ar); 8.22 (s, 3H, H-Ar); 8.14-8.08 (m, 3H, H-Ar); 8.03-7.98 (m, 1H, H-Ar); 7.95-7.89 (m, 1H, H-Ar); 7.82-7.77 (m, 3H, H-Ar); 7.73-7.66 (m, 3H, H-Ar); 5.41-5.36 (m, 1H, H-gal); 4.65 (dd, 1H, *J* = 8.0

Hz, J = 2.1 Hz, H-gal); 4.49-4.44 (m, 1H, H-gal); 4.39-4.30 (m, 3H, H-gal); 4.30-4.26 (m, 1H, H-gal); 1.37 (s, 3H, CH_3); 1.32-1.25 (m, 9H, $3xCH_3$).

^{13}C NMR (125 MHz, $CDCl_3$) δ [ppm] = 150.7; 150.7; 150.6; 150.4; 150.4; 150.3; 150.3; 150.2; 149.6; 149.5; 149.5; 144.4; 144.3; 139.4; 134.4; 134.4; 133.1; 133.0; 132.7; 132.6; 132.5; 132.4; 131.9; 131.8; 128.2; 128.0; 128.0; 127.0; 124.0; 121.5; 121.4; 120.3; 120.2; 120.2; 118.6; 109.6; 109.1; 96.3; 70.9; 70.8; 70.7; 68.7; 68.6; 68.6; 66.5; 26.2; 26.0; 25.0; 24.5.

MS (ESI) m/z (% rel. int.): 1112 (20); 1111 (28); 1110 (44); 1109 (50); 1108 (88); 1107 (62); 1106 (100); 1105 (35); 1104 (50) (isotope $[M+Na]^+$);

1090 (10); 1089 (14); 1088 (29); 1087 (45); 1086 (59); 1085 (68); 1084 (62); 1083 (71); 1082 (38); 1081 (35), (isotope $[M]^+$ and $[M+H]^+$).

HRMS (ESI): $C_{56}H_{42}N_5O_8Cl_3Zn^+$ $[M+H]^+$ (*m/z*): calc. 1081.1390; found 1081.1418.

UV-VIS ($CHCl_3$) λ_{max} [nm] (log ε): 595.8 (3.78); 555.8 (4.40); 425.0 (5.54, Soret band).

*[5-[3-(1,2:3,4-di-O-isopropylidene-α-D-galactopyranosyl-6-oxy-)-4-nitrophenyl]-10,15,20-tris(3-chlorophenyl)porphyrinato] magnesium (II) (**4g**)*

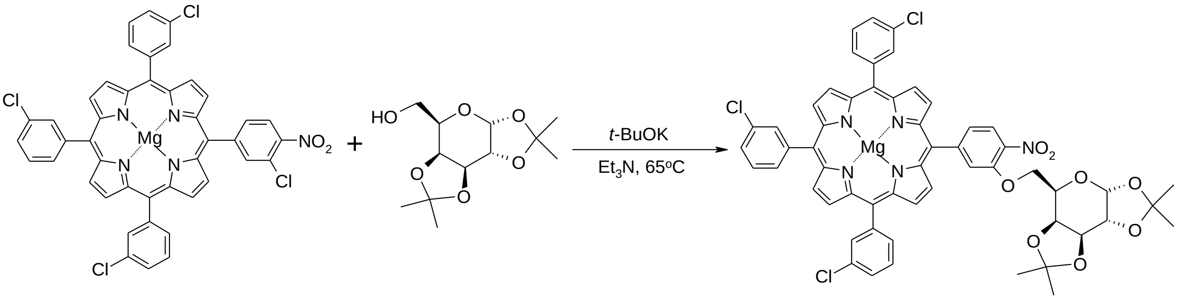

2g **3a** **4g**

Following the general procedure for the synthesis of porphyrin-sugar hybrids. Starting from 10.7 mg porphyrin **2g** (0.013 mmol), 6.8 mg of sugar **3a** (0.026 mmol), 0.35 mL of triethylamine and 29.5 mg of potassium *tert*-butoxide (0.263 mmol). The work-up performed as in general procedure. Final product was isolated by column chromatography using CH_2Cl_2/MeOH/Et_3N, 100:2:1, as an eluent giving purple solid of porphyrin **4g** (5.3 mg, 39%).

^{1}H NMR (500 MHz, DMSO-d_6) δ [ppm] = 8.91-8.89 (m, 1H, H^β); 8.87 (d, 1H, J = 4.5 Hz, H^β); 8.81-8.76 (m, 6H, H^β); 8.29 (d, 1H, J = 8.2 Hz, H-Ar); 8.25-8.12 (m, 7H, H-Ar); 7.94-7.88 (m, 4H, H-Ar); 7.85-7.79 (m, 3H, H-Ar); 5.47-5.43 (m, 1H, H-gal); 4.66-4.61 (m, 1H, H-gal); 4.55-4.49 (m, 1H, H-gal); 4.42-4.34 (m, 3H, H-gal); 4.25-4.19 (m, 1H, H-gal); 1.48-1.44 (m, 3H, CH_3); 1.28 (s, 3H, CH_3); 1.24-1.21 (m, 3H, CH_3); 1.19 (s, 3H, CH_3).

^{13}C NMR (125 MHz, DMSO-d_6) δ [ppm] = 149.5; 149.5; 149.1; 149.0; 149.0; 148.8; 148.6; 145.0; 139.0; 133.6; 132.9; 132.0; 132.0; 131.9; 131.8; 131.5; 128.3; 127.6; 127.2; 123.2; 120.1; 120.0; 120.0; 119.2; 108.6; 108.1; 95.6; 70.3; 69.9; 69.8; 68.8; 68.8; 66.4; 66.3; 25.8; 25.8; 24.9; 24.2.

MS (ESI) m/z (% rel. int.): 1071 (6); 1070 (13); 1069 (26); 1068 (46); 1067 (59); 1066 (100); 1065 (50); 1064 (69) (isotope $[M+Na]^+$).

HRMS (ESI): $C_{56}H_{42}N_5O_8Cl_3MgNa^+$ $[M+Na]^+$ (*m/z*): calc. 1064.1847; found 1064.1851.

UV-VIS ($CHCl_3$) λ_{max} [nm] (log ε): 604.0 (3.76); 564.6 (4.16); 427.2 (5.51, Soret band).

[5-[3-(1,2:3,4-di-O-isopropylidene-α-D-galactopyranosyl-6-oxy)-4-nitrophenyl]-10,15,20-tris(3-chlorophenyl)porphyrinato] copper(II) (***4h***)

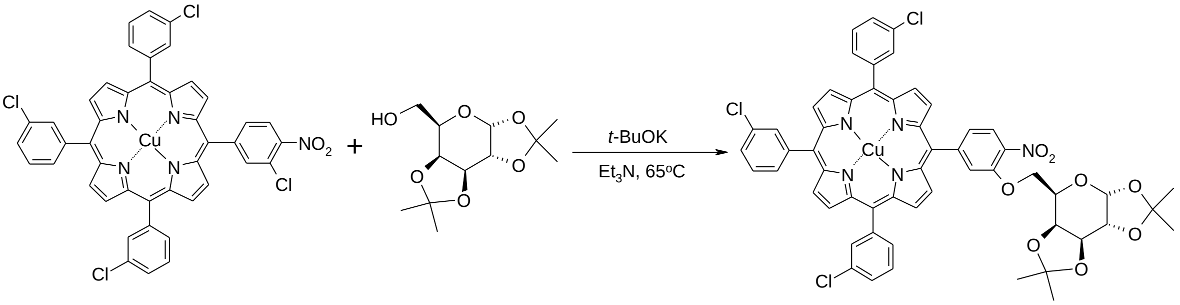

2h **3a** **4h**

Following the general procedure for the synthesis of porphyrin-sugar hybrids. Starting from 11.2 mg porphyrin **2h** (0.013 mmol), 6.8 mg of sugar **3a** (0.026 mmol), 0.35 mL of triethylamine and 29.5 mg of potassium *tert*-butoxide (0.263 mmol). The work-up performed as in general procedure. Final product was isolated by column chromatography using $CHCl_3$/MeOH, 99:1 as an eluent giving purple solid of porphyrin **4h** (3.8 mg, 27%).

^{1}H NMR and **^{13}C NMR** spectra were not measured due to paramagnetic properties of compound **4h**.

MS (ESI) m/z (% rel. int.): 1111 (5); 1110 (7); 1109 (20); 1108 (32); 1107 (65); 1106 (54); 1105 (100); 1104 (42); 1103(66) (isotope $[M+Na]^+$);

2195 (3); 2194 (5); 2193 (8); 2192 (12); 2191 (19); 2190 (24); 2189 (29); 2188 (32); 2187 (29); 2186 (20); 2185 (20); 2184 (7); 2183 (5) (isotope $[2M+Na]^+$).

HRMS (ESI): $C_{56}H_{42}N_5O_8Cl_3CuNa^+$ $[M+Na]^+$ (*m/z*): calc. 1103.1293; found 1103.1321.

UV-VIS ($CHCl_3$) λ_{max} [nm] (log ε): 539.2 (4.53); 416.4 (5.78, Soret band).

*5-[3-(2,3:4,5-di-O-isopropylidene-β-D-fructopyranosyl-1-oxy)-4-nitrophenyl]-10,15,20-tris(3-chlorophenyl)porphyrin (**4j**)*

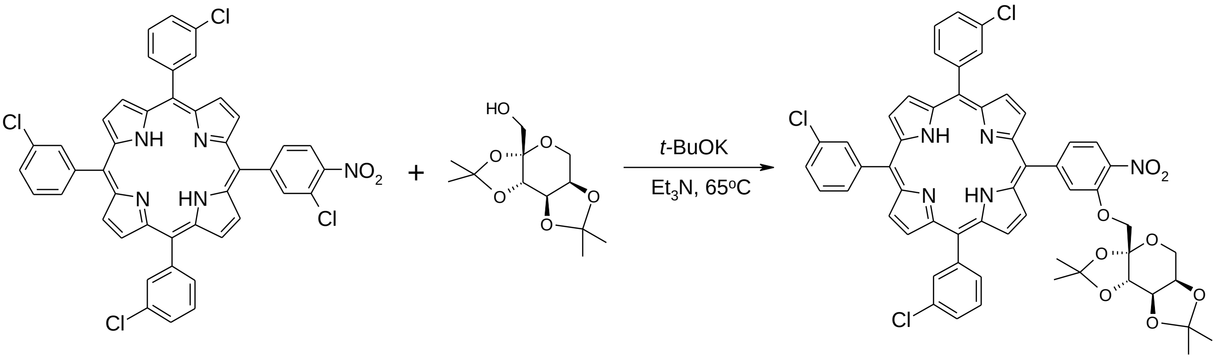

2a **3b** **4j**

Following the general procedure for the synthesis of porphyrin-sugar hybrids. Starting from 10.3 mg of porphyrin **2a** (0.013 mmol), 6.8 mg of sugar **3b** (0.026 mmol), 0.35 mL of triethylamine and 29.5 mg of potassium *tert*-butoxide (0.263 mmol). The reaction was stirred for 18 h. The work-up performed as in general procedure. Final product was isolated by column chromatography using $CHCl_3$/MeOH 99:1 as an eluent giving purple solid of porphyrin **4j** (8.5 mg, 64%).

^{1}H NMR (500 MHz, $CDCl_3$) δ [ppm] = 8.91-8.84 (m, 8H, H^{β}); 8.26-8.21 (m, 4H, H-Ar); 8.14-8.09 (m, 3H, H-Ar); 8.06 (d, 1H, J = 5.2 Hz, H-Ar); 7.94 (d, 1H, J = 8.1Hz, H-Ar); 7.84-7.79 (m, 3H, H-Ar); 7.75-7.68 (m, 3H, H-Ar); 4.86 (br s, 1H, H-gal); 4.72 (dd, 1H, J = 8.0 Hz, J = 2.8 Hz, H-gal); 4.52-4.46 (m, 1H, H-gal); 4.33-4.26 (m, 1H, H-gal); 4.25-4.12 (m, 1H, H-gal); 3.99-3.94 (m, 1H, H-gal); 3,67 (d, 1H, J = 13.1Hz) 1.61 (s, 3H, CH_3); 1.58 (s, 3H, CH_3); 1.34-1.28 (m, 6H, 2x CH_3); -2,86 (s, 2H, NH)

^{13}C NMR (125 MHz, $CDCl_3$) δ [ppm] = 150.3; 150.3; 150.2; 148.5; 143.7; 143.6; 143.6; 139.6; 134.5; 133.2; 132.9; 128.5; 128.4; 128.1; 124.0; 121.7; 121.6; 119.5; 119.3; 119.2; 117.6; 109.7; 109.2; 109.2; 101.5; 71.0; 70.2; 70.1; 70.0; 61.6; 26.9; 26.0; 25.5; 24.0.

MS (ESI) m/z (% rel. int.): 970 (6); 969 (12); 968 (33); 967 (45); 966 (100); 965 (46); 964 (77) (isotope $[M+H]^+$).

HRMS (ESI): $C_{53}H_{41}N_5O_7Cl_3^+$ $[M+H]^+$ (m/z): calc. 964.2072; found 964.2082.

UV-VIS ($CHCl_3$) λ_{max} [nm] (log ε): 644.8 (3.40); 589.2 (3.75); 549.4 (3.81); 514.8 (4.26); 418.4 (5.39, Soret band).

*5-[4-nitro-3-(1-O-methyl-2,3-O-isopropylidene-β-D-ribofuranosyl-5-oxy)phenyl]-10,15,20-tris(3-chlorophenyl)porphyrin (**4k**)*

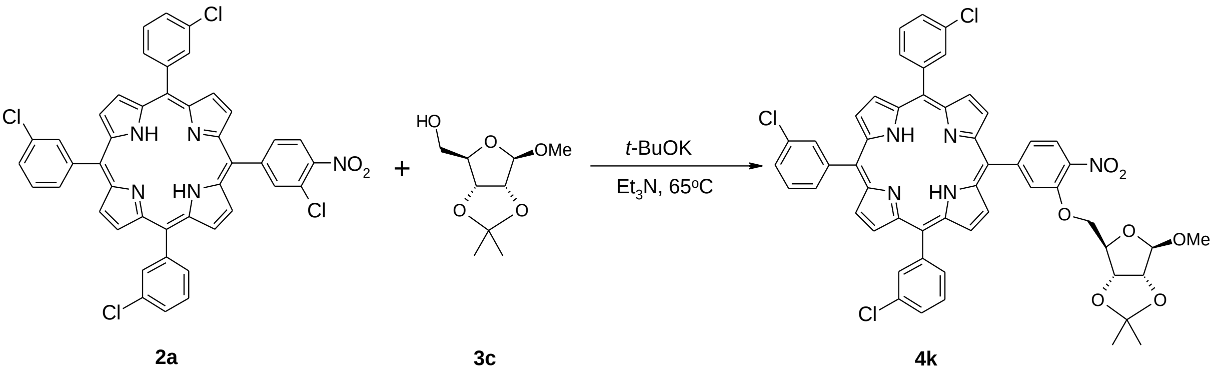

Following the general procedure for the synthesis of porphyrin-sugar hybrids. Starting from 10.3 mg of porphyrin **2a** (0.013 mmol), 5.3 mg of sugar **3c** (0.026 mmol), 0.35 mL of triethylamine and 29.5 mg of potassium *tert*-butoxide (0.263 mmol). The reaction mixture was stirred for 18 h. The work-up performed as in general procedure. Final product was isolated by column chromatography using $CHCl_3$/MeOH 99:1 as an eluent giving purple solid of porphyrin **4k** (6.5 mg, 52%).

^{1}H NMR (500 MHz, $CDCl_3$) δ [ppm] = 8.91 (d, 2H, $H^β$); 8.88 (s, 4H, $H^β$); 8.84 (d, 2H, $H^β$); 8.27-8.21 (m, 4H, H-Ar); 8.14-8.09 (m, 3H, H-Ar); 7.97-7.90 (m, 2H, H-Ar); 7.84-7.79 (m, 3H, H-Ar); 7.75-7.68 (m, 3H, H-Ar); 4.97-4.94 (m, 2H, H-ryb); 4.71-4.66 (m, 1H, H-ryb); 4.63 (d, 1H, *J* = 6.1 Hz, H-ryb); 4.34-4.27 (m, 1H, H-ryb); 4.25-4.18 (m, 1H, H-ryb); 3.16 (s, 3H, O-CH_3); 1.51 (s, 3H, CH_3); 1.36 (s, 3H, CH_3); -2.86 (s, 2H, 2xNH).

^{13}C NMR (125 MHz, $CDCl_3$) δ [ppm] = 150.1; 148.6; 143.6; 143.6; 140.0; 134.5; 133.2; 132.8; 128.5; 128.5; 128.2; 127.2; 124.1; 121.3; 119.5; 119.3; 117.4; 112.8; 110,0; 85.2; 84.2; 82.0; 70.4; 55.2; 26.6; 25.1.

MS (ESI) m/z (% rel. int.): 970 (6); 969 (12); 968 (33); 967 (45); 966 (100); 965 (46); 964 (77) (isotope $[M+H]^+$).

HRMS (ESI): $C_{53}H_{41}N_5O_7Cl_3^+$ $[M+H]^+$ (*m/z*): calc. 964.2072; found 964.2082.

UV-VIS ($CHCl_3$) λ_{max} [nm] (log ε): 644.8 (3.40); 589.2 (3,75); 549.4 (3.81); 514.8 (4.26); 418.4 (5.39, Soret band).

*5-[4-nitro-3-(2,3:4,6-di-O-isopropylidene-α-L-sorbofuranosyl-1-oxy)phenyl]-10,15,20-tris(3-chlorophenyl)porphyrin (**4l**)*

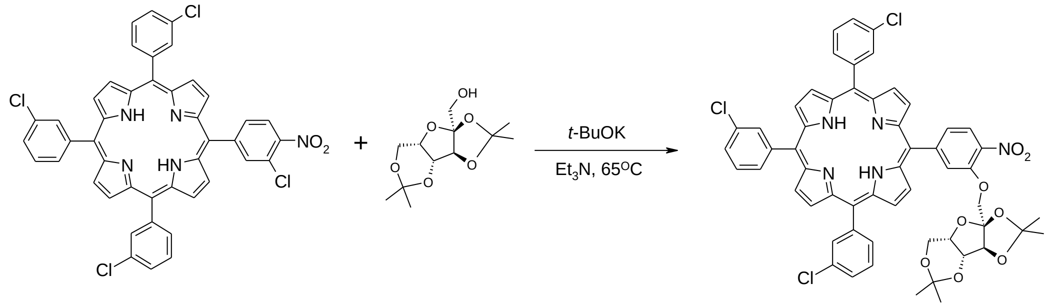

2a **3d** **4l**

Following the general procedure for the synthesis of porphyrin-sugar hybrids. Starting from 10.3 mg of porphyrin **2a** (0.013 mmol), 6.8 mg of sugar **3d** (0.026 mmol), 0.35 mL of triethylamine and 29.5 mg of potassium *tert*-butoxide (0.263 mmol). The reaction mixture was stirred for 18 h. The work-up performed as in general procedure. Final product was isolated by column chromatography using $CHCl_3$/MeOH 99:1 as an eluent giving purple solid of porphyrin **4l** (9.3 mg, 70%).

^{1}H NMR (500 MHz, $CDCl_3$) δ [ppm] = 8.94-8.86 (m, 8H, H^{β}); 8.28-8.23 (m, 4H, H-Ar); 8.16-8.10 (m, 4H, H-Ar); 7.93 (d, 1H, J = 8.1Hz, H-Ar); 7.85-7.79 (m, 3H, H-Ar); 7.76-7.68 (m, 3H, H-Ar); 4.89 (s, 1H, H-sorb); 4.71-4.63 (m, 1H, H-sorb); 4.51-4.45 (m, 1H, H-sorb); 4.44 (s, 1H, H-sorb); 4.17 (s, 1H, H-sorb); 3.99 (m, 1H, H-sorb); 3.87 (d, 1H, J = 13.7 Hz, H-sorb); 1.63 (s, 3H, CH_3); 1.58 (s, 3H, CH_3); 1.39 (s, 3H, CH_3); 1.25 (s, 3H, CH_3); -2.83 (s, 2H, NH).

^{13}C NMR (125 MHz, $CDCl_3$) δ [ppm] = 150.3; 150.2; 148.6; 143.7; 143.6; 139.5; 134.5; 134.5; 133.2; 132.8; 128.5; 128.4; 128.1; 127.3; 127.3; 124.0; 121.5; 121.4; 119.4; 119.3; 119.2; 117.6; 113.6; 112.9; 97.5; 84.2; 73.2; 72.8; 68.7; 60.4; 29.1; 28.0; 26.6; 18.6.

MS (ESI) m/z (% rel. int.): 1031 (1); 1030 (12); 1029 (55); 1028 (100) (isotope $[M+H]^+$).

HRMS (ESI): $C_{60}H_{53}N_5O_8F_3^+$ $[M+H]^+$ (*m/z*): calc. 1028.3846; found 1028.3862.

UV-VIS ($CHCl_3$) λ_{max} [nm] (log ε): 645.0 (3.60); 588.6 (3.91); 549.2 (4.19); 515.4 (4.45); 416.8 (5.35, Soret band).

*5-[4-nitro-3-(1,2-isopropylideneglyceryl-3-oxy)-phenyl]-10,15,20-tris(3-chlorophenyl)porphyrin ((rac)-**4m**)*

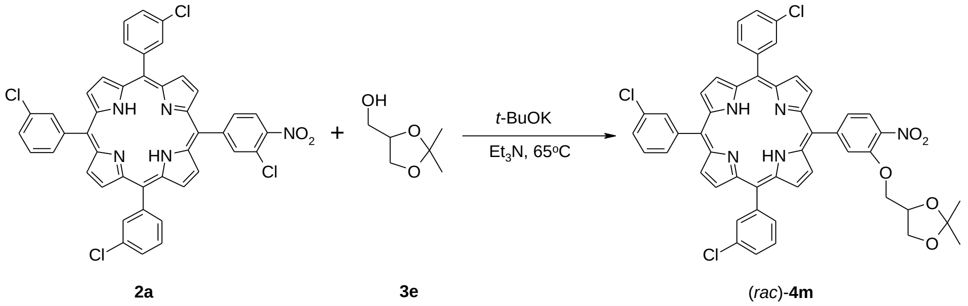

2a **3e** (*rac*)-**4m**

Following the general procedure for the synthesis of porphyrin-sugar hybrids. Starting from 10.3 mg of porphyrin **2a** (0.013 mmol), 3.4 mg of alcohol **3e** (0.026 mmol), 0.35 mL of triethylamine and 29.5 mg of potassium *tert*-butoxide (0.263 mmol). The reaction mixture was stirred for 18 h. The work-up performed as in general procedure. Final product was isolated by column chromatography using $CHCl_3$ as an eluent giving purple solid of porphyrin (*rac*)-**4m** (7.2 mg, 62%).

^{1}H NMR (500 MHz, $CDCl_3$) δ [ppm] = 8.93-8.82 (m, 8H, H^β); 8.26 (d, 1H, 8.1Hz, H-Ar); 8.23 (br s, 3H, H-Ar); 8.11 (m, 3H, H-Ar); 7.99 (br s, 1H, H-Ar); 7.93 (d, 1H, *J* = 7.7 Hz, H-Ar); 7.84-7.78 (m, 3H, H-Ar); 7.75-7.68 (m, 3H, H-Ar); 4.59-4.53 (m, 1H, H-gly); 4.36-4.29 (m, 1H, H-gly); 4.28-4.20 (m, 2H, H-gly); 4.11 (dd, 1H, *J* = 8.5 Hz *J* = 5.9 Hz, H-gly; 1.37 (s, 6H, CH_3); -2.87 (s, 2H, NH).

^{13}C NMR (125 MHz, $CDCl_3$) δ [ppm] = 150.4; 148.6; 143.6; 143.6; 139.7; 134.5; 133.2; 132.8; 128.5; 128.5; 128.2; 127.2; 124.1; 121.4; 119.5; 119.3; 117.5; 110.0; 73.8; 70.0; 66.8; 26.8; 25.5.

MS (ESI) m/z (% rel. int.): 898 (4); 897 (11); 896 (31); 895 (41); 894 (100); 893 (41); 892 (89) (isotope $[M+H]^+$).

HRMS (ESI): $C_{50}H_{37}N_5O_5Cl_3^+$ $[M+H]^+$ (*m/z*): calc. 892.1860; found 892.1852.

UV-VIS ($CHCl_3$) λ_{max} [nm] (log ε): 644.8 (3.41); 589.0 (3.74); 549.4 (3.81); 514.8 (4.26); 419.2 (5.45, Soret band).

*5-[3-(1,2:5,6-Di-O-isopropylidene-α-D-glucofuranosyl-3-oxy)-4-nitrophenyl]10,15,20-tris(3-chlorophenyl)porphyrin (**4o**)*

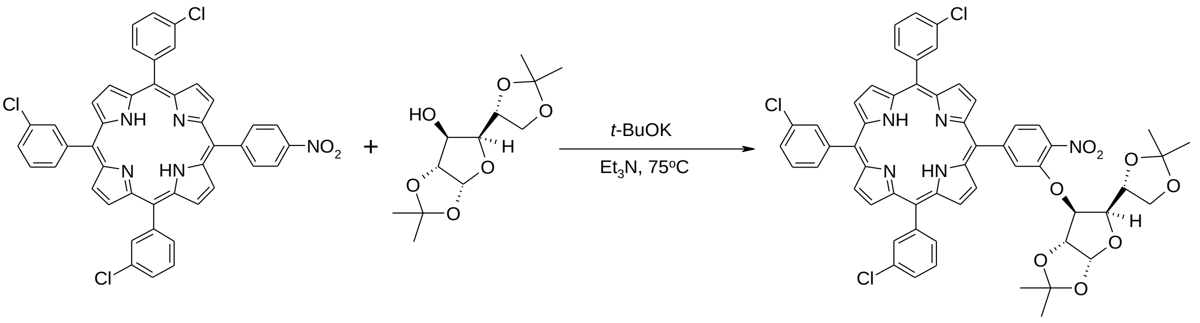

2a **3g** **4o**

Following the general procedure for the synthesis of porphyrin-sugar hybrids. Starting from 20.6 mg of porphyrin **2a** (0.026 mmol), 13.6 mg of sugar **3g** (0.052 mmol), 0.7 mL of triethylamine and 73.0 mg of potassium *tert*-butoxide (0.65 mmol). The reaction mixture was stirred for 48 h at 75°C. The work-up performed as in general procedure. Final product was isolated by column chromatography using $CHCl_3$/MeOH, 99:1 as an eluent giving purple solid of porphyrin **4o** (15.4 mg, 58%).

^{1}H NMR (500 MHz, $CDCl_3$) δ [ppm] = 8.95-8.84 (m, 8H, H^β); 8.29-8.21 (m, 4H, H-Ar); 8.17 (br s, 1H, H-Ar); 8.12 (m, 3H, H-Ar); 7.99 (d, 1H, *J* = 8.2 Hz, H-Ar); 7.85-7.80 (m, 3H, H-Ar); 7.76-7.69 (m, 3H, H-Ar); 6.10 (br s, 1H, H-glu); 5.01 (br s, 1H, H-glu); 4.93-4.88 (m, 1H, H-

glu); 4.69-4.63 (m, 1H, H-glu); 4.27-4.20 (m, 2H, H-glu); 4.12-4.07 (m, 1H, H-glu); 1.38-1.34 (m, 3H, CH_3); 1.31-1.25 (m, 3H, CH_3); 1.24-1.18 (m, 6H, 2xCH_3); -2.85 (s, 2H, NH).

^{13}C NMR (125 MHz, $CDCl_3$) δ [ppm] = 149.2; 149.2; 148.6; 143.6; 143.6; 140.4; 140.3; 134.5; 133.2; 132.8; 132.8; 128.5; 128.5; 128.2; 128.2; 128.0; 127.9; 124.2; 122.7; 122.6; 122.5; 119.6; 119.3; 119.3; 117.3; 114.2; 114.1; 112.5; 112.5; 109.7; 109.6; 105.2; 82.9; 82.8; 82.8; 82.6; 80.9; 72.2; 67.7; 27.0; 26.7; 26.3; 25.2.

MS (ESI) m/z (% rel. int.): 1027 (3); 1026 (7); 1025 (20); 1024 (36); 1023 (54); 1022 (100); 1021 (51); 1020 (87) (isotope $[M+H]^+$).

HRMS (ESI): $C_{56}H_{45}N_5O_8Cl_3^+$ $[M+H]^+$ (*m/z*): calc. 1020.2334; found 1020.2349.

UV-VIS ($CHCl_3$) λ_{max} [nm] (log ε): 645.4 (3.43); 589.4 (3.78); 550.4 (3.84); 514.8 (4.27); 420.0 (5.52, Soret band).

*5,10,15-tris[3-(1,2:3,4-di-O-isopropylidene-α-D-galactopyranosyl-6-oxy)-4-nitrophenyl]-20-(3-chlorophenyl)porphyrin (**5a**)*

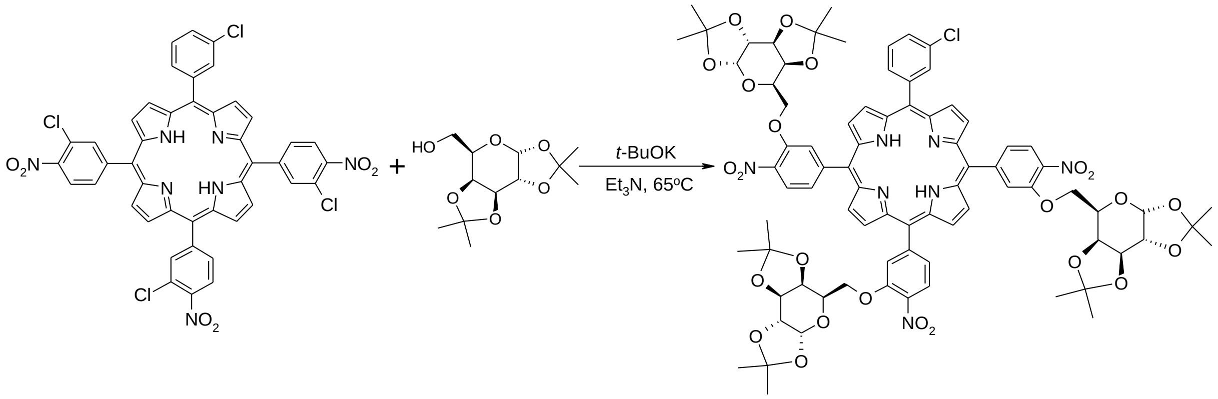

2m **3a** **5a**

Following the general procedure for the synthesis of porphyrin-sugar hybrids. Starting from 11.5 mg of porphyrin **2m** (0.013 mmol), 20.4 mg of sugar **3a** (0.078 mmol), 0.35 mL of triethylamine and 35.0 mg of potassium *tert*-butoxide (0.312 mmol). The work-up performed as in general procedure. Final product was isolated by column chromatography using $CHCl_3$/MeOH, 96:4 as an eluent giving purple solid of porphyrin **5a** (6.3 mg, 31%).

^{1}H NMR (500 MHz, $CDCl_3$) δ [ppm] = 8.94-8.83 (m, 8H, H-Ar); 8.32-8.26 (m, 3H, H-Ar); 8.22 (s, 1H, H-Ar); 8.11 (m, 1H, H-Ar); 8.01 (s, 3H, H-Ar); 7.94-7.86 (m, 3H, H-Ar); 7.83 (m, 1H, H-Ar); 7.73 (m, 1H, H-Ar); 5.51-5.46 (m, 3H, H-gal), 4.68 (m, 3H, H-gal); 4.49 (m, 3H, H-gal); 4.38 (s, 9H, H-gal); 4.35-4.31 (m 3H, H-gal); 1.61 (s, 9H, CH_3); 1.34 (s, 9H, CH_3); 1.30 (s, 18H, CH_3); -2.89 (s, 2H, NH).

^{13}C NMR (125 MHz, $CDCl_3$) δ [ppm] = 150.8; 150.8; 148.4; 148.3; 139.7; 134.5; 134.4; 133.3; 132.8; 132.8; 128.6; 128.2; 127.1; 124.2; 121.5; 121.6; 121.4; 119.9; 118.3; 118.3; 118.2; 118.1; 114.1; 109.7; 109.2; 96.4; 70.9; 70.8; 70.7; 68.7; 66.6; 26.3; 26.0; 25.1; 24.5.

MS (APCI) m/z (% rel. int.): 1563 (2); 1562 (11); 1561 (34); 1560 (72); 1559 (87); 1558 (100) (isotope $[M+H]^+$).

HRMS (APCI): $C_{80}H_{81}N_7O_{24}Cl^+$ $[M+H]^+$ (*m/z*): calc. 1558.5021; found 1558.5009.

UV-VIS ($CHCl_3$) λ_{max} [nm] (log ε): 644.6 (3.40); 590.6 (3.82); 550.8 (3.91); 515.8 (4.33); 422.6 (5.57, Soret band).

*5,10,15,20-tetrakis[4-(1,2:3,4-di-O-isopropylidene-α-D-galactopyranosyl-6-oxy)-3-nitrophenyl]porphyrin (**5b**)*

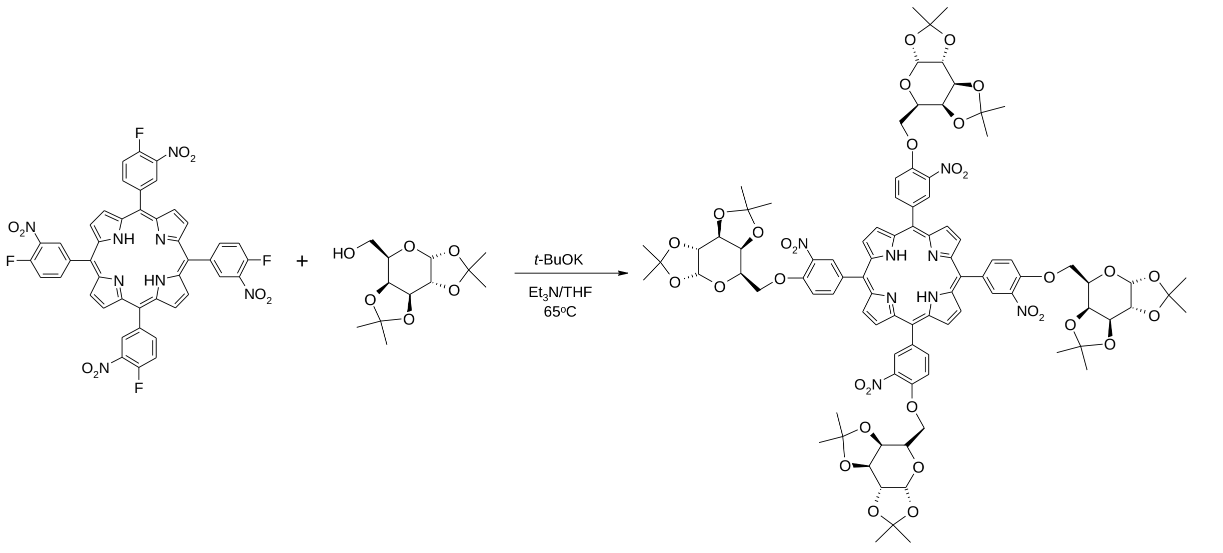

2n **3a** **5b**

Following the general procedure for the synthesis of porphyrin-sugar hybrids. Starting from 11.3 mg of porphyrin **2n** (0.013 mmol), 27.1 mg of sugar **3a** (0.104 mmol), 0.25 mL of triethylamine, 0.1 mL of tetrahydrofuran and 37.9 mg of potassium *tert*-butoxide (0.338 mmol). The work-up performed as in general procedure. Final product was isolated after two purifications by column chromatography the first one used $CHCl_3$/MeOH, 96:4 as an eluent. The analytical sample was obtained after second column chromatography (eluent: ethyl acetate/*n*-hexane 2:3) giving purple solid of porphyrin **5b** (15.3 mg, 64%).

^{1}H NMR (500 MHz, $CDCl_3$) δ [ppm] = 8.92-8.85 (m, 8H, H^β); 8.72 (s, 4H, H-Ar); 8.41-8.32 (m, 4H, H-Ar); 7.61-7.53 (m, 4H, H-Ar); 5.67 (d, 4H, J = 4.9 Hz, H-gal); 4.80 (dd, 4H, J = 7.8 Hz, J = 2.5 Hz, H-gal); 4.66-4.55 (m, 12H, H-gal); 4.50-4.42 (m, 8H, H-gal); 1.69 (s, 12H, CH_3); 1.57 (s, 12H, CH_3); 1.47 (s, 12H, CH_3); 1.43 (s, 12H, CH_3); -2.87 (s, 2H, NH).

^{13}C NMR (125 MHz, $CDCl_3$) δ [ppm] = 152.3; 139.5; 139.4; 138.6; 134.6; 130.6; 117.8; 113.8; 113.7; 109.8; 109.3; 96.6; 71.0; 71.0; 70.8; 68.7; 66.5; 26.3; 26.2; 25.2; 24.6.

MS (APCI) m/z (% rel. int.): 1832 (2); 1831 (7); 1830 (21); 1829 (51); 1828 (100); 1827 (93); (isotope $[M+H]^+$).

HRMS (APCI): $C_{92}H_{99}N_8O_{32}^+$ $[M+H]^+$ (*m/z*): calc. 1827.6365; found 1827.6353.

UV-VIS ($CHCl_3$) λ_{max} [nm] (log ε): 649.0 (3.45); 591.6 (3.72); 554.2 (3.88); 517.6 (4.23); 424.2 (5.54, Soret band).

Characterization and procedures for deprotected sugar hybrids

5-[3-(D-galactopyranosyl-6-oxy-)-4-nitro-phenyl]-10,15,20-tris-(3-chlorophenyl)porphyrin (6a)

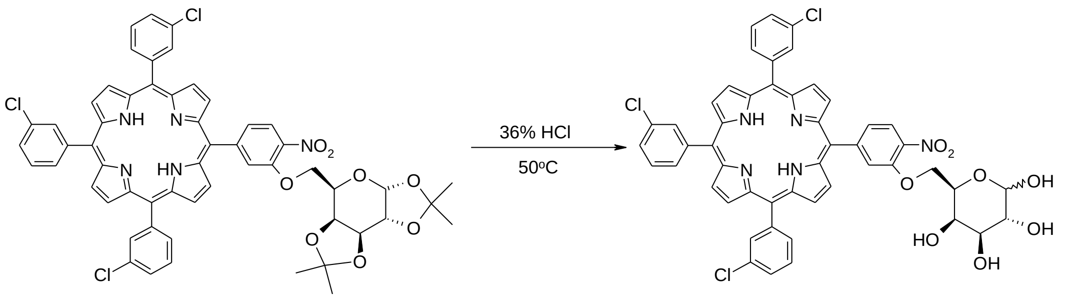

4a **6a**

In a 10 mL sealed tube 49.0 mg of porphyrin **4a** (0.052 mmol) and 3.0 mL of 36% HCl_{aq} were added. The reaction mixture was stirred vigorously at 50°C for 20 min. Mixture was cooled to r.t. and transferred to a separatory funnel with 30 mL of $CHCl_3$ and 30 mL of saturated aqueous solution of sodium carbonate. Organic phase was separated and water phase was extracted 2x10 mL of $CHCl_3$. Combined organic phases were washed with 10 mL of saturated solution of sodium carbonate and then with 10 mL of water. Solvent was evaporated off. Crude product was purified by column chromatography (eluent: $CHCl_3$/MeOH, 94:6) yielding purple solid of porphyrin **6a** (31.6 mg, 70%) as a mixture of two inseparable isomers (A:B, ratio 2.8:1).

^{1}H NMR (500 MHz, DMSO-d_6) δ [ppm] = 9.02-8.94 (m, 2H, H^{β}, isom A+B); 8.86 (br s, 6H, H^{β}, isom A+B); 8.32-8.26 (m, 3H, H-Ar, isom A+B); 8.24 (br s, 1H, H-Ar, isom A+B); 8.21-8.16 (m, 3H, H-Ar, isom A+B); 7.96-7.90 (m, 4H, H-Ar, isom A+B); 7.87-7.81 (m, 3H, H-Ar, isom A+B); 6.57 (d, 0.63H, *J* = 6,8 Hz, H-gal-isom A); 6.29 (d, 0.63H, *J* = 4.7 Hz, H-gal-isom A); 6,20 (m, 0.24H, H-gal-isom B); 6.11 (d, 0.24H, *J* = 6.3 Hz, H-gal-isom B); 5.20 (d, 0.24H, *J* = 4.6Hz, H-gal-isom B); 5.14 (d, 0.24H, *J* = 6.6 Hz, H-gal-isom B); 5.11-5.07 (m, 0.24H, H-gal-isom B); 5.05 (d, 0.24H, *J* = 6.7 Hz, H-gal-isom B); 4.91 (t, 0.63H, *J* = 4.2 Hz, H-gal-isom A); 4.85-4.79 (m, 0.48H, H-gal-isom B); 4.76 (d, 0.24H, *J* = 4.2 Hz, H-gal-isom B); 4.69 (d, 0.63H, *J* = 4.6 Hz H-gal-isom A); 4.66 (d, 0.63H, *J* = 5.6Hz, H-gal-isom A); 4.53 (d, 0.63H, *J* = 4.3 Hz, H-gal-isom A); 4.52-4.47 (m, 0.63H, H-gal-isom A); 4.44-4.35 (m, 1.50H, H-gal-isom A + H-gal-isom B); 4.34-4.25 (m, 2.52H, H-gal-isom A); 3.98-3.91 (m, 0.72H, H-gal-isom B); 3.90-3.85 (0.63H, H-gal-isom A); 3.83-3.79 (m, 0.63H, H-gal-isom A); 3.77-3.73 (m, 0.63H, H-gal-isom A); 3.68-3.58 (m, 0.96H, H-gal-isom B); 3.50-3.43 (m, 0.63H, H-gal-isom A); 3.23-3.16 (m, 0.72H, H-gal-isom B); -3.02 (s, 2H, NH isom A+B).

^{13}C NMR (125 MHz, DMSO-d_6) δ [ppm] = 150.4; 147.8; 147.7; 143.5; 134.1; 133.3; 132.5; 129.3; 128.9; 124.0; 119.3; 119.1; 92.2; 73.5; 72.7; 72.2; 69.4; 68.9.

MS (ESI) m/z (% rel. int.): 967 (16); 966 (30); 965 (44); 964 (83); 963 (50); 962 (67) (isotope $[M+Na]^+$);

946 (11); 945 (19); 944 (48); 943 (52); 942 (100); 941 (54); 940 (92) (isotope $[M+H]^+$).

HRMS (ESI): $C_{50}H_{37}N_5O_8Cl_3^+$ $[M+H]^+$ (*m/z*): calc. 940.1708; found 940.1688.

UV-VIS ($CHCl_3$) λ_{max} [nm] (log ε): 642.6 (3.45); 590.2 (3.97); 550.2 (3.97); 515.6 (4.47); 420.0 (5.74, Soret band).

*5-[3-(glycerol-1-oxy)-4-nitrophenyl]-10,15,20-tris-(3-chlorophenyl)porphyrin ((rac)-**6m**)*

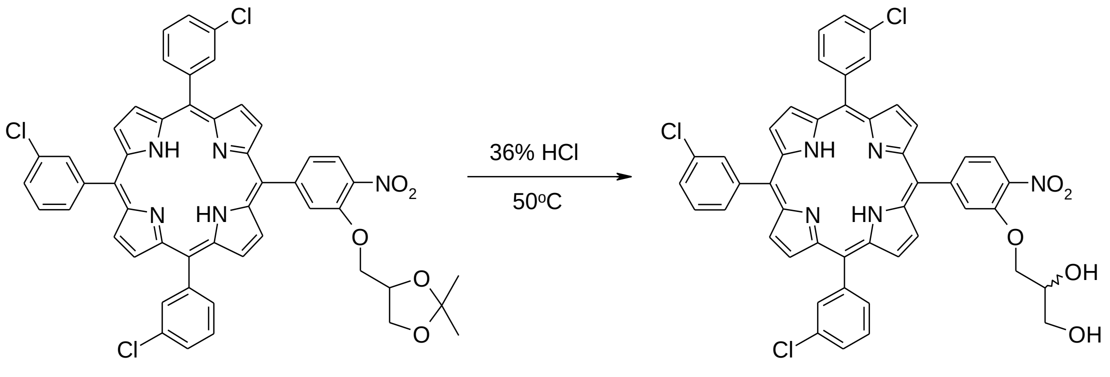

4m

(*rac*)-**6m**

In a 6 mL sealed tube 16.1 mg of porphyrin (*rac*)-**4m** (0.018 mmol) and 1.0 mL of 36% HCl_{aq} were added. The reaction mixture was stirred vigorously at 50°C for 30 min. Mixture was cooled to r.t. and transferred to a separatory funnel with 20 mL of $CHCl_3$ and 20 mL of saturated aqueous solution of sodium carbonate. Organic phase was separated and water phase was extracted 2x10 mL of $CHCl_3$. Combined organic phases were washed with 10 mL of saturated solution of sodium carbonate and then with 10 mL of water. Solvent was evaporated off. Crude product was purified by column chromatography (eluent: $CHCl_3$/MeOH, 95:5) yielding purple solid of porphyrin (*rac*)-**6m** (13.5 mg, 88%).

^{1}H NMR (500 MHz, $CDCl_3$) δ [ppm] = 8.93-8.86 (m, 6H, H^β); 8.84 (d, 2H, J = 4.6 Hz); 8.32 (d, 1H, J = 8.1 Hz, H-Ar); 8.23 (br s, 3H, H-Ar); 8.13-8.09 (m, 3H, H-Ar); 7.98 (br s, 1H, H-Ar); 7.95 (d, 1H, 8.3 Hz, H-Ar); 7.83-7.79 (m, 3H, H-Ar); 7.74-7.67 (m, 3H, H-Ar); 4.42-4.36 (m, 1H, H-gly); 4.35-4.30 (m, 1H, H-gly); 4.19 (s, 1H, H-gly); 3.92-3.81 (m, 2H, H-gly); 3.08 (br s, 1H, OH); 2.24 (br s, 1H, OH); -2.86 (br s, 2H, NH).

^{13}C NMR (125 MHz, $CDCl_3$) δ [ppm] = 150.7; 149.3; 143.6; 143.6; 139.2; 134.5; 133.2; 132.8; 128.5; 128.5; 128.2; 127.4; 124.5; 121.4; 119.6; 119.3; 117.2; 72.0; 71.9; 69.8; 63.4.

MS (APCI) m/z (% rel. int.): 858 (3); 857 (11); 856 (28); 855 (34); 854 (100); 853 (42); 852 (83) (isotope $[M+H]^+$).

HRMS (APCI): $C_{47}H_{33}N_5O_5Cl_3^+$ $[M+H]^+$ (*m/z*): calc. 852.1547; found 852.1556.

UV-VIS ($CHCl_3$) λ_{max} [nm] (log ε): 644.6 (3.50); 589.2 (3.83); 550.6 (3.92); 515.2 (4.35); 419.6 (5.54, Soret band).

*5-[3-(D-glucofuranosyl-3-oxy)-4-nitrophenyl]-10,15,20-tris-(3-chlorophenyl)porphyrin (**6o**)*

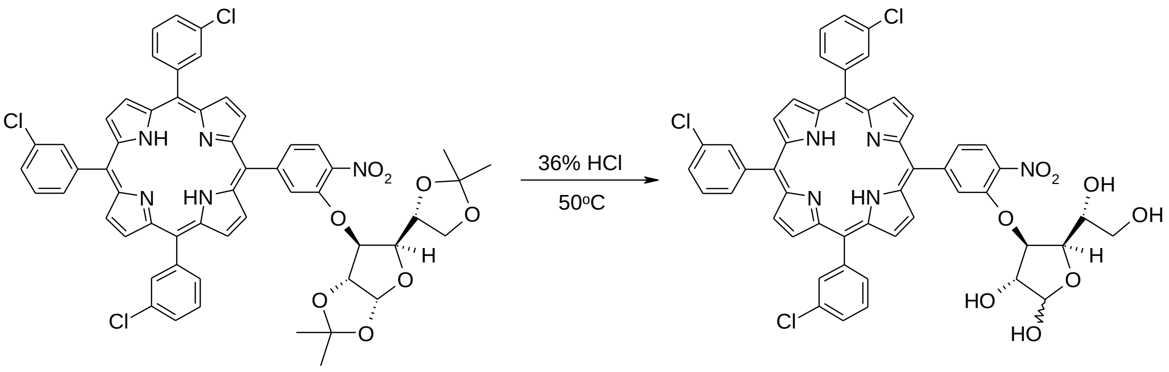

4o **6o**

In a 6 mL sealed tube 12.2 mg of porphyrin **4o** (0.012 mmol) and 1 mL of 36% HCl_{aq} were added. The reaction mixture was stirred vigorously at 50°C for 30 min. Then it was cooled to r.t. and transferred to a separatory funnel with 20 mL of $CHCl_3$ and 20 mL of saturated aqueous solution of sodium carbonate. Organic phase was separated and water phase was extracted 2x10 mL of $CHCl_3$. Combined organic phases were washed with 10 mL of saturated solution of sodium carbonate and then with 10 mL of water. Solvent was evaporated off. Crude product was purified by column chromatography (eluent: $CHCl_3$/MeOH, 95:5) yielding purple solid of porphyrin **6o** (6.9 mg, 61%) as a mixture of two inseparable isomers (A:B, ratio 1:1.2)..

^{1}H NMR (500 MHz, Acetone-d_6) δ [ppm] = 9.53-9.35 (m, 2H, $H^β$); 9.37 (br s, 6H, $H^β$); 9.06-9.01 (m, 1H, H-Ar); 8.78-8.81 (m, 3H, H-Ar); 8.69-8.62 (m, 3H, H-Ar); 8.62-8.58 (m, 1H, H-Ar); 8.38-8.24 (m, 7H, H-Ar); 6.31 (s, 0.45H, H-glu isom A); 5.99 (s, 0.45H, H-glu isom A); 5.59 (s, 0.55H-glu isom B); 5.36 (m, 0.55H, H-glu isom B); 5.30-5.15 (m, 1.65H, H-glu isom B); 4.99-4.94 (m, 0.45H, H-glu isom A); 4.75-4.69 (m, 0.45H, H-glu isom A); 4.38-4.29 (m, 1H, H-glu isom A + isom B); 4.28-4.18 (m, 1.65H, H-glu isom B); 4.17-4.02 (m, 2.55H, H-glu isom A + isom B); 3.98-3.94 (m, 0.45H, H-glu isom A); 3.76-3.71 (m, 0.45H, H-glu isom A); -2.40 (s, 2H, 2xNH).

MS (APCI) m/z (% rel. int.): 944 (23); 943 (35); 942 (100); 941 (32); 940 (78) (isotope $[M+H]^+$).

HRMS (APCI): $C_{50}H_{37}N_5O_8Cl_3^+$ $[M+H]^+$ (*m/z*): calc. 940.1708; found 940.1717.

UV-VIS ($CHCl_3$) λ_{max} [nm] (log ε): 644.4 (3.40); 589.6 (3.78); 550.0 (3.84); 515.2 (4.30); 419.8 (5.55, Soret band).

NMR and UV-VIS spectra of new compounds

^{1}H NMR of **2g**

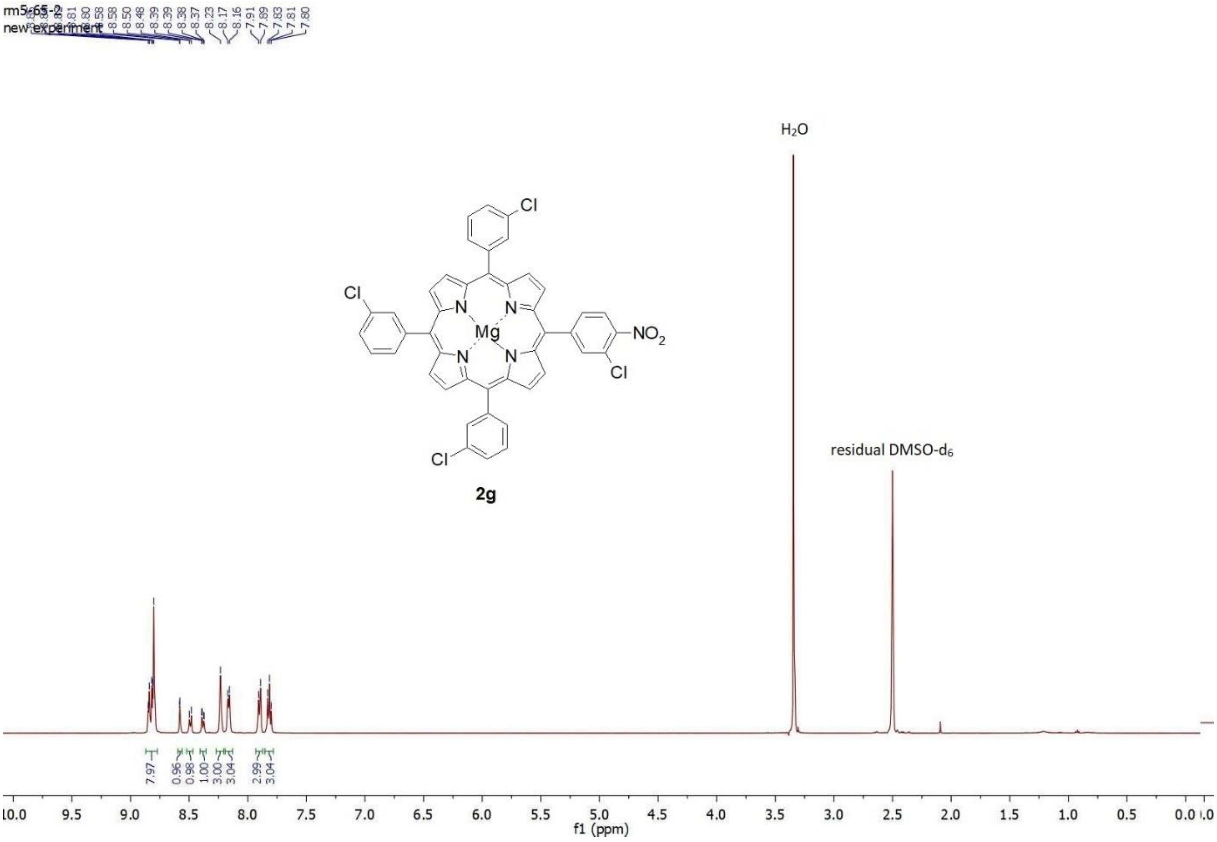

UV-VIS spectrum of **2g**

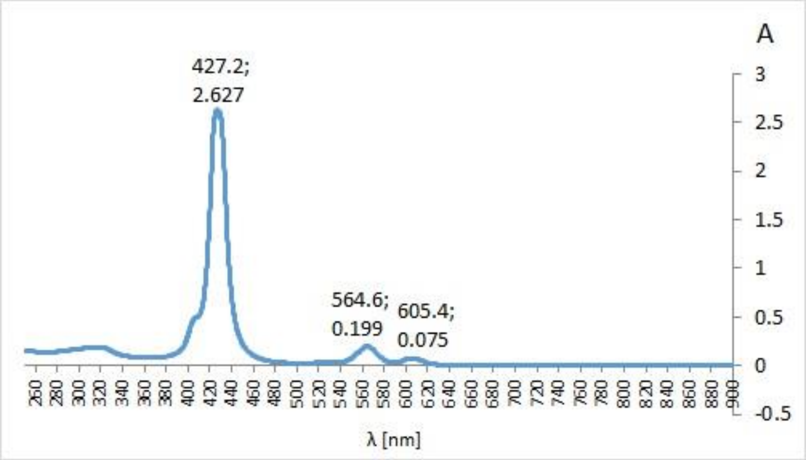

^{1}H NMR spectrum of **2i**

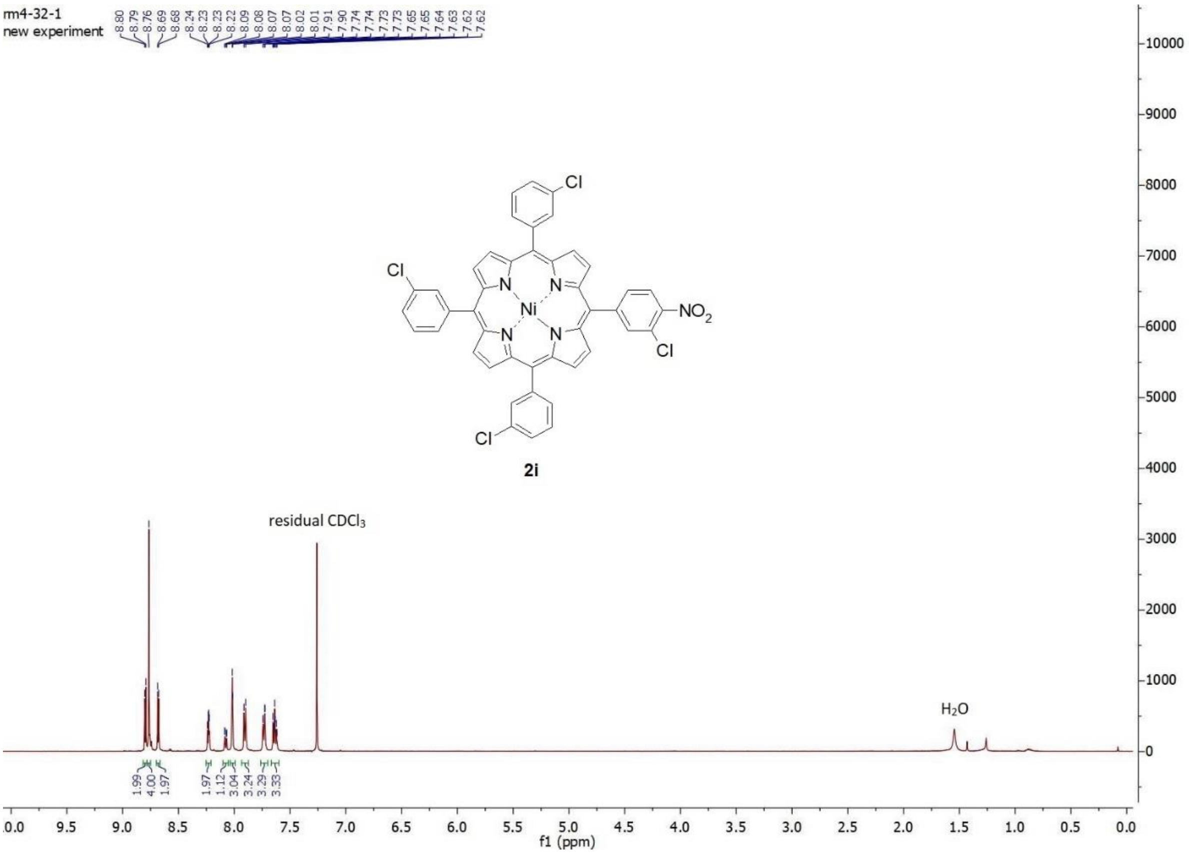

UV-VIS spectrum of **2i**

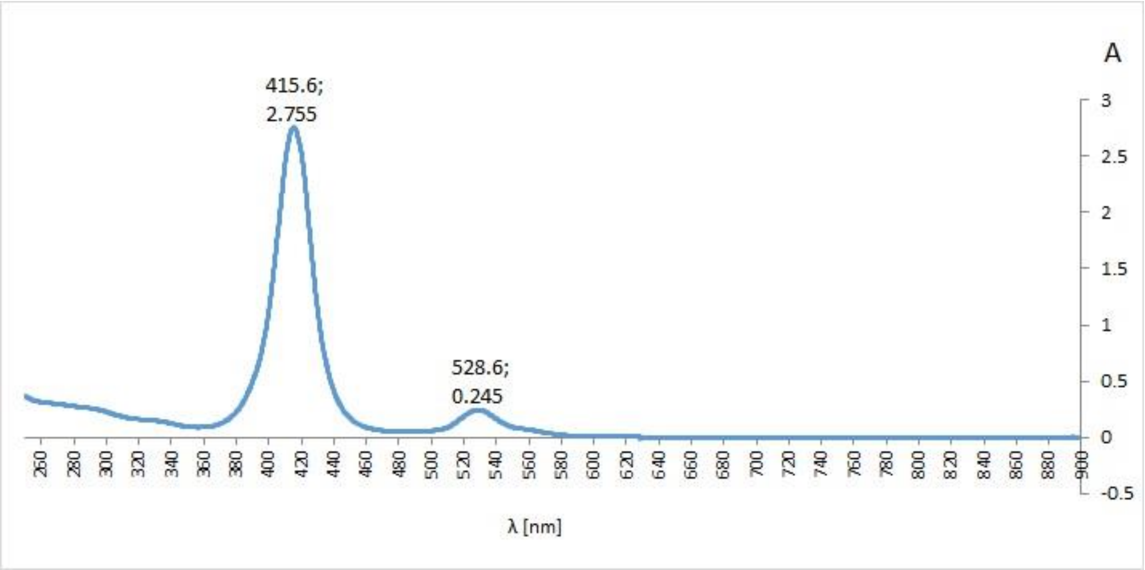

^{1}H NMR of **2n**

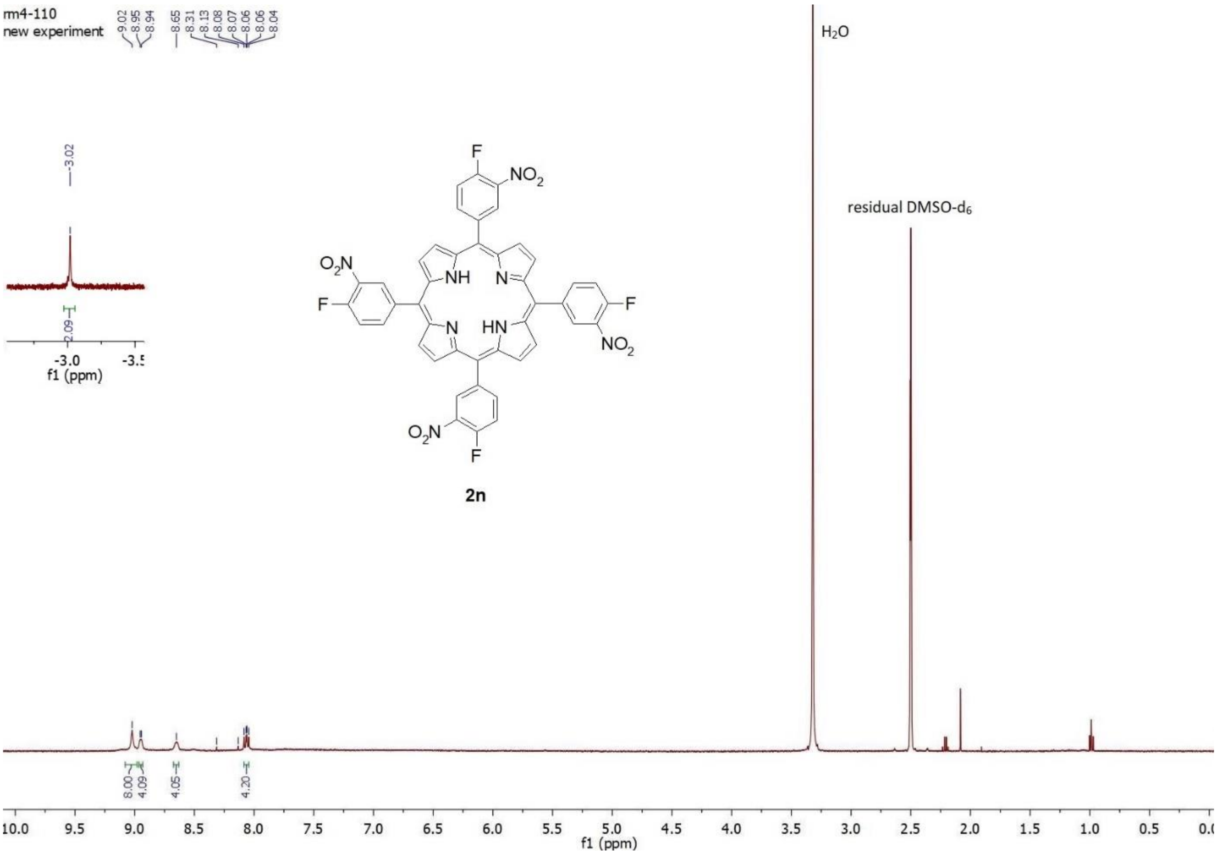

UV-VIS spectrum of **2n**

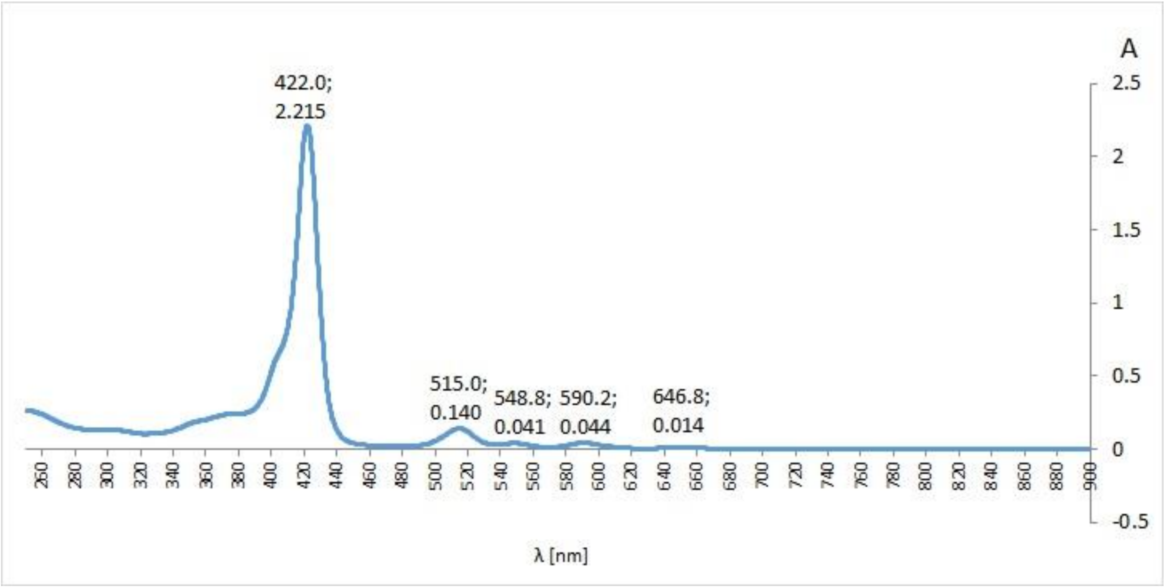

^{1}HNMR of **4a**

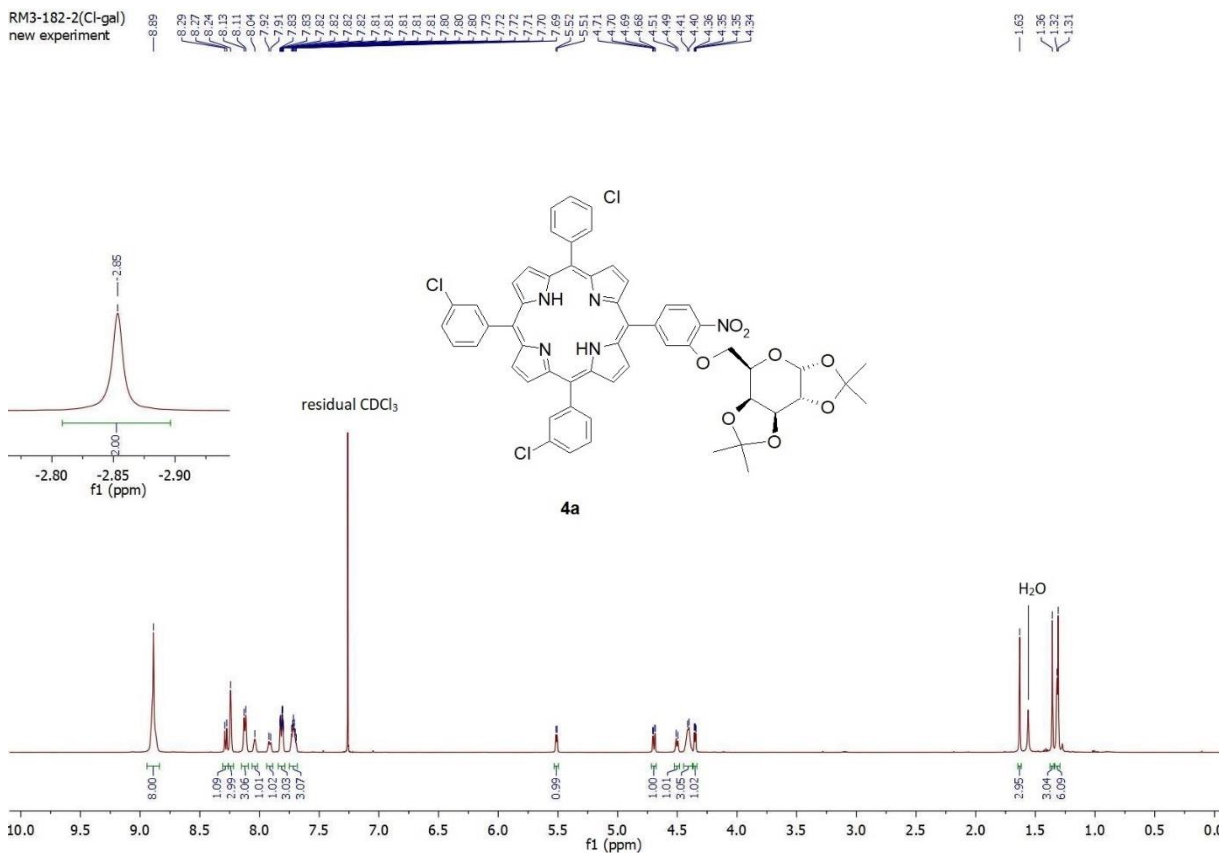

^{13}C NMR of **4a**

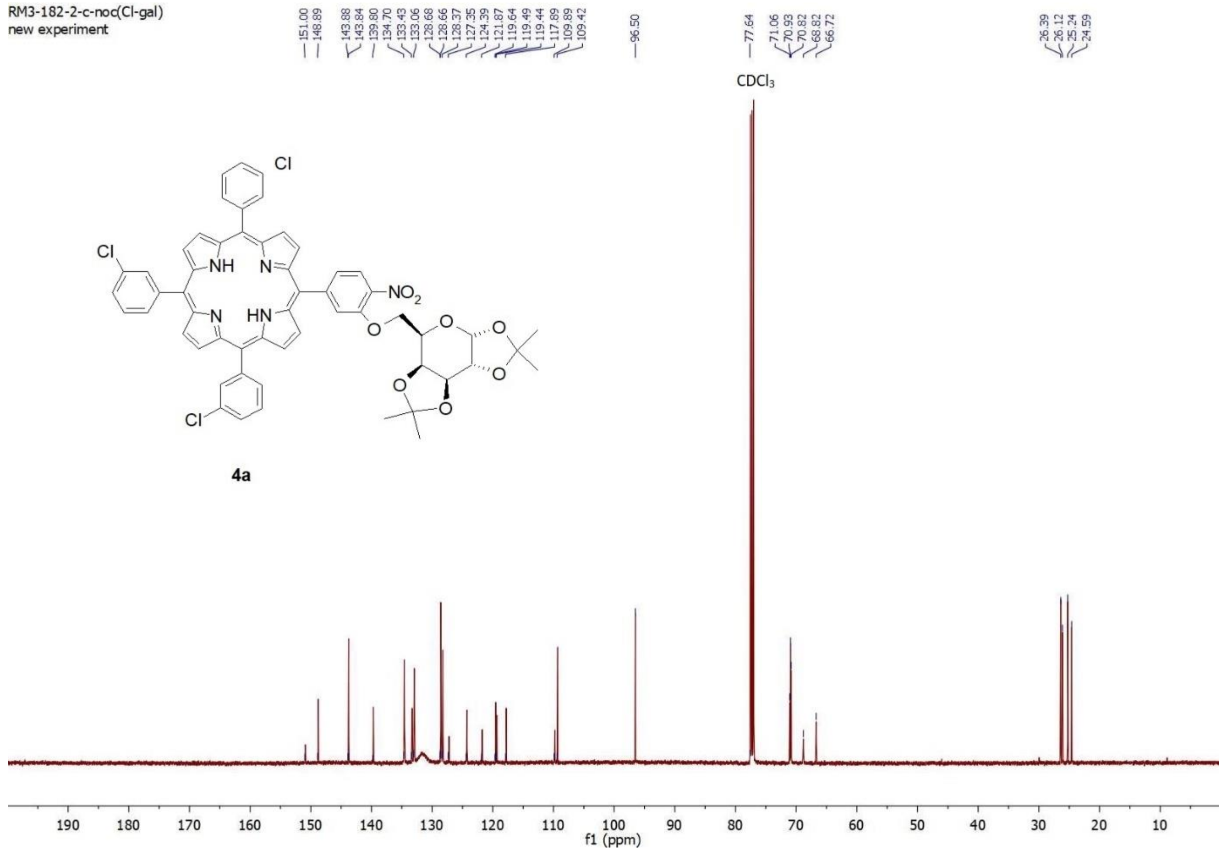

UV-VIS spectrum of **4a**

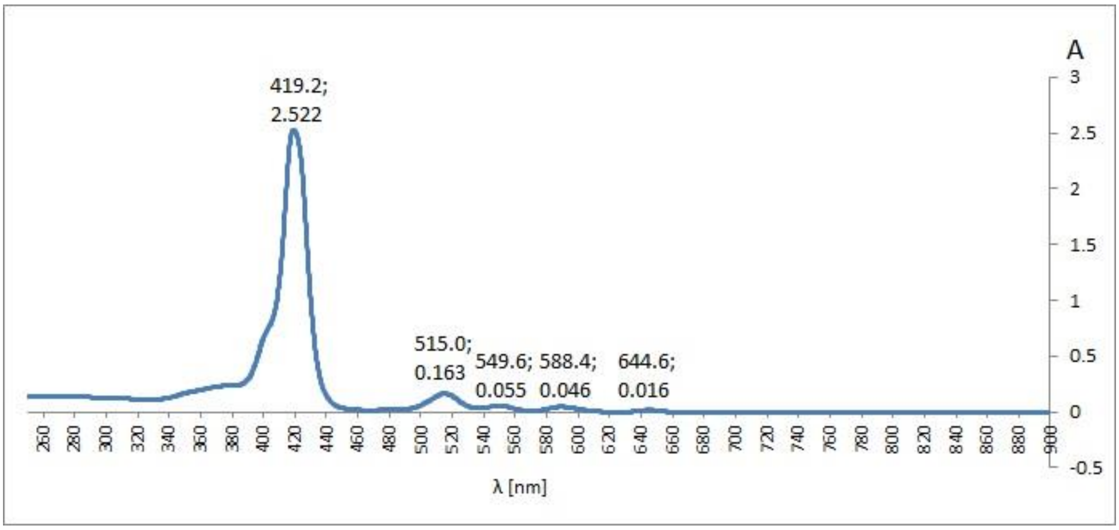

^{1}H NMR of **4b**

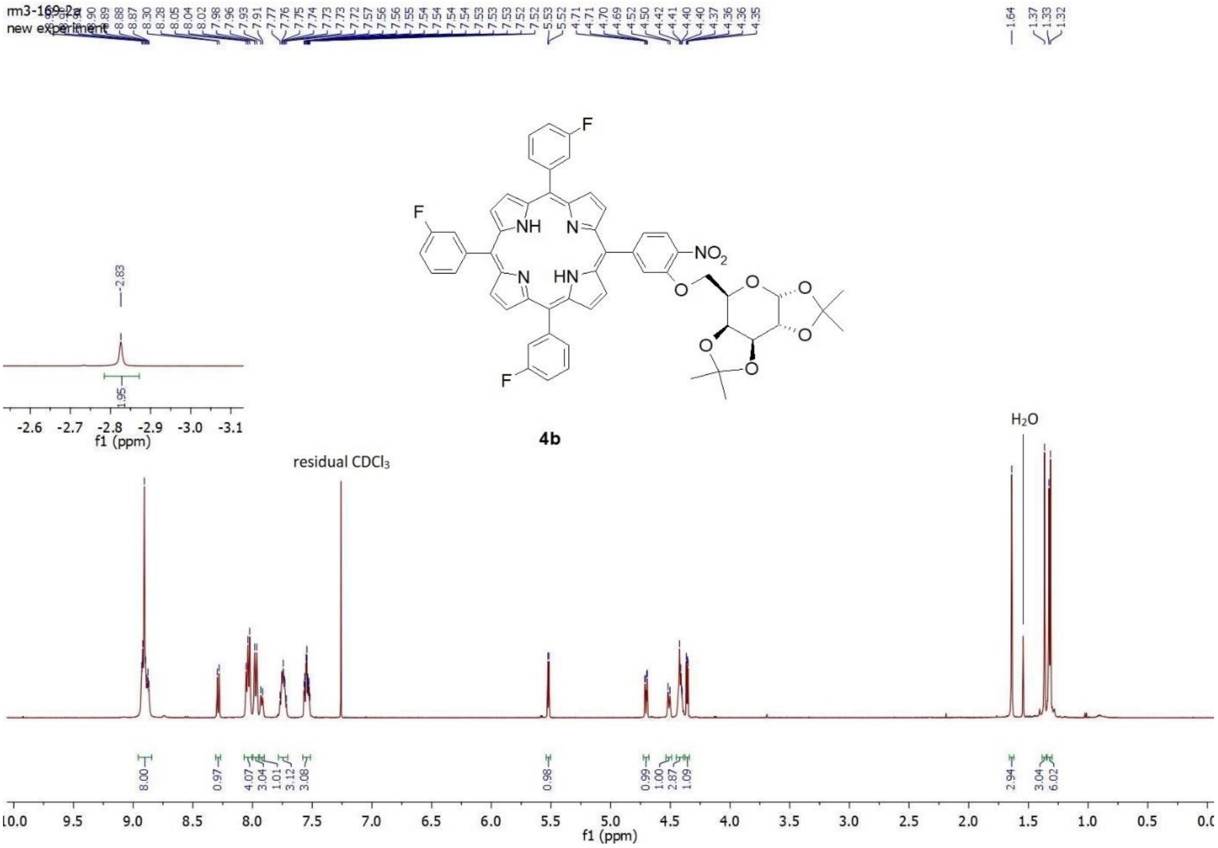

^{19}F of **4b**

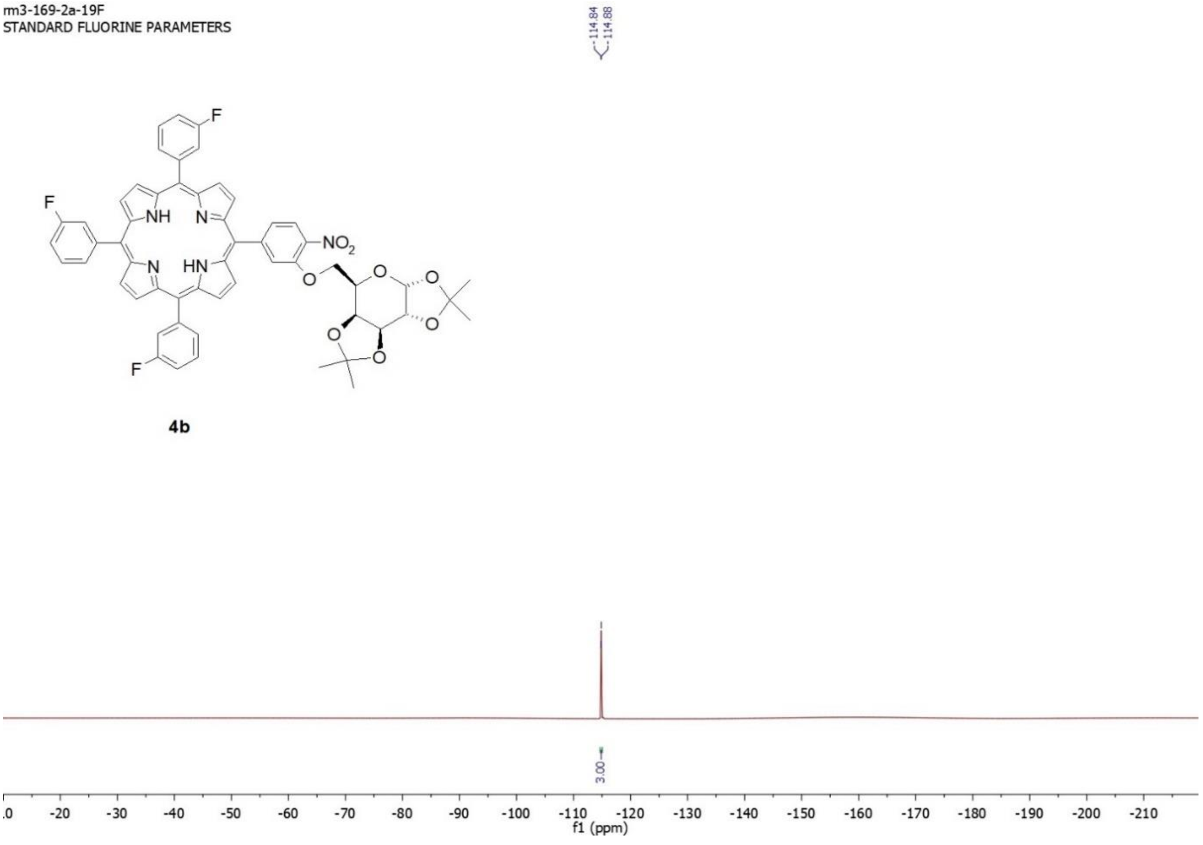

^{13}C of **4b**

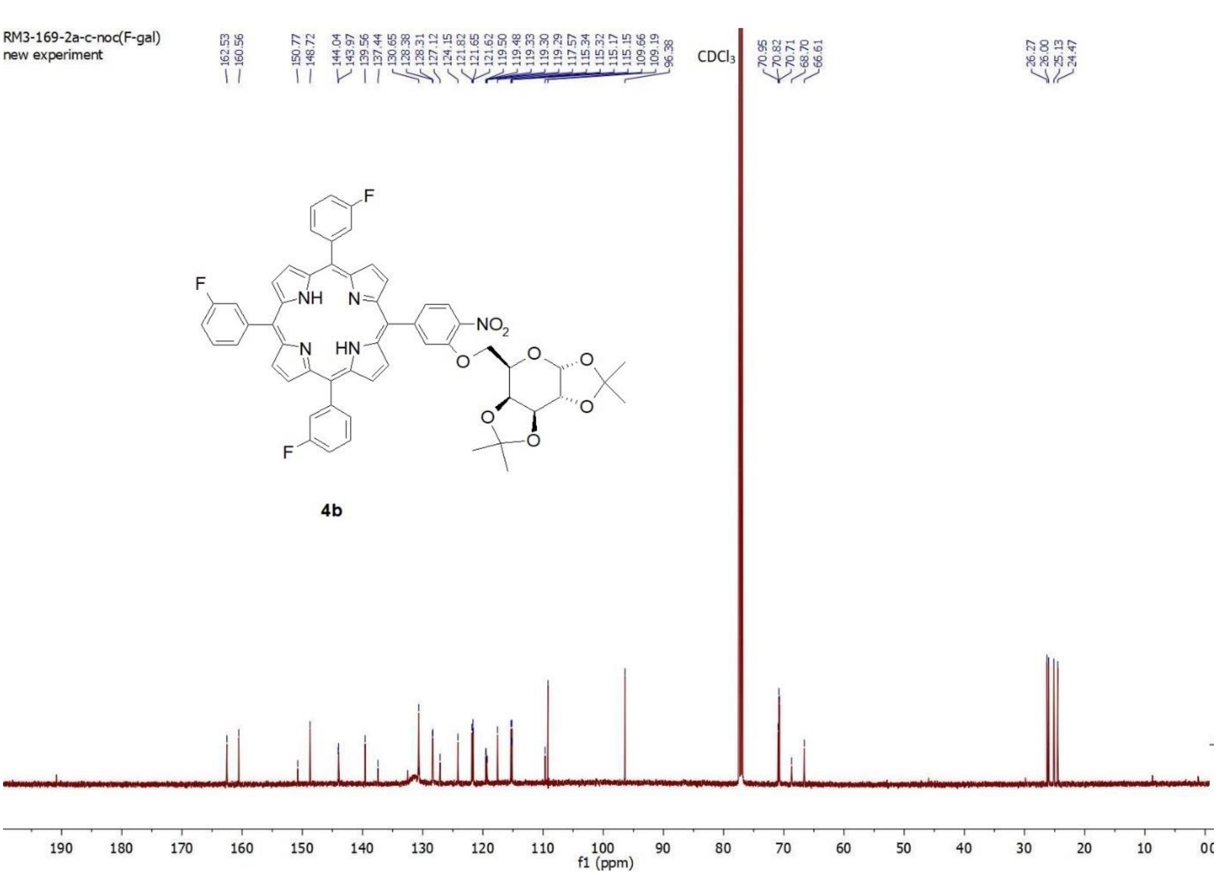

UV-VIS spectrum of **4b**

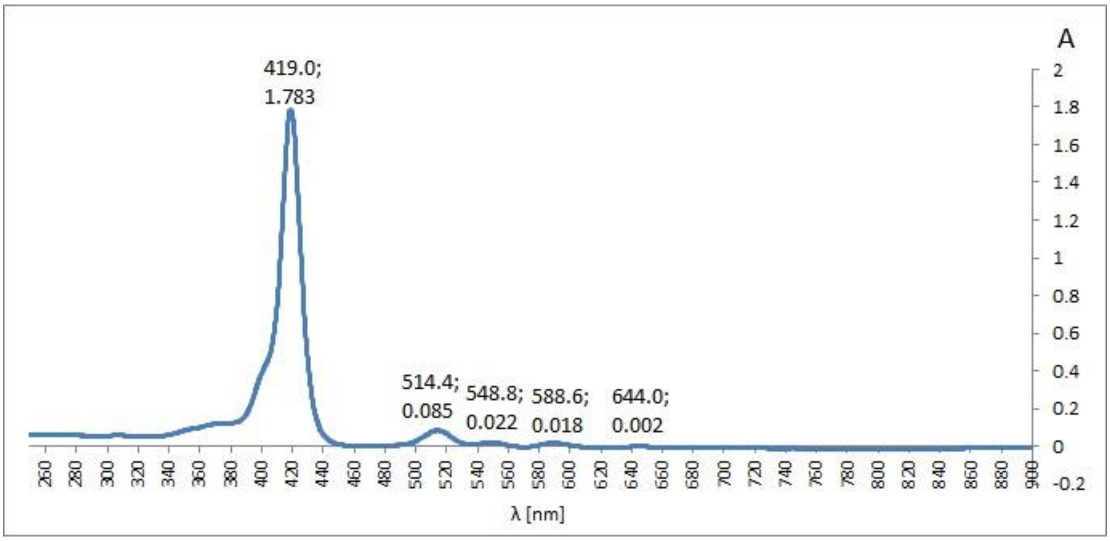

^{1}H NMR of **4c**

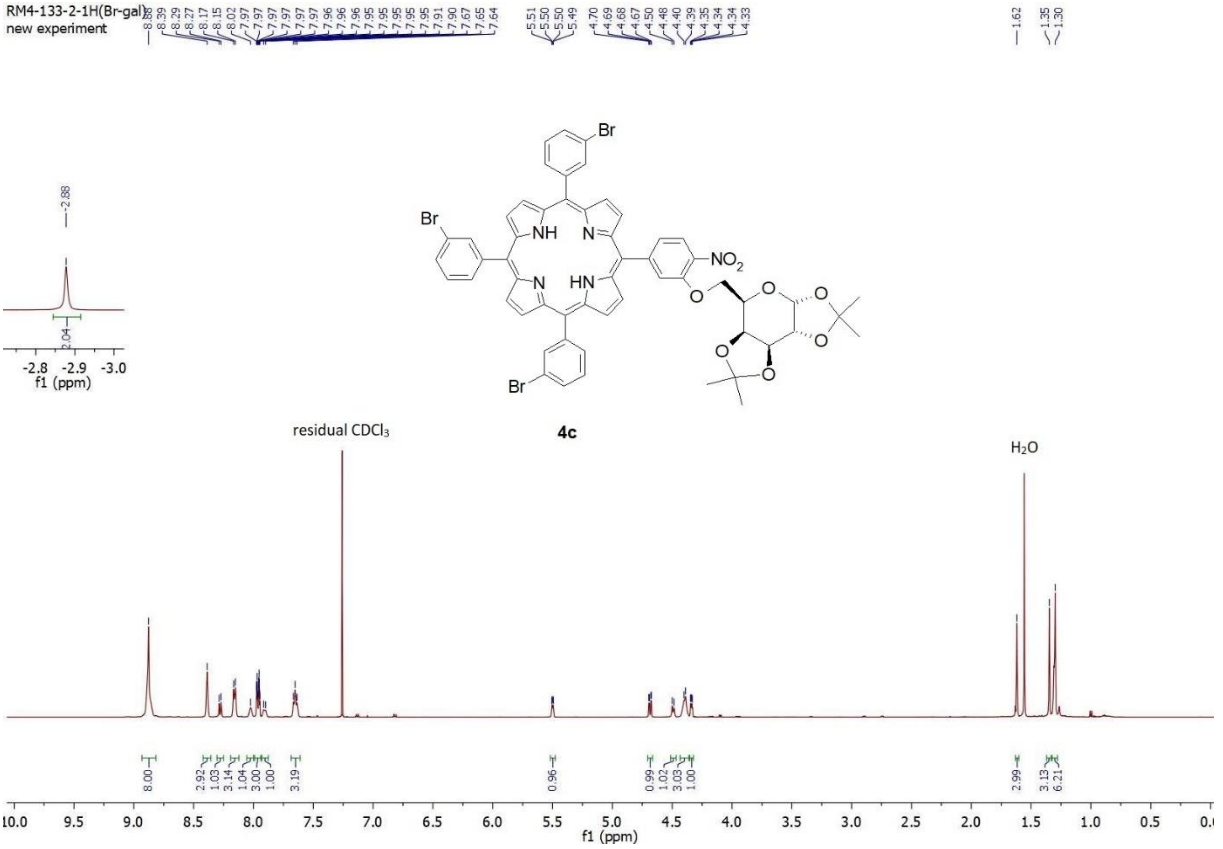

^{13}C NMR of **4c**

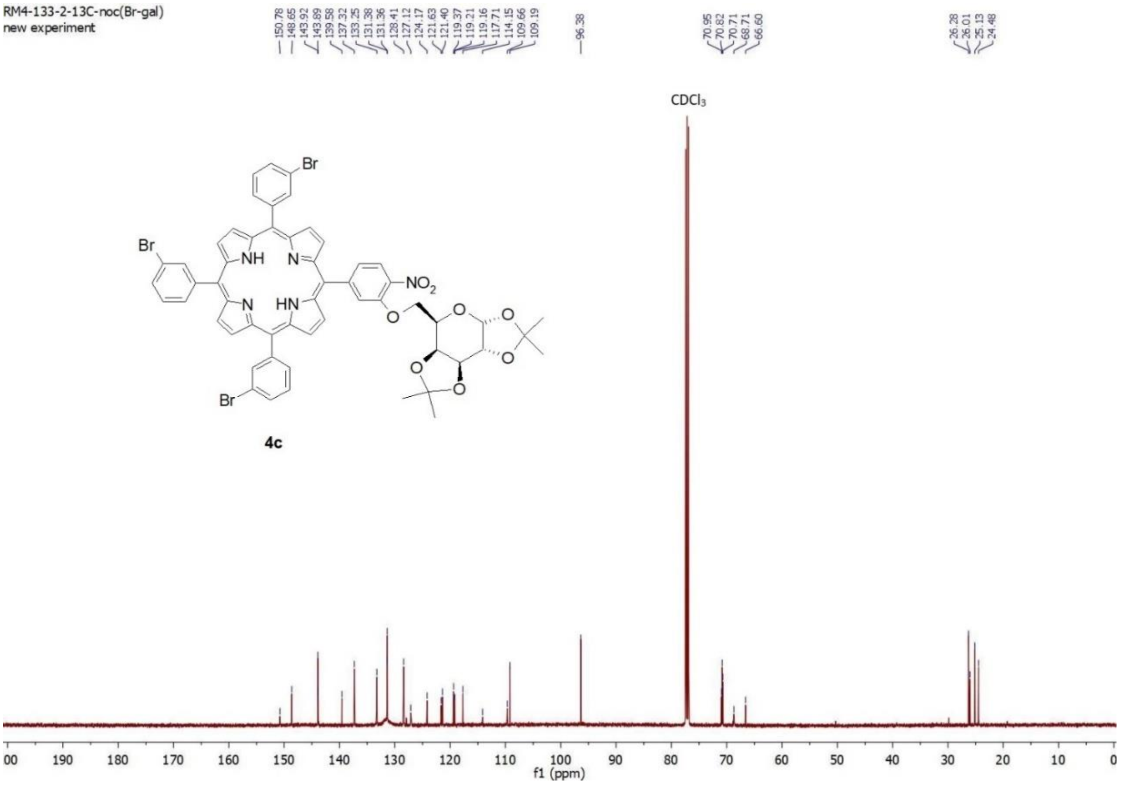

UV-VIS spectrum of **4c**

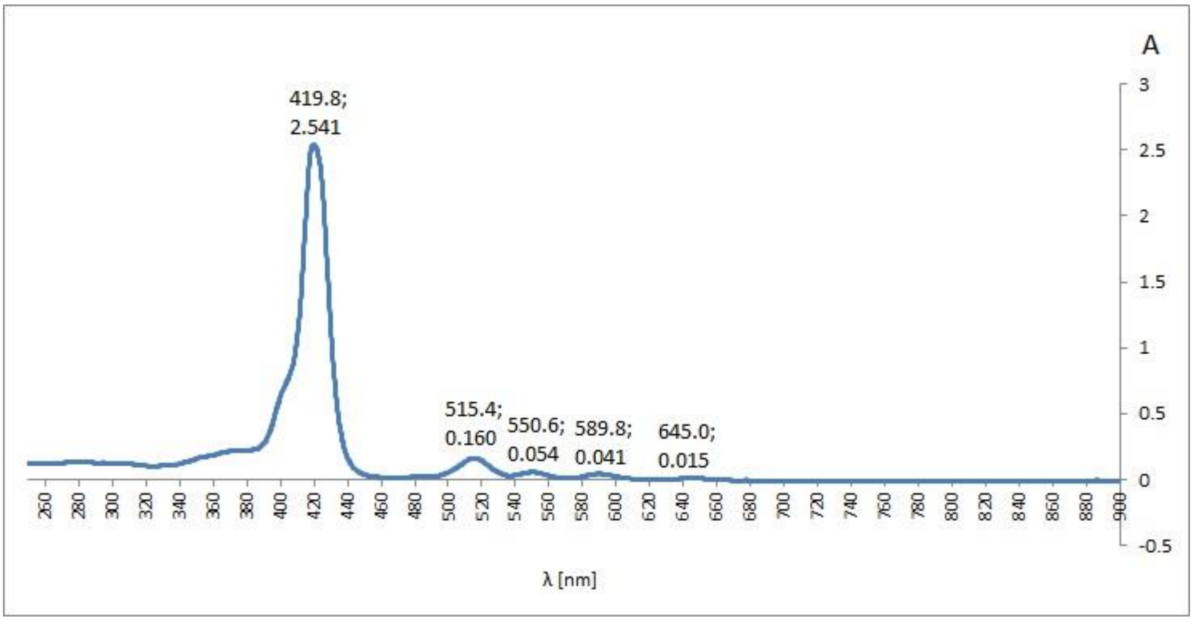

^1H NMR of **4d**

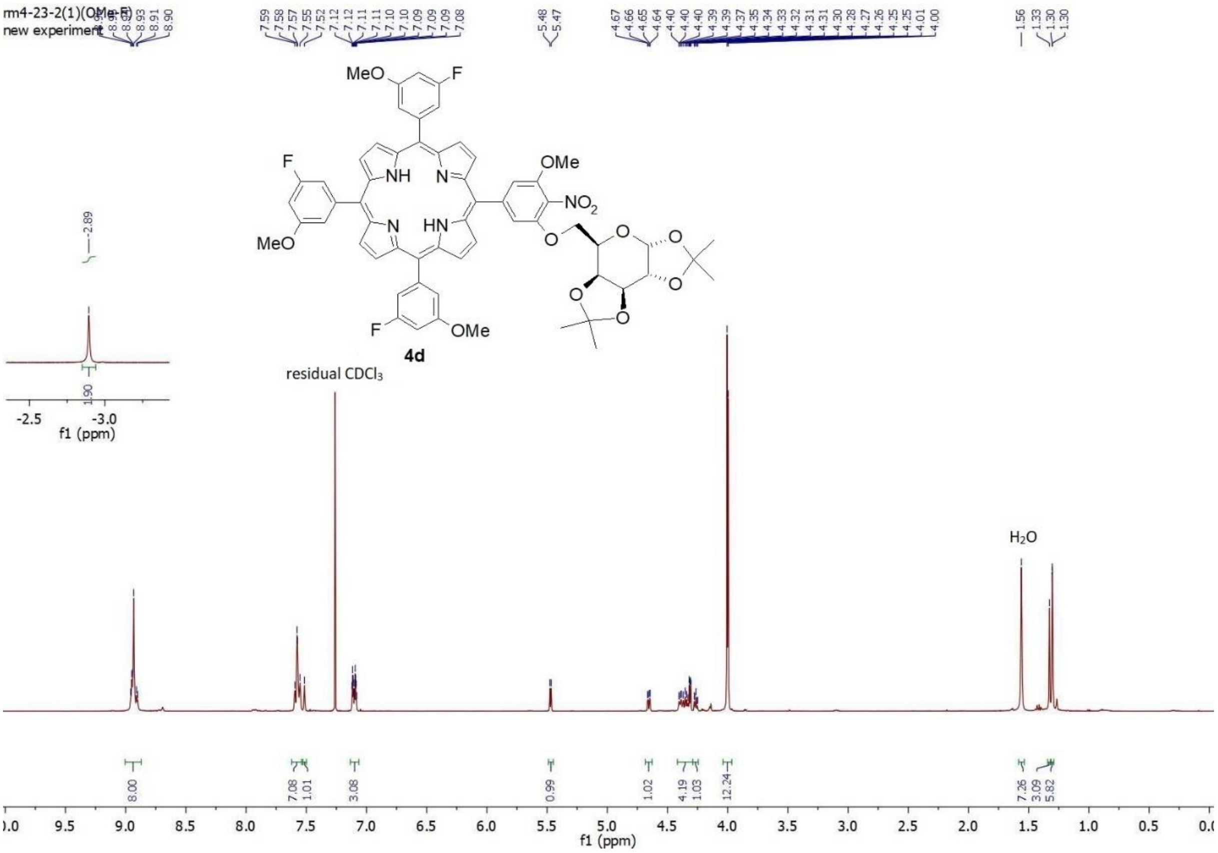

^{13}C NMR of **4d**

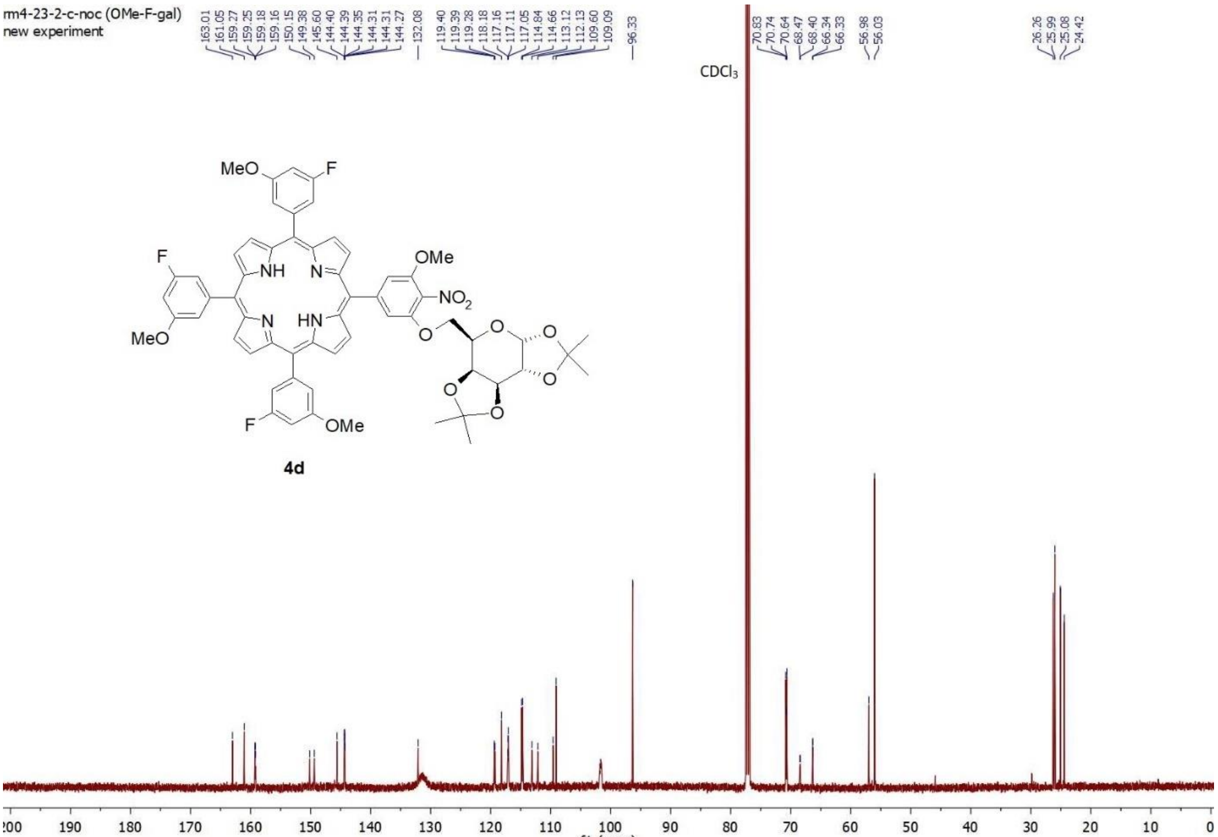

UV-VIS spectrum of **4d**

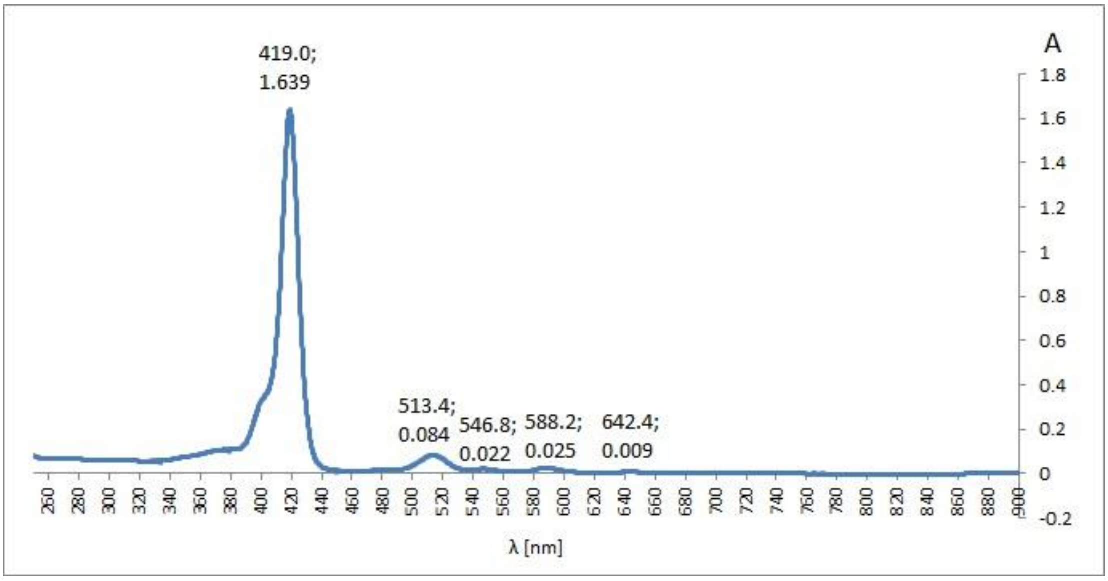

^{1}H NMR of **4e**

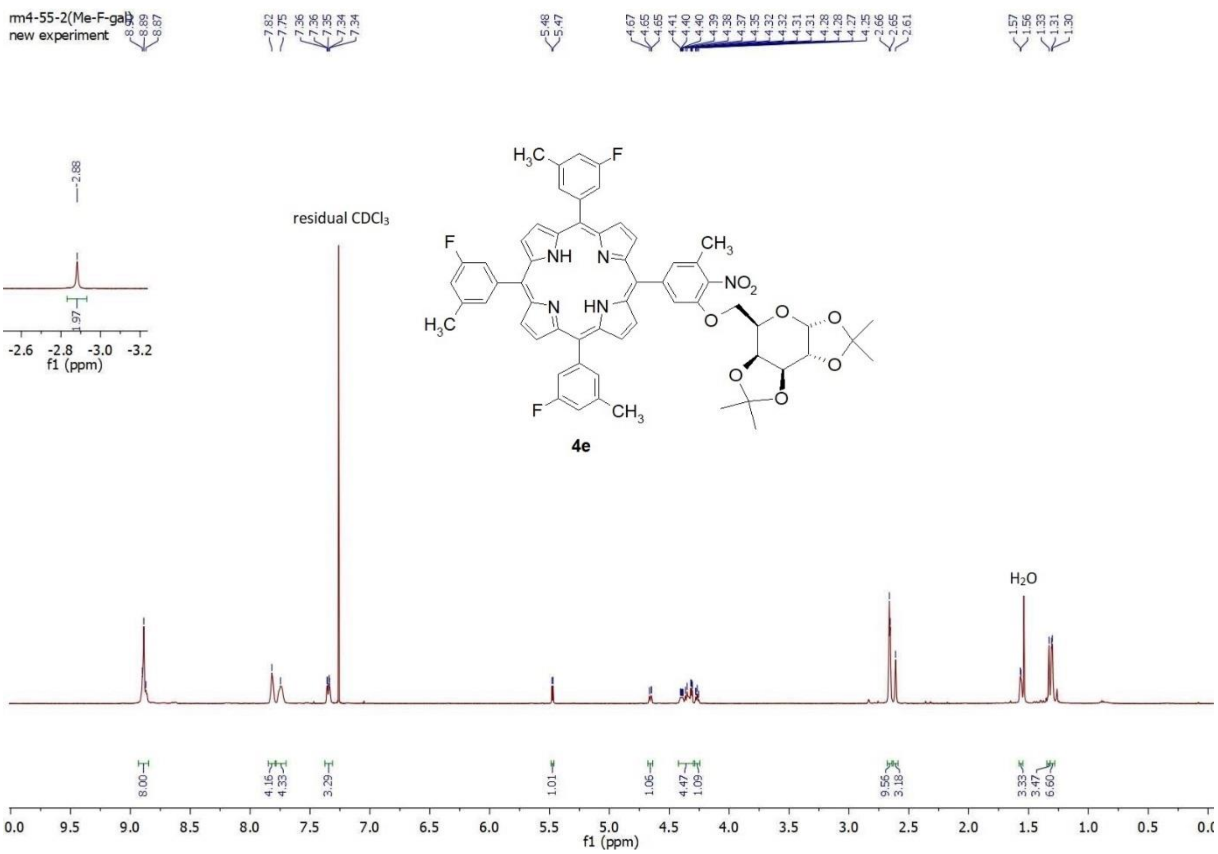

^{13}C NMR of **4e**

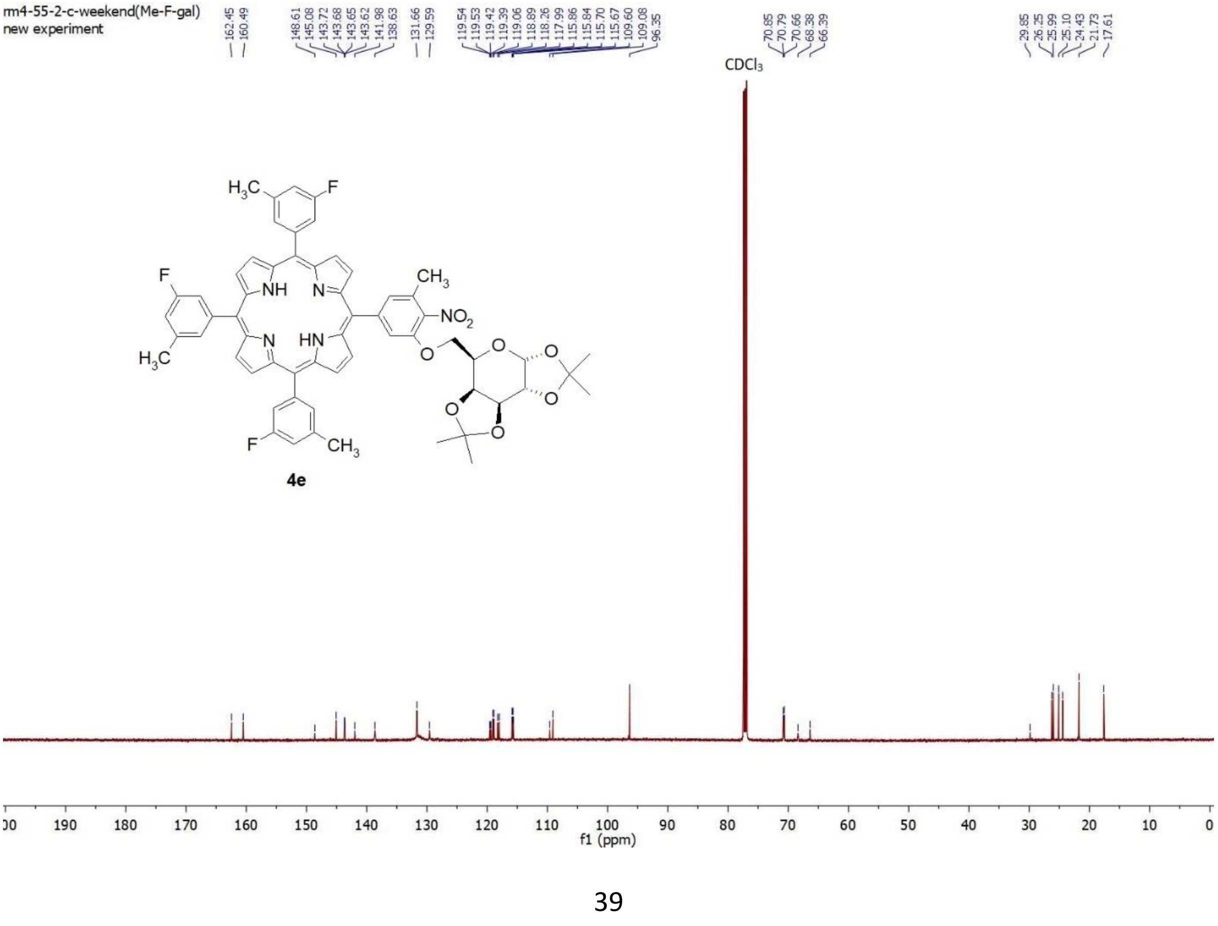

^{19}F NMR of **4e**

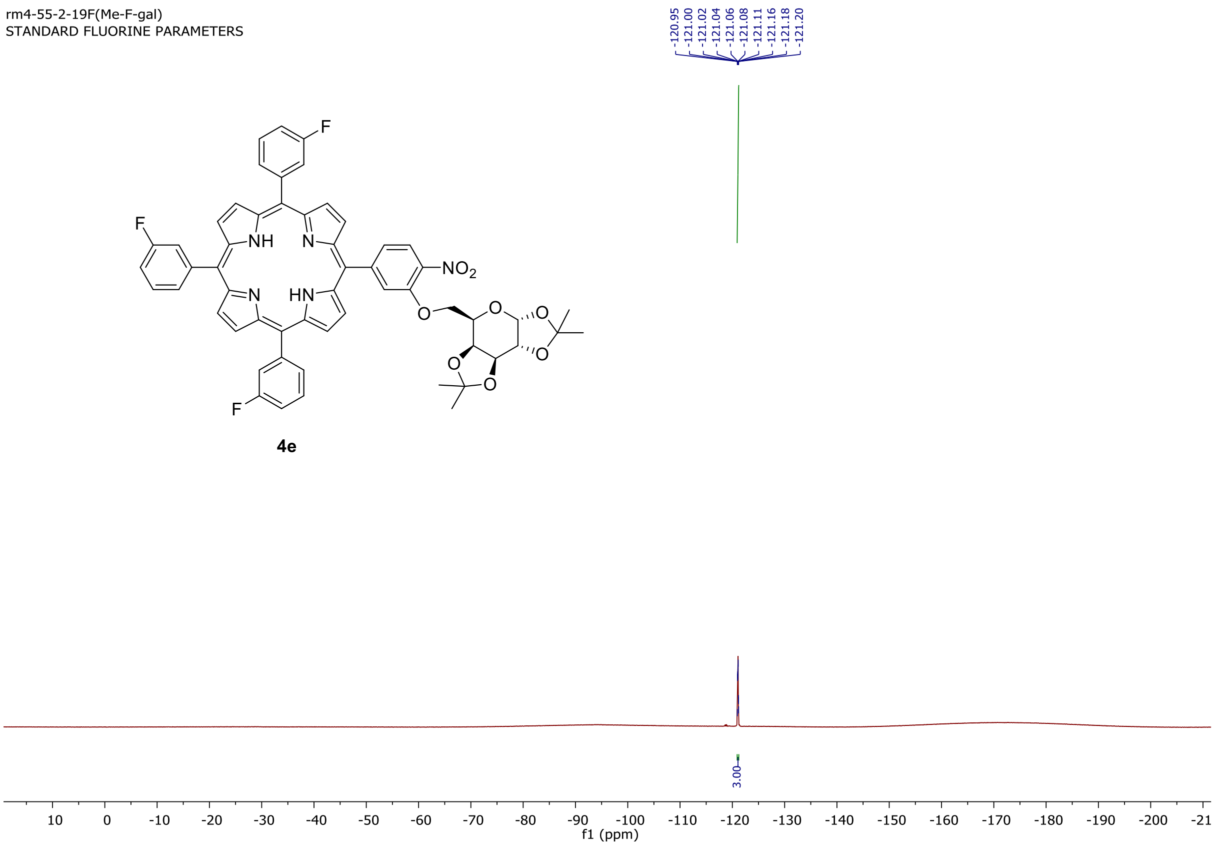

UV-VIS spectrum of **4e**

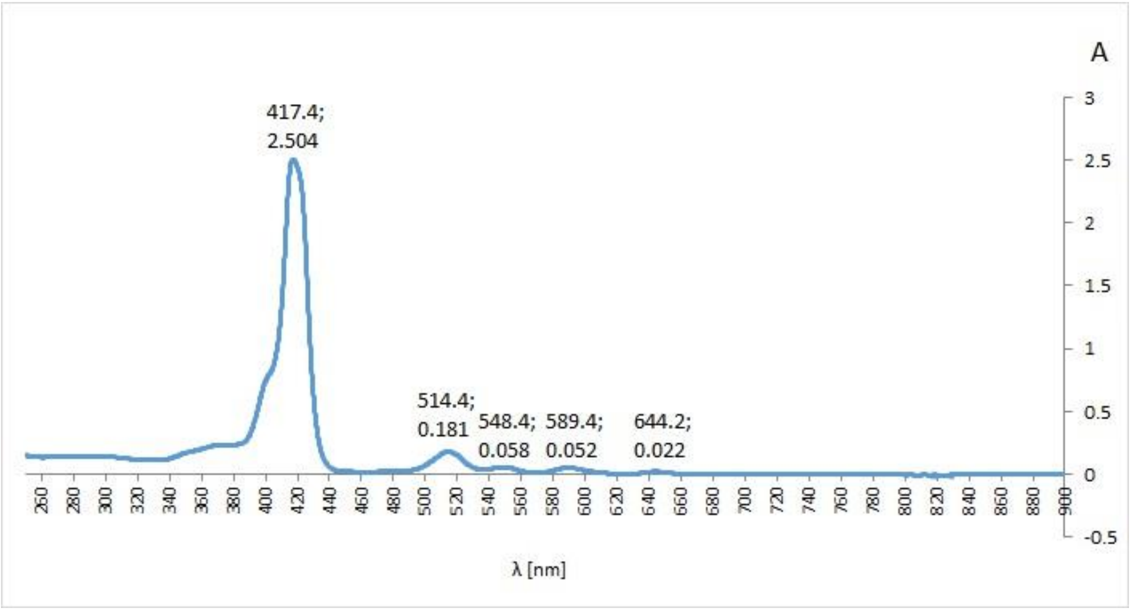

^{1}H NMR of **4f**

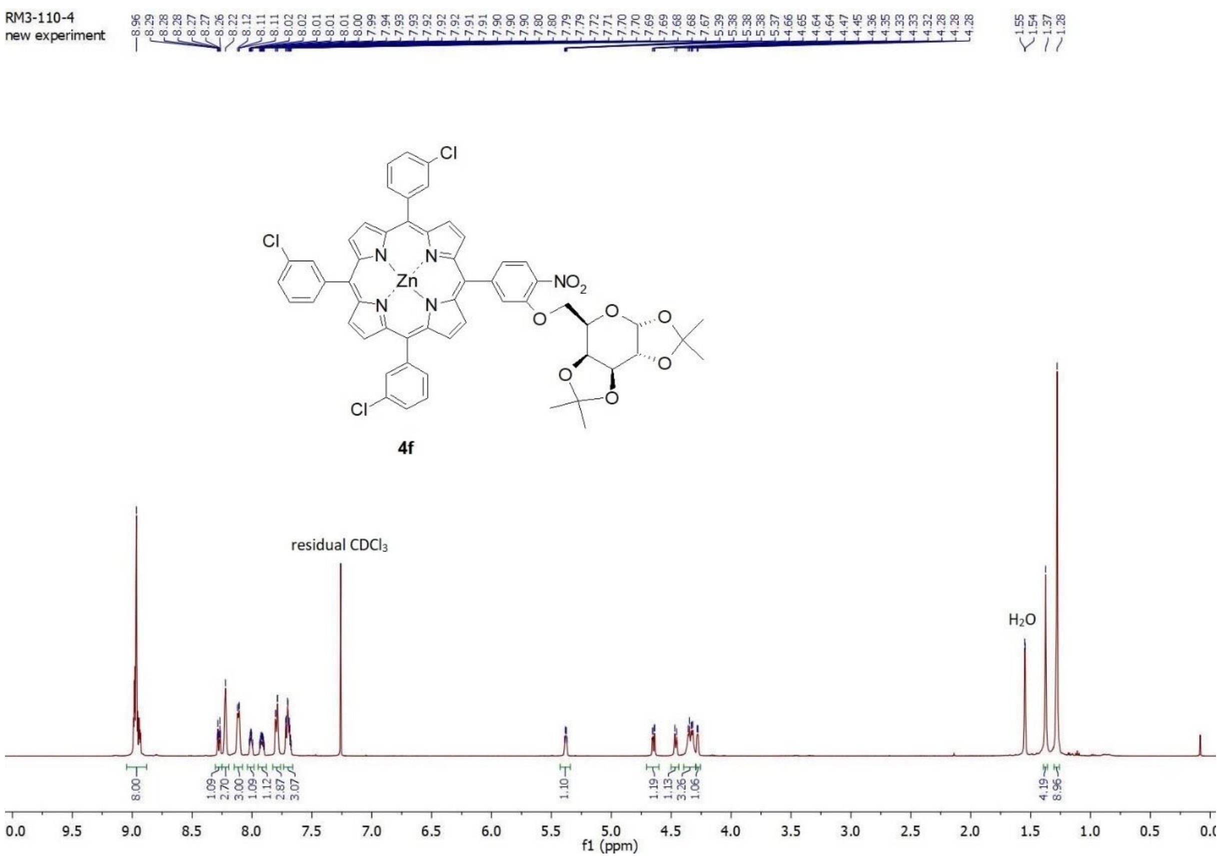

^{13}C NMR of **4f**

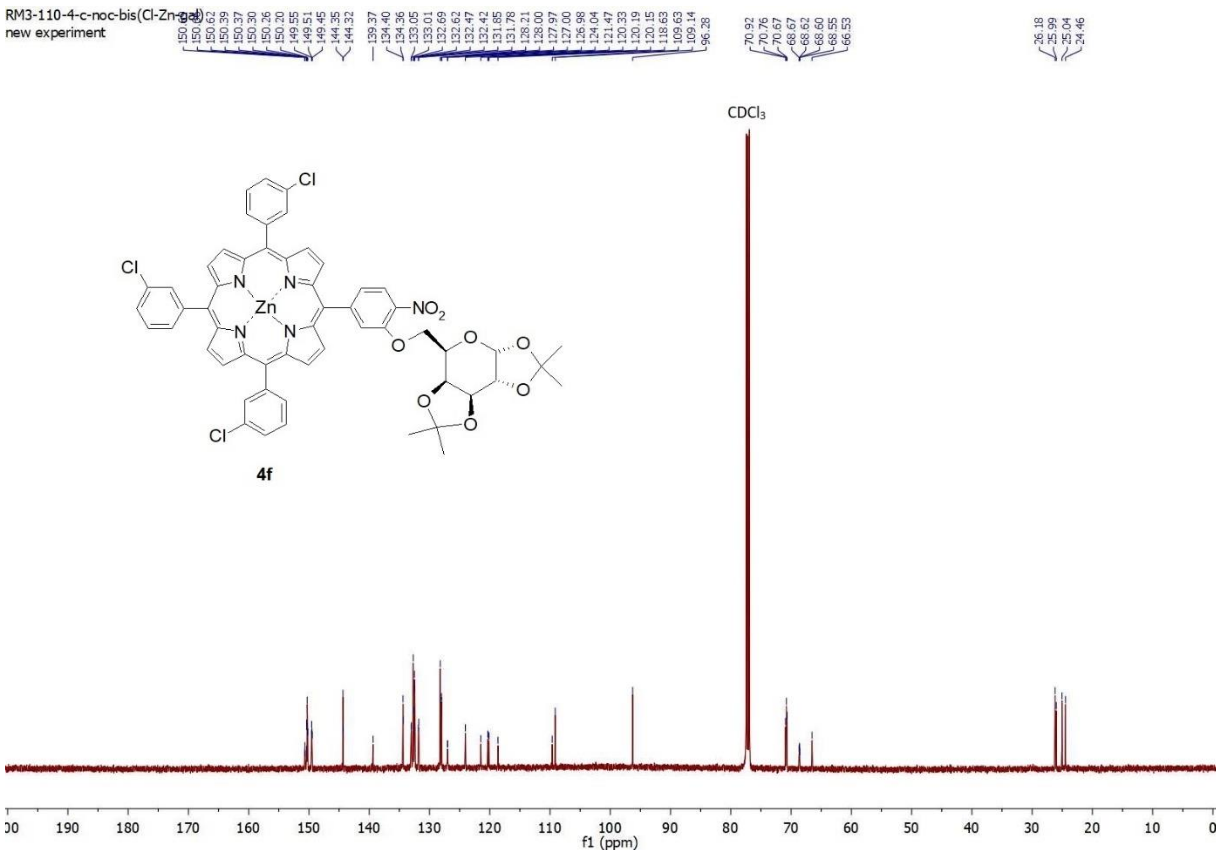

UV-VIS spectrum of **4f**

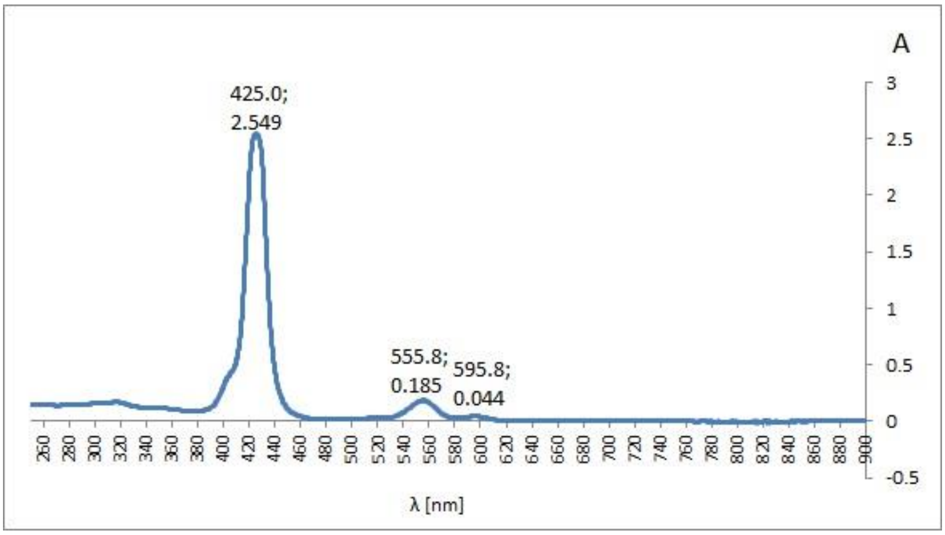

^{1}H NMR of **4g**

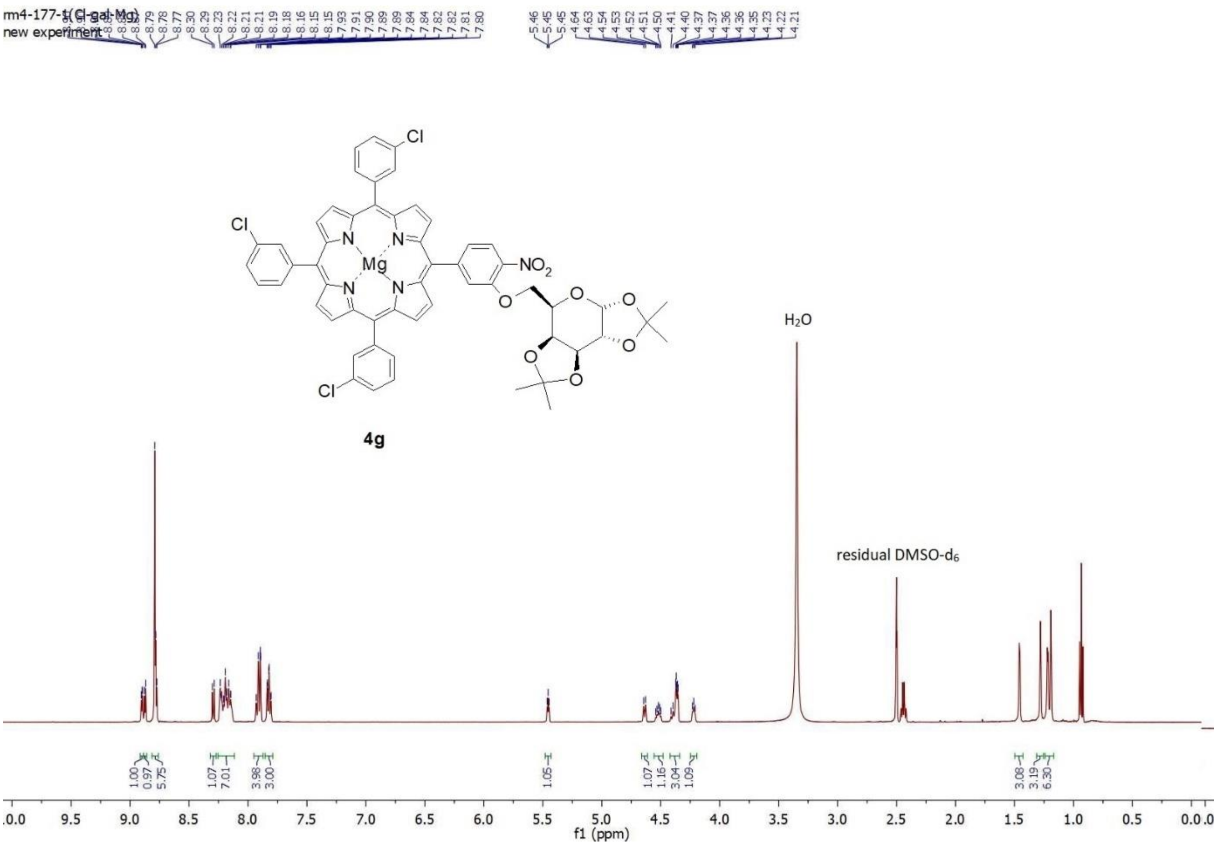

^{13}C NMR of **4g**

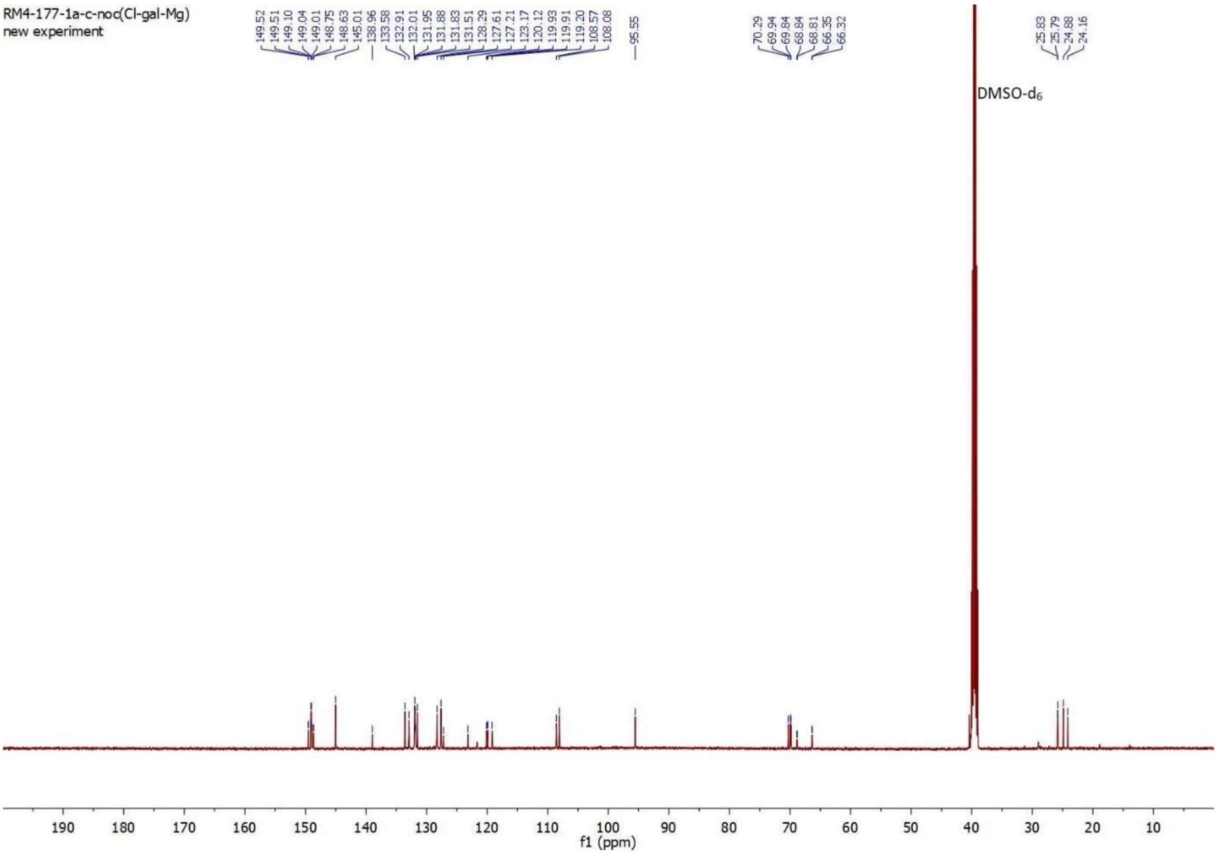

UV-VIS spectrum of **4g**

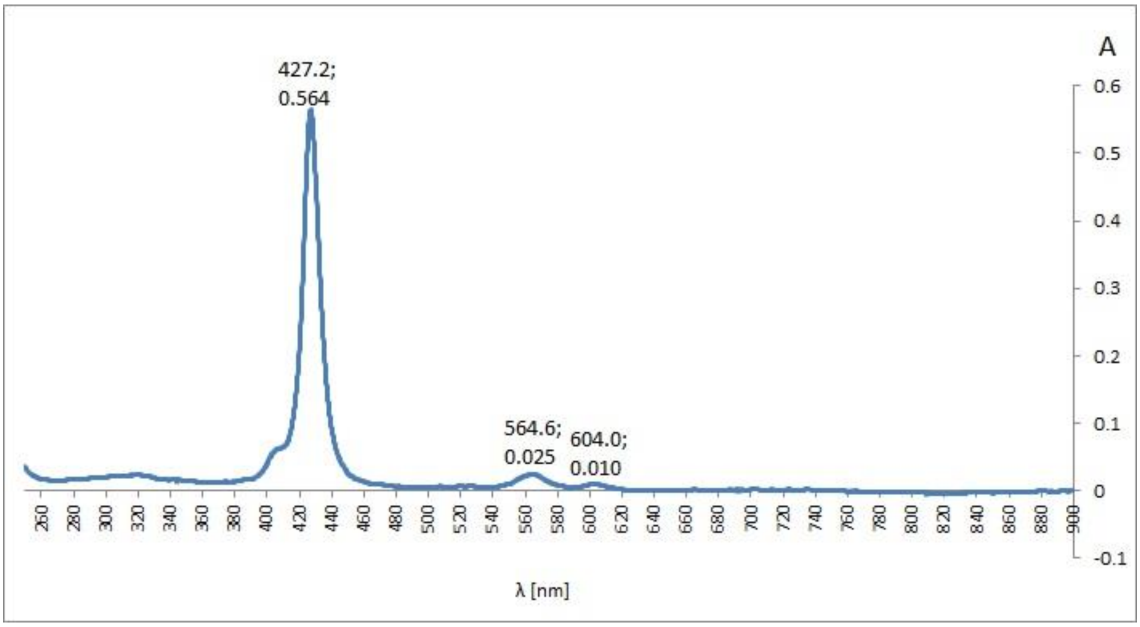

UV-VIS spectrum of **4h**

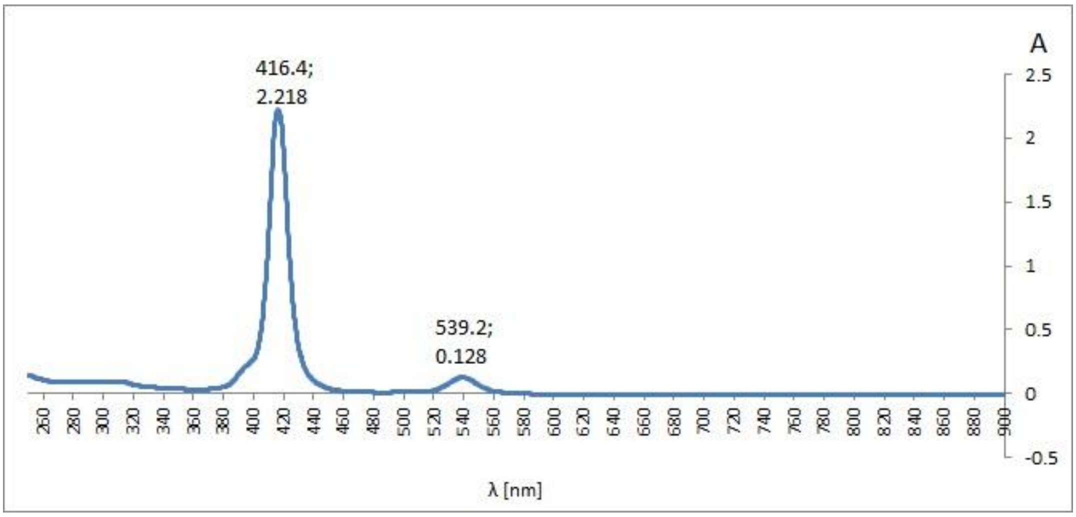

^{1}H NMR spectrum of **4j**

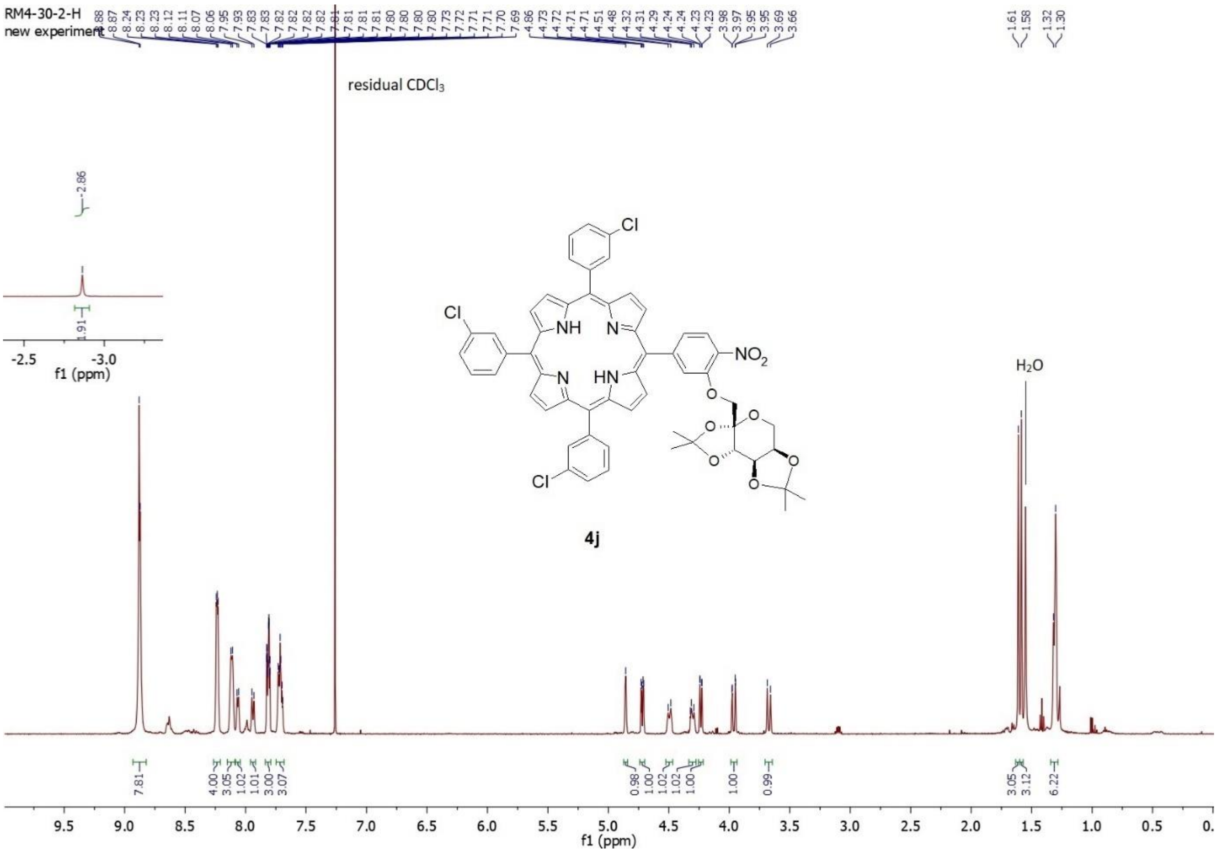

^{13}C NMR of **4j**

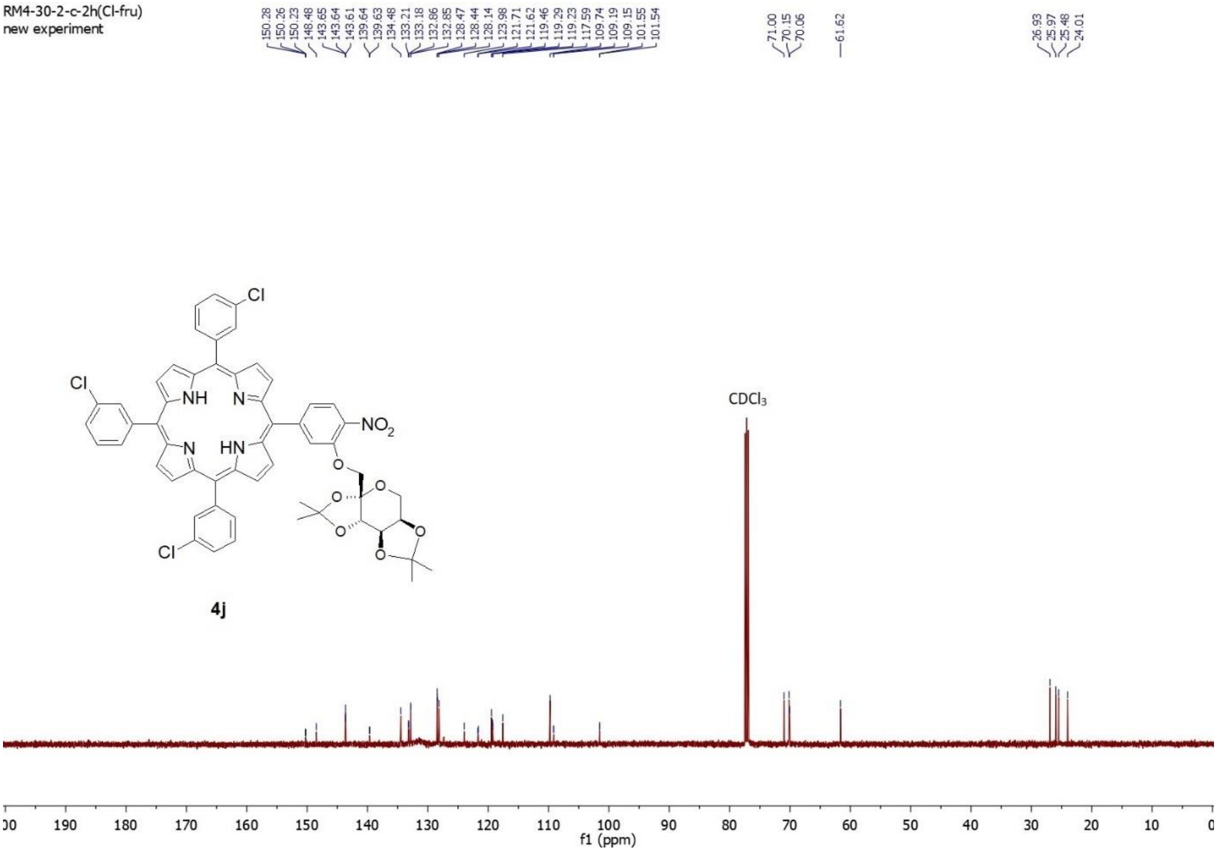

UV-VIS spectrum of **4j**

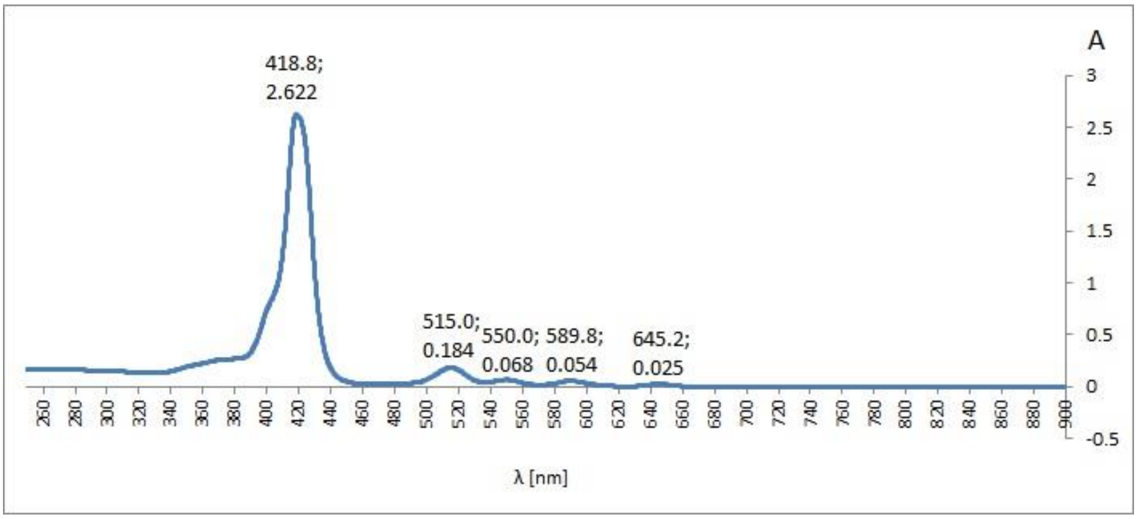

^{1}H NMR of **4k**

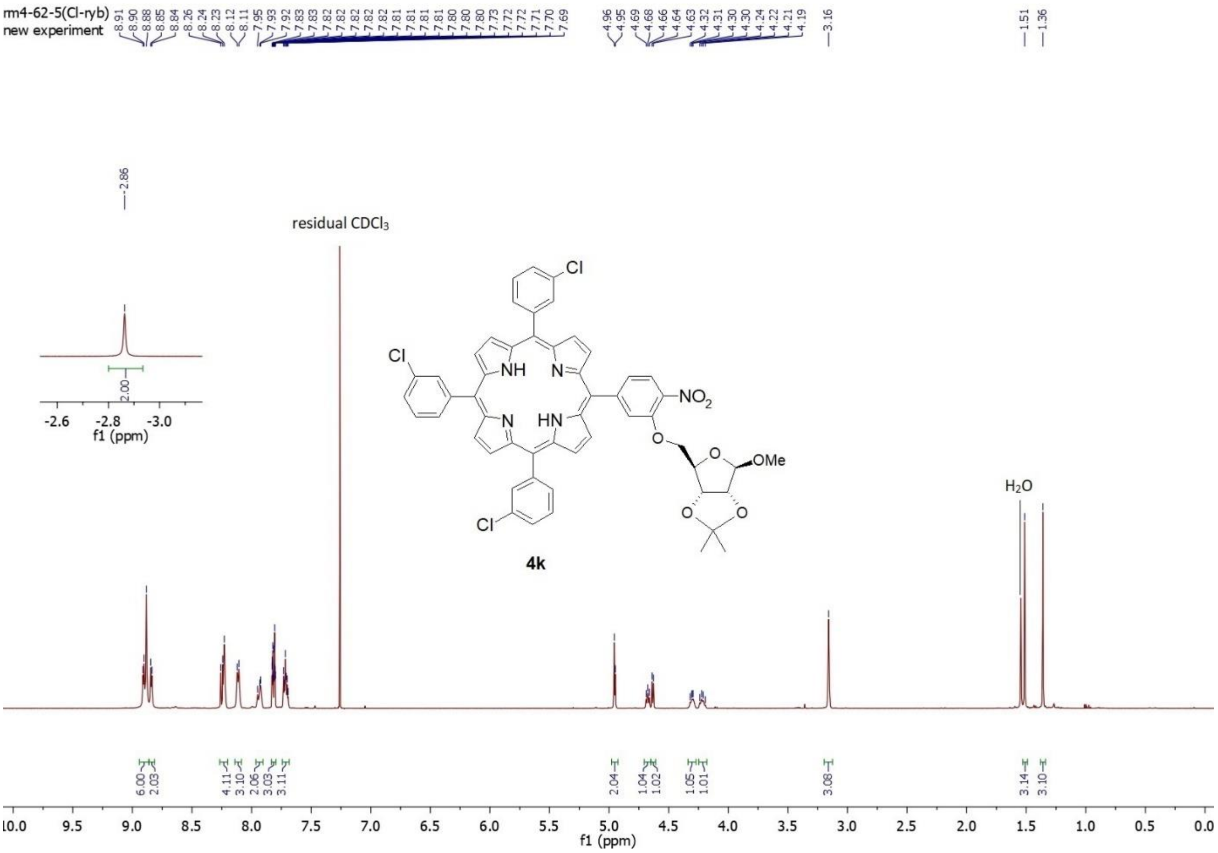

^{13}C NMR of **4k**

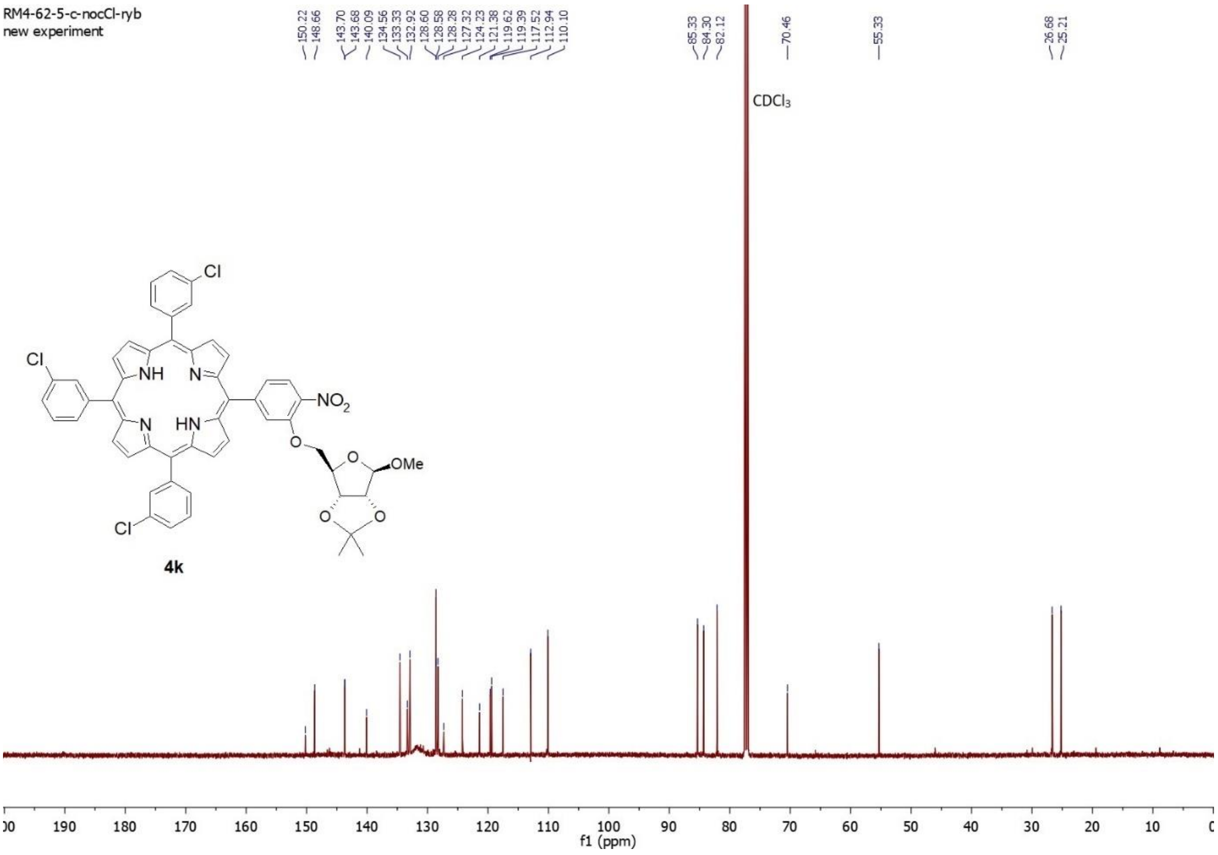

UV-VIS spectrum of **4k**

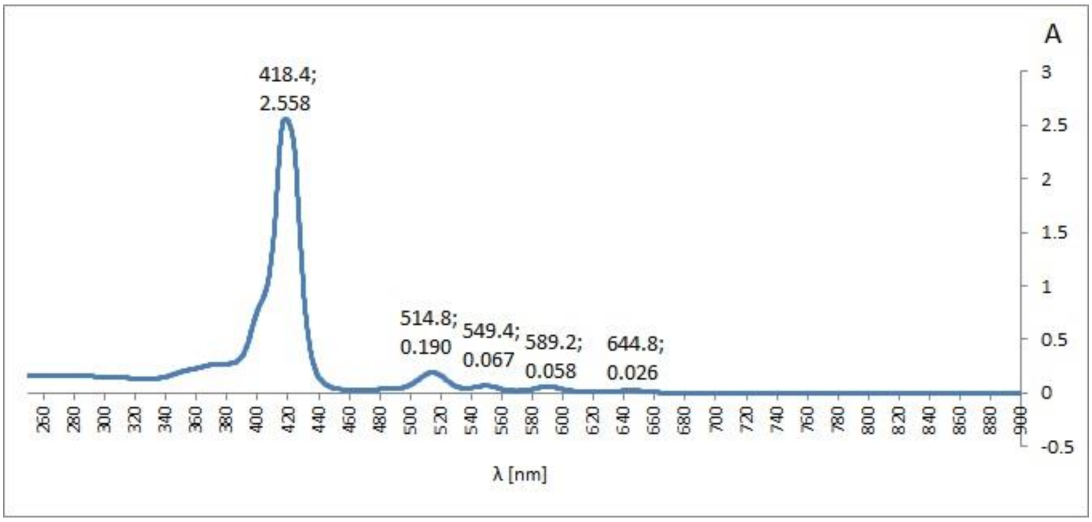

^{1}H NMR of **4l**

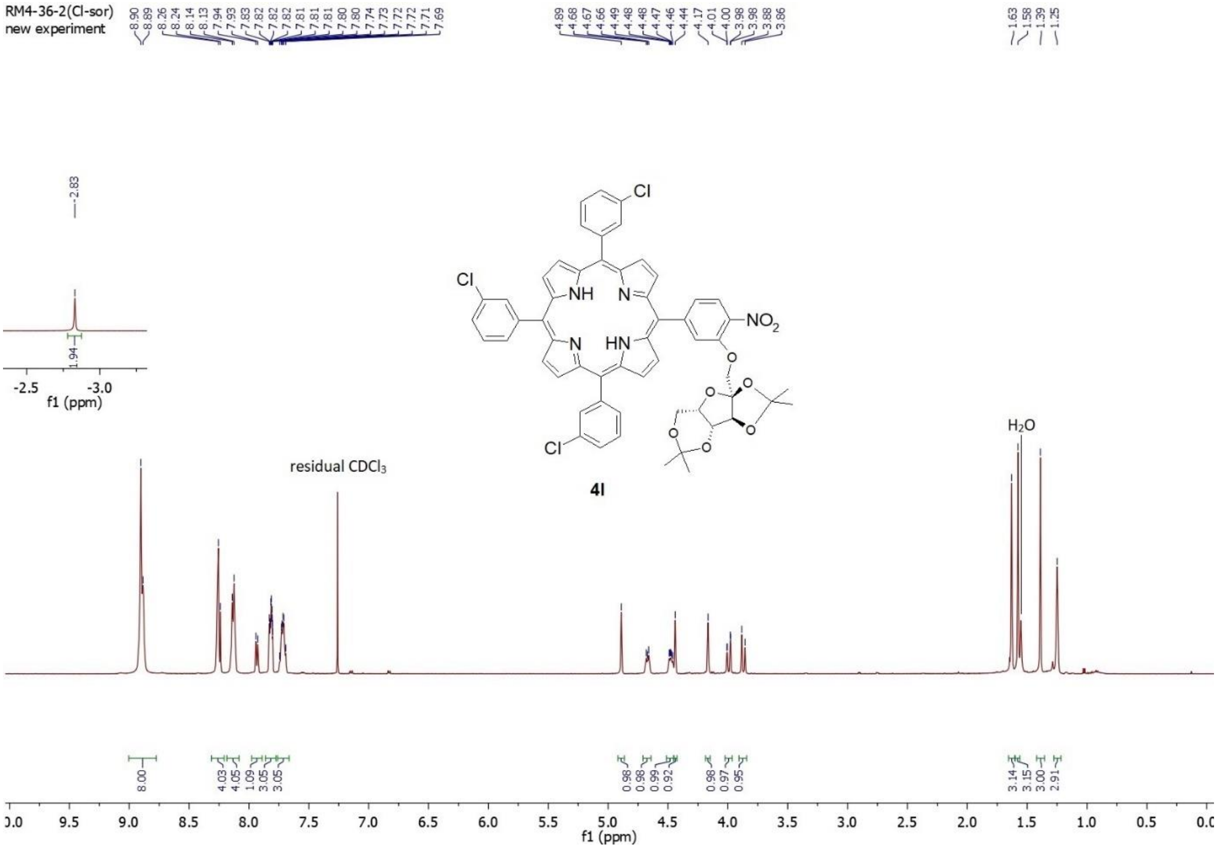

^{13}C NMR of **4l**

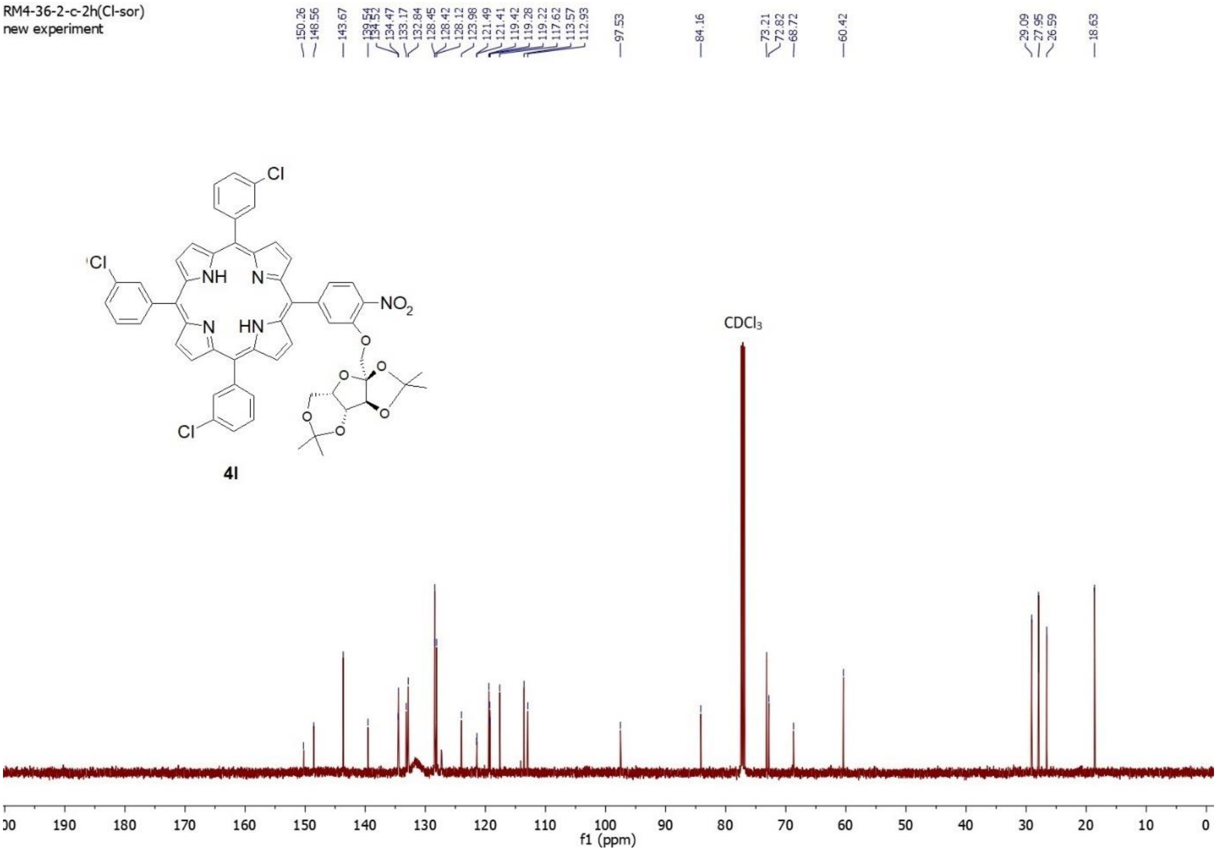

UV-VIS spectrum of **4l**

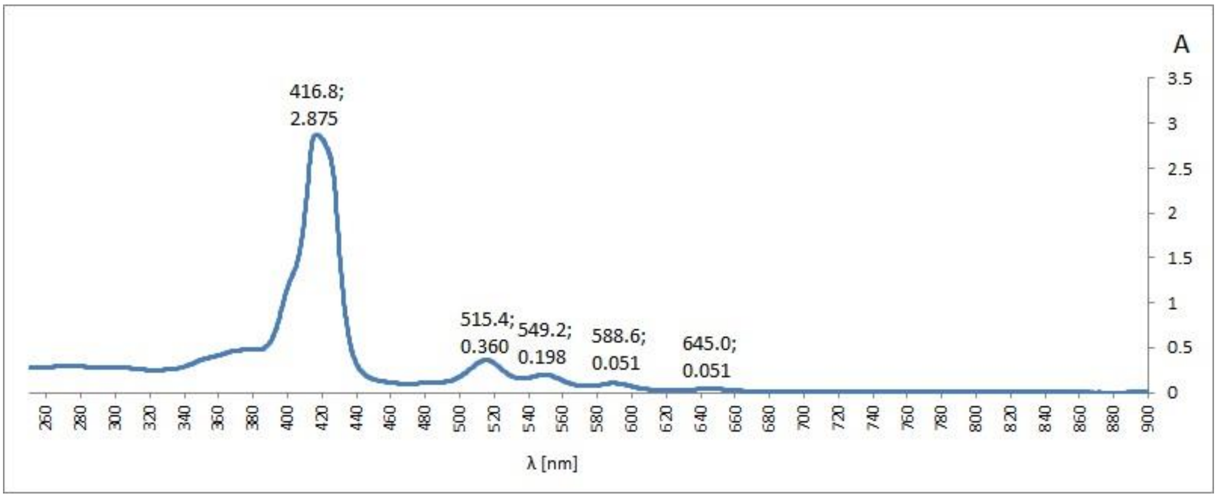

^{1}H NMR of (*rac*)-**4m**

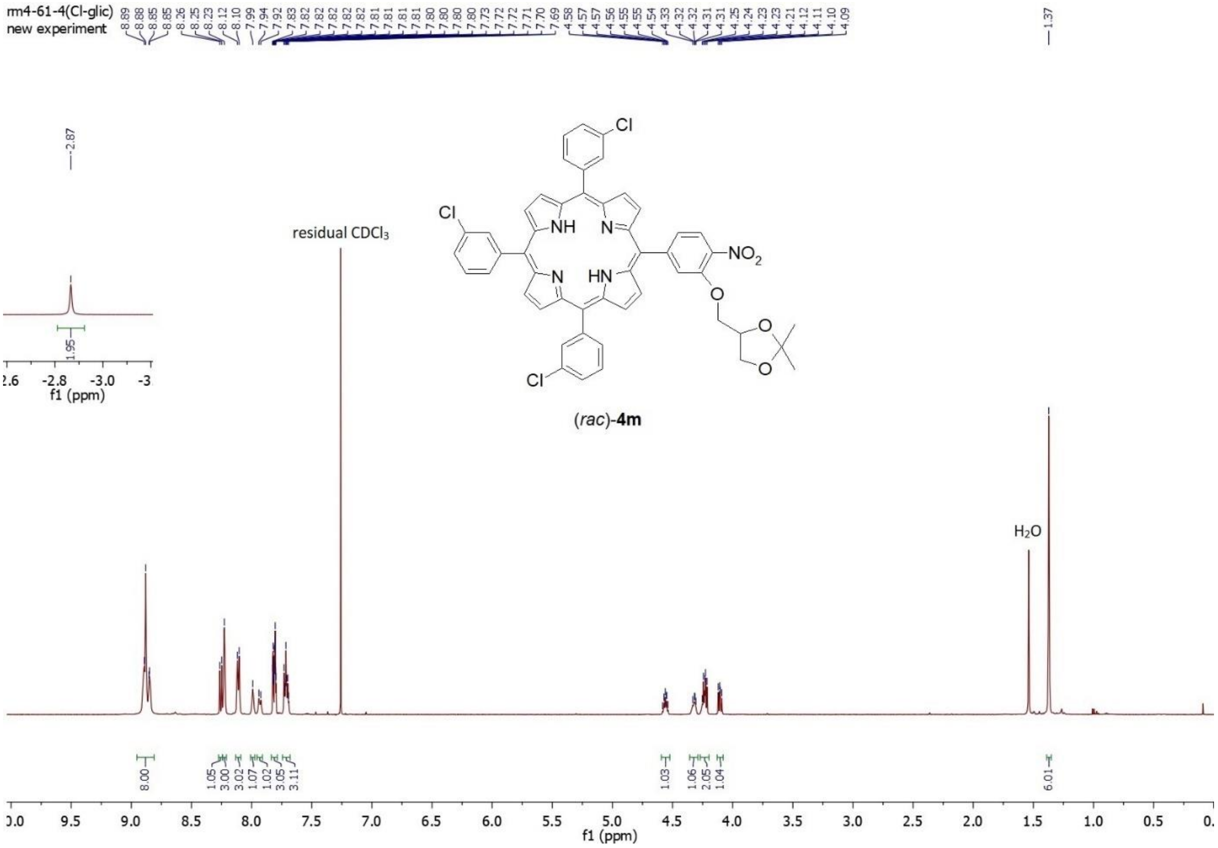

^{13}C NMR of (*rac*)-**4m**

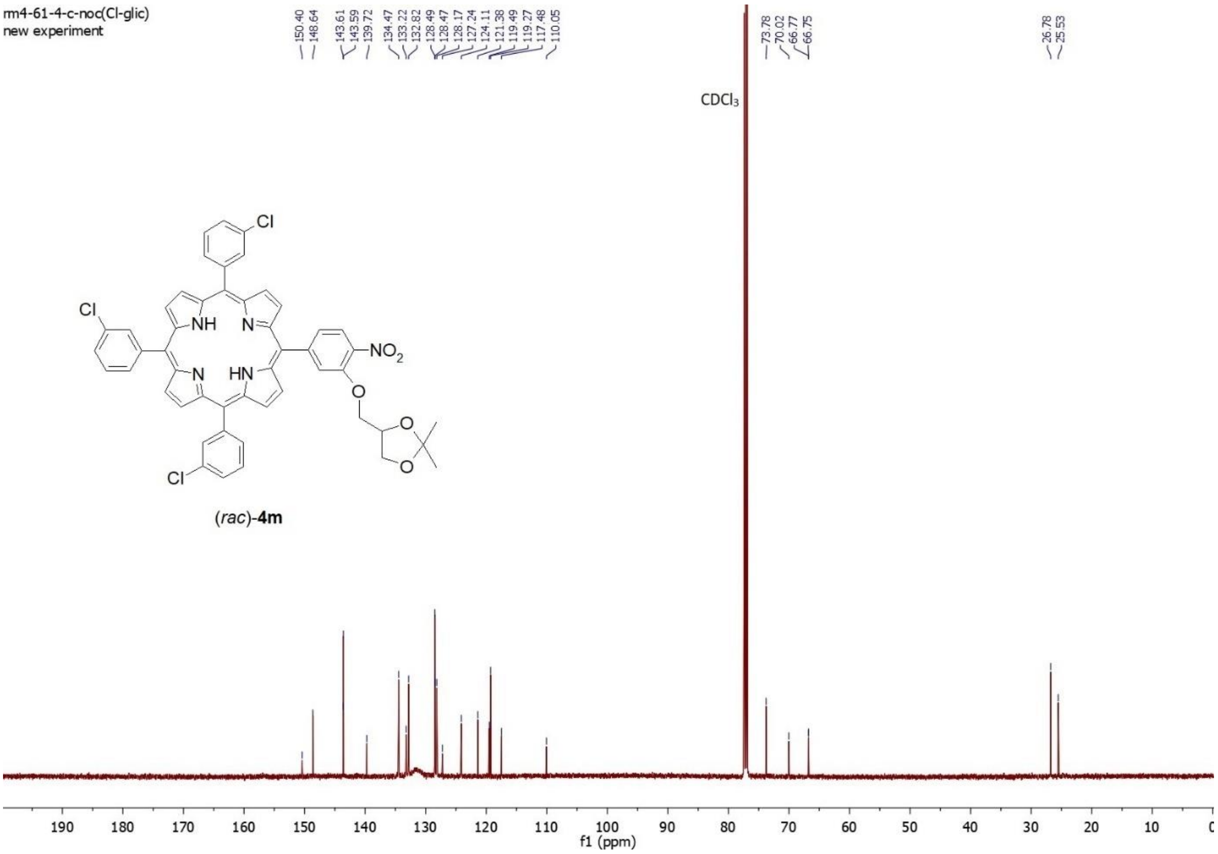

UV-VIS spectrum of (*rac*)-**4m**

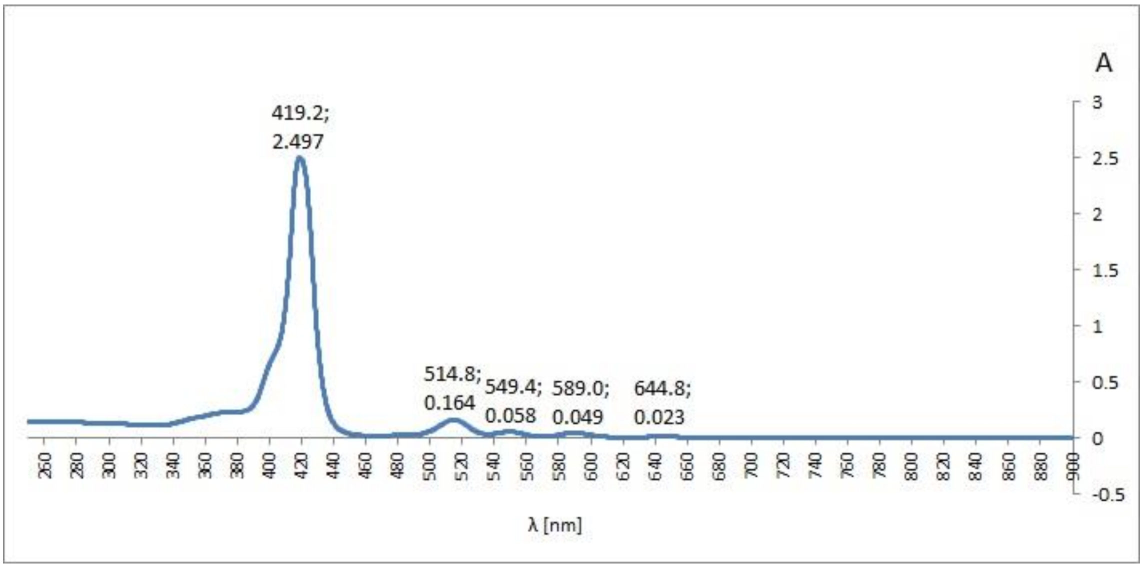

^{1}H NMR of **4o**

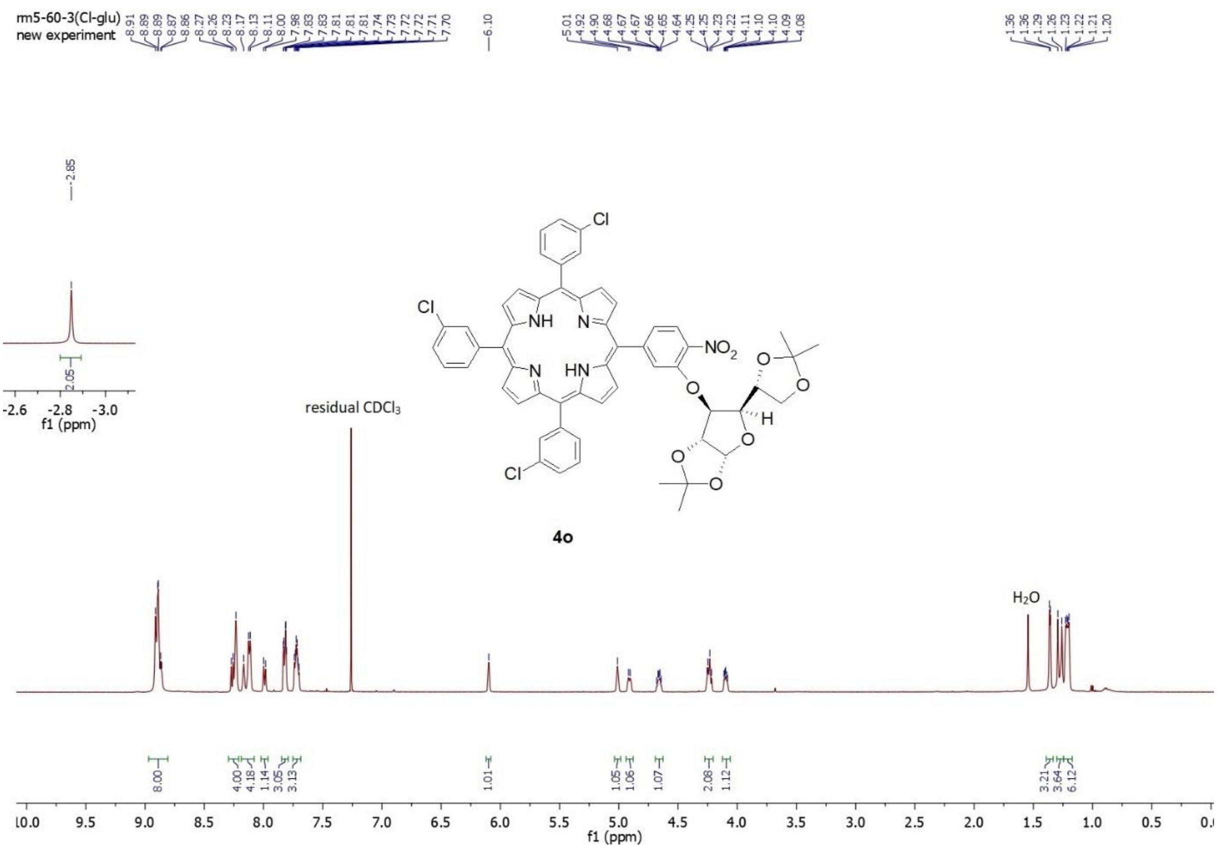

^{13}C NMR of **4o**

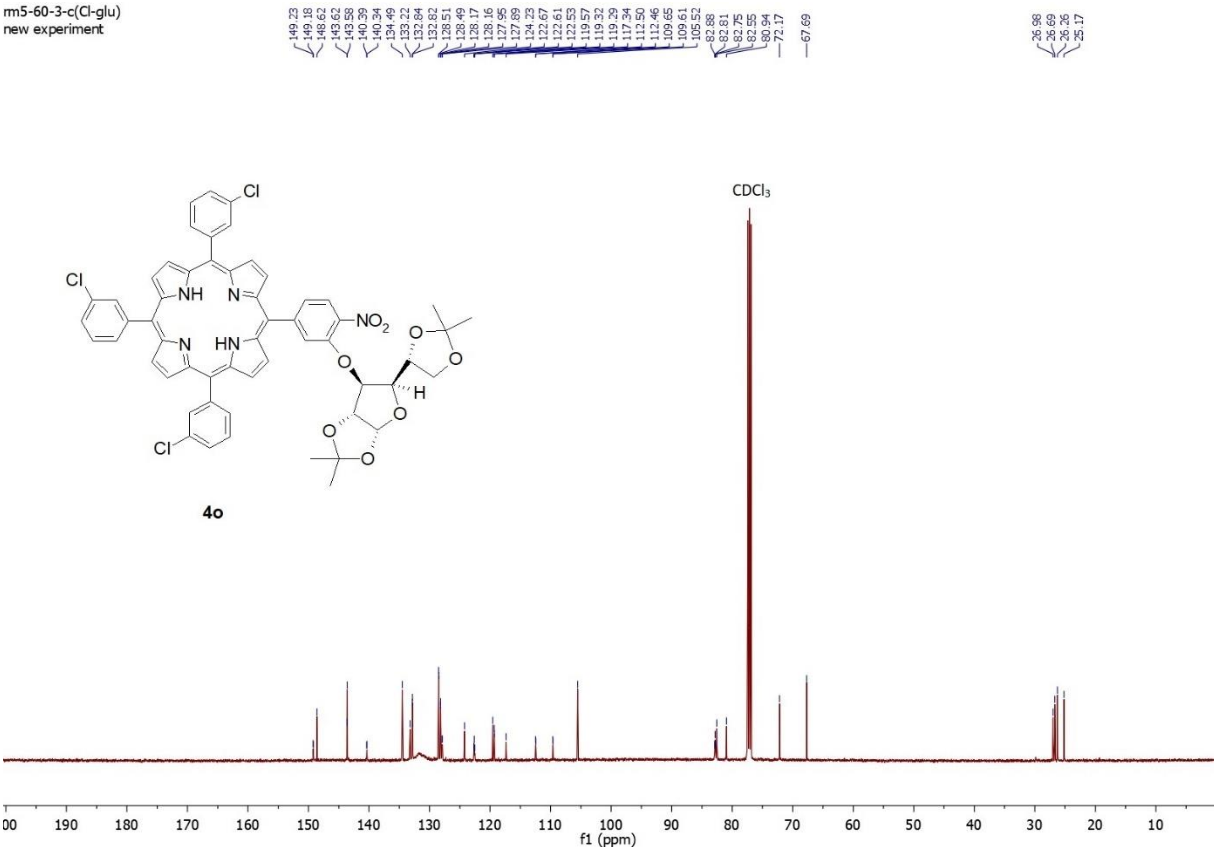

UV-VIS of **4o**

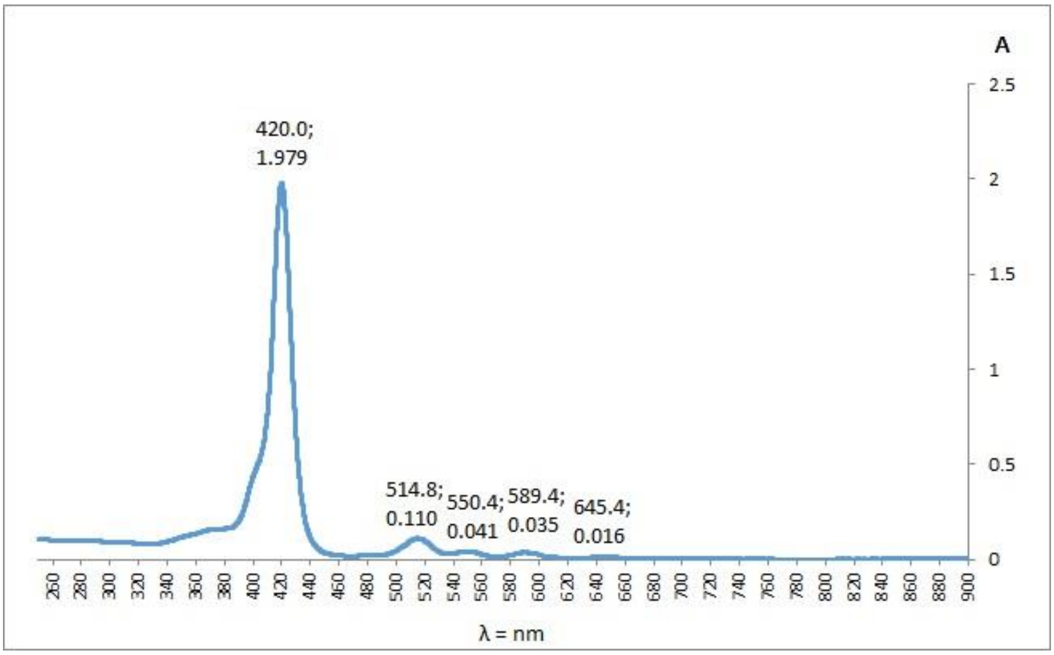

A
2.5
2
1.5
1
0.5
0
420.0;
1.979
514.8;
0.110
550.4;
0.041
589.4;
0.035
645.4;
0.016
260
280
300
320
340
360
380
400
420
440
460
480
500
520
540
560
580
600
620
640
660
680
700
720
740
760
780
800
820
840
860
880
900
λ = nm

^{1}H NMR of **5a**

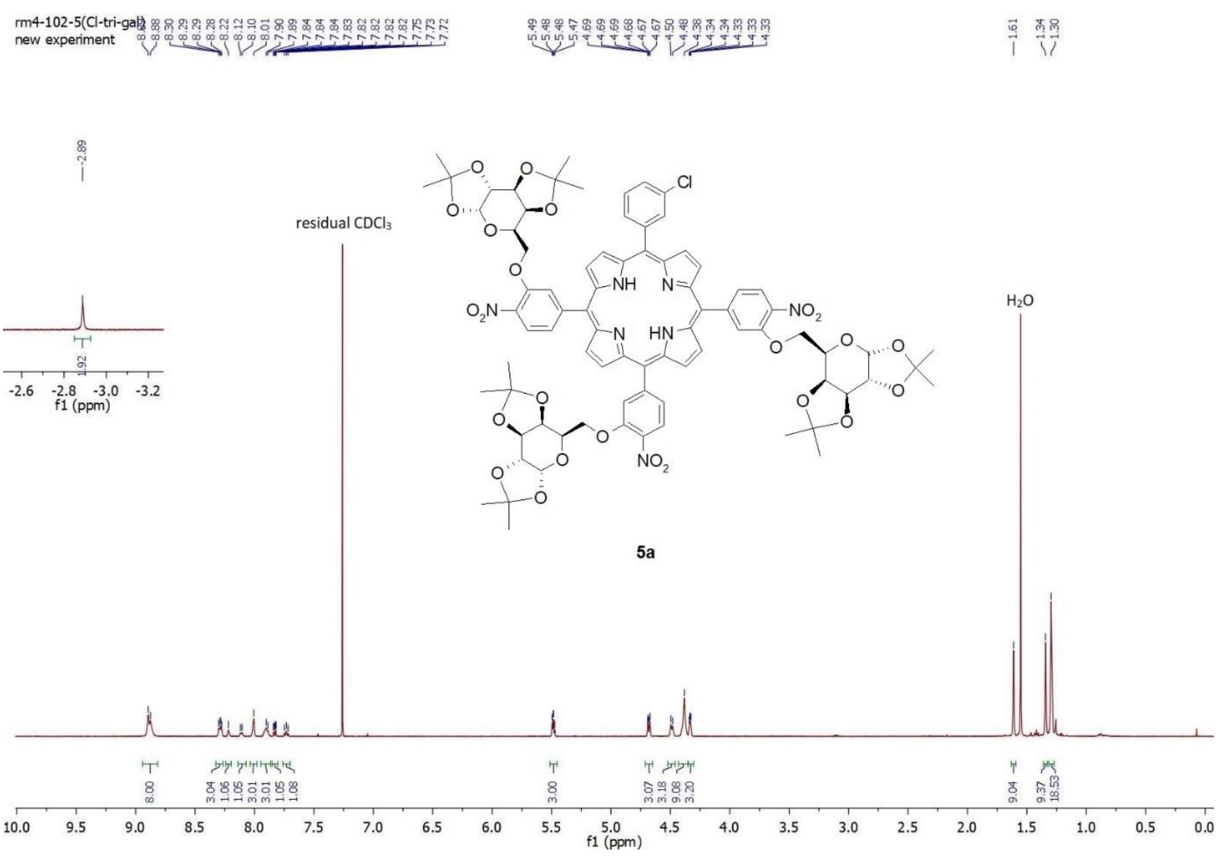

^{13}C NMR of **5a**

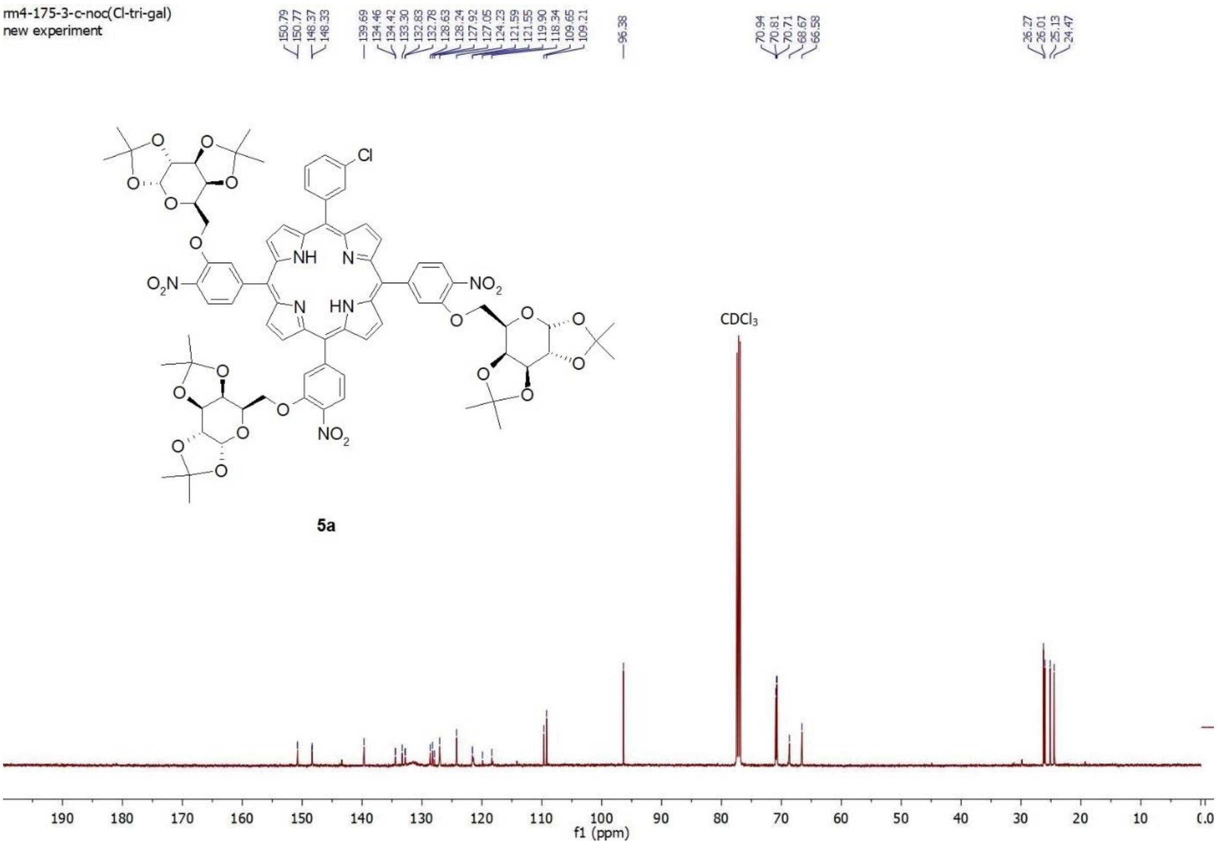

UV-VIS spectrum of **5a**

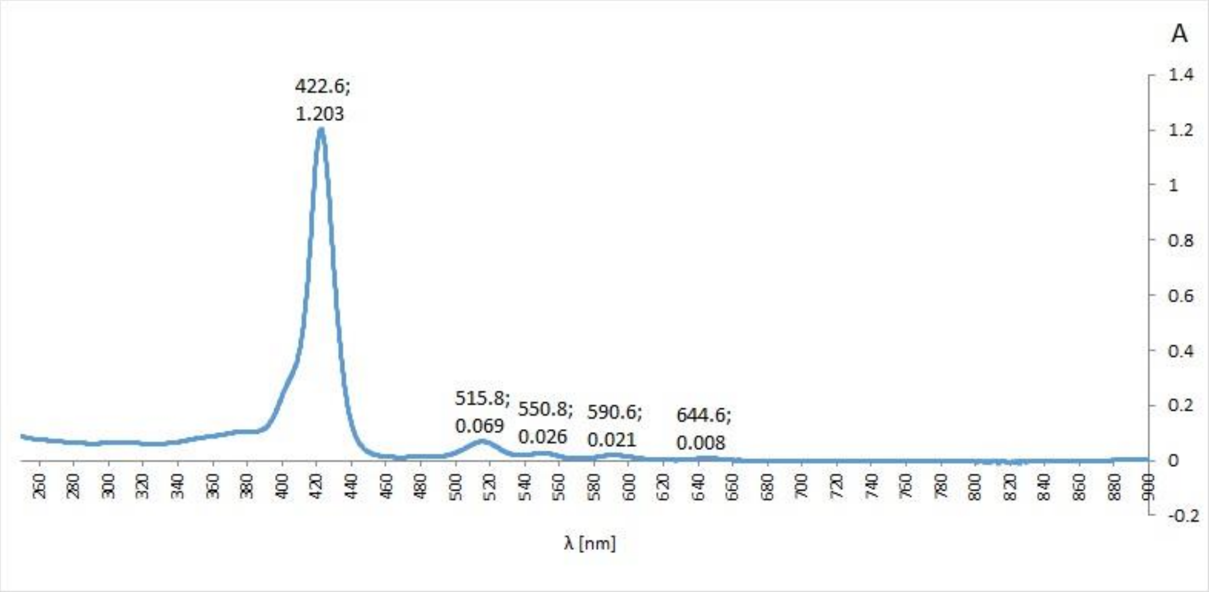

^{1}H NMR spectrum of **5b**

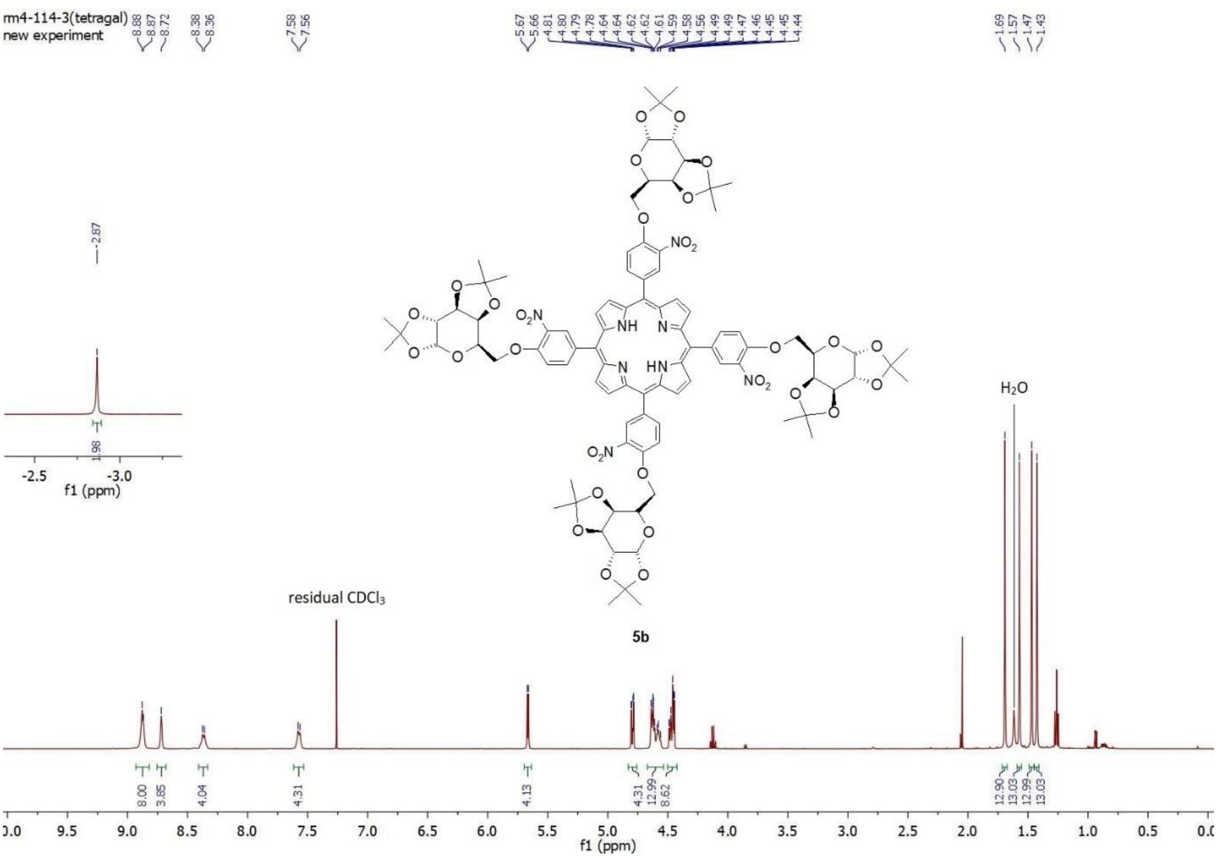

^{13}C NMR spectrum of **5b**

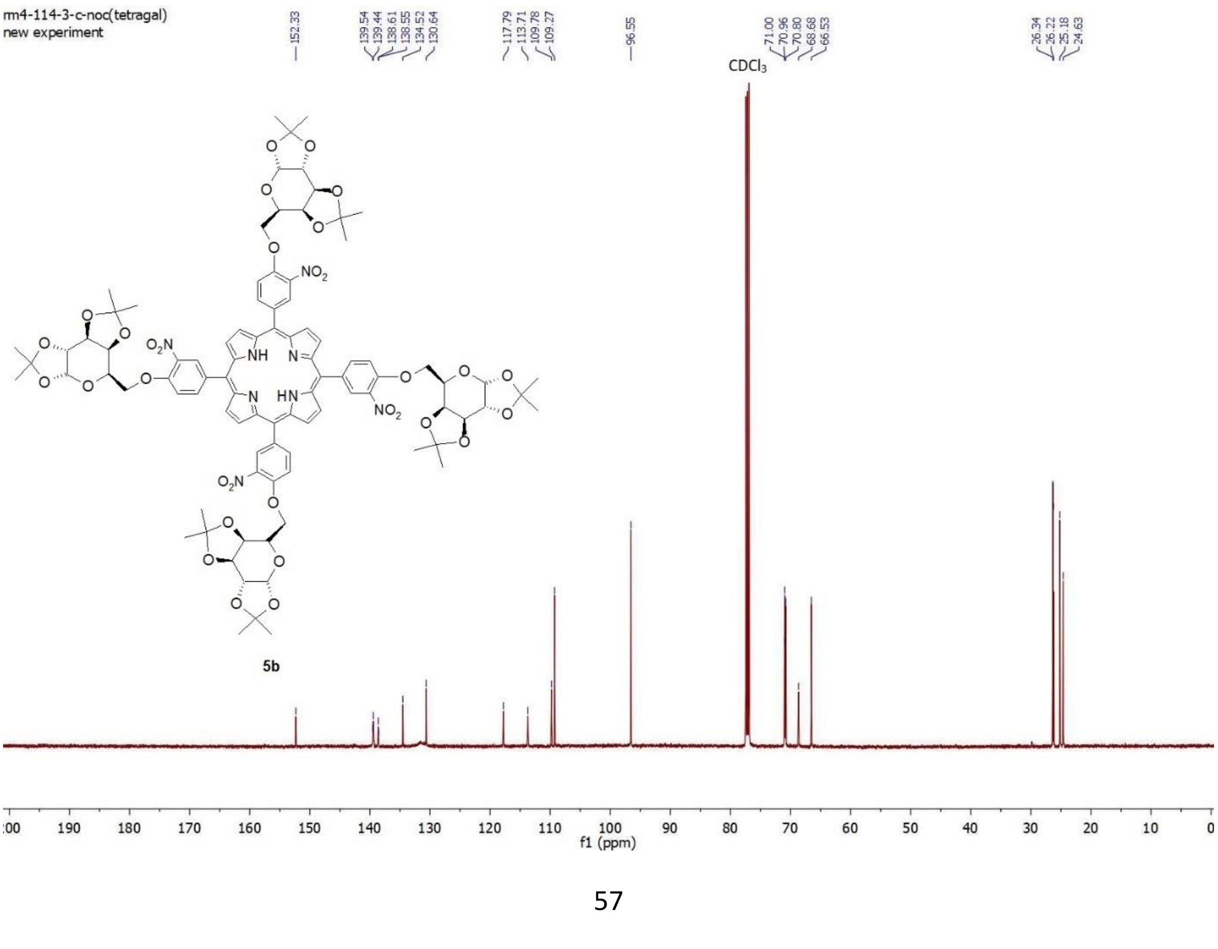

UV-VIS spectrum of **5b**

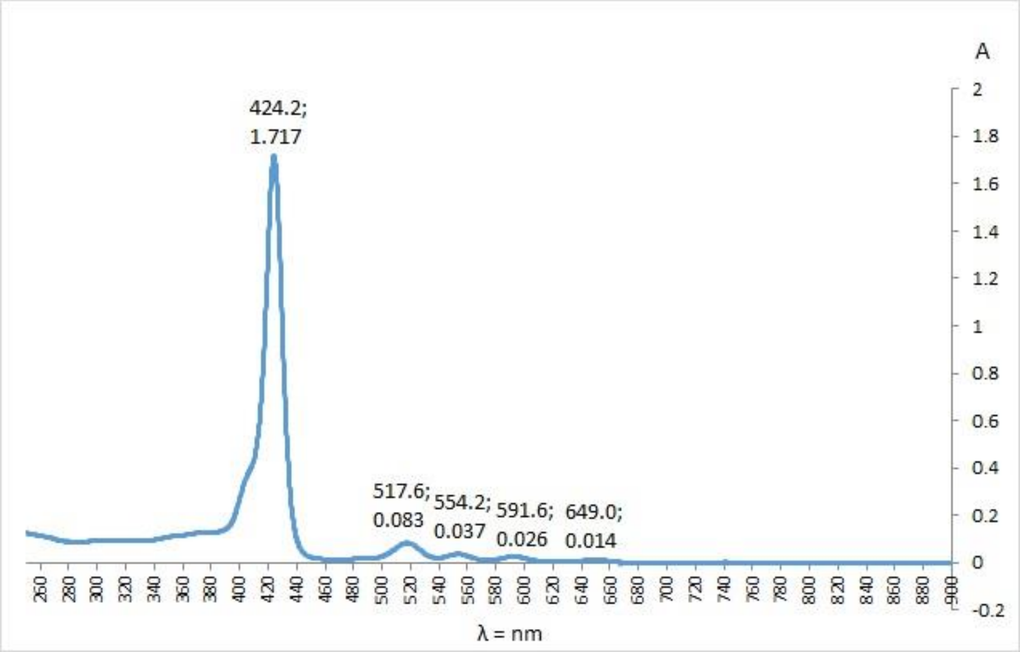

^{1}H NMR of **6a**

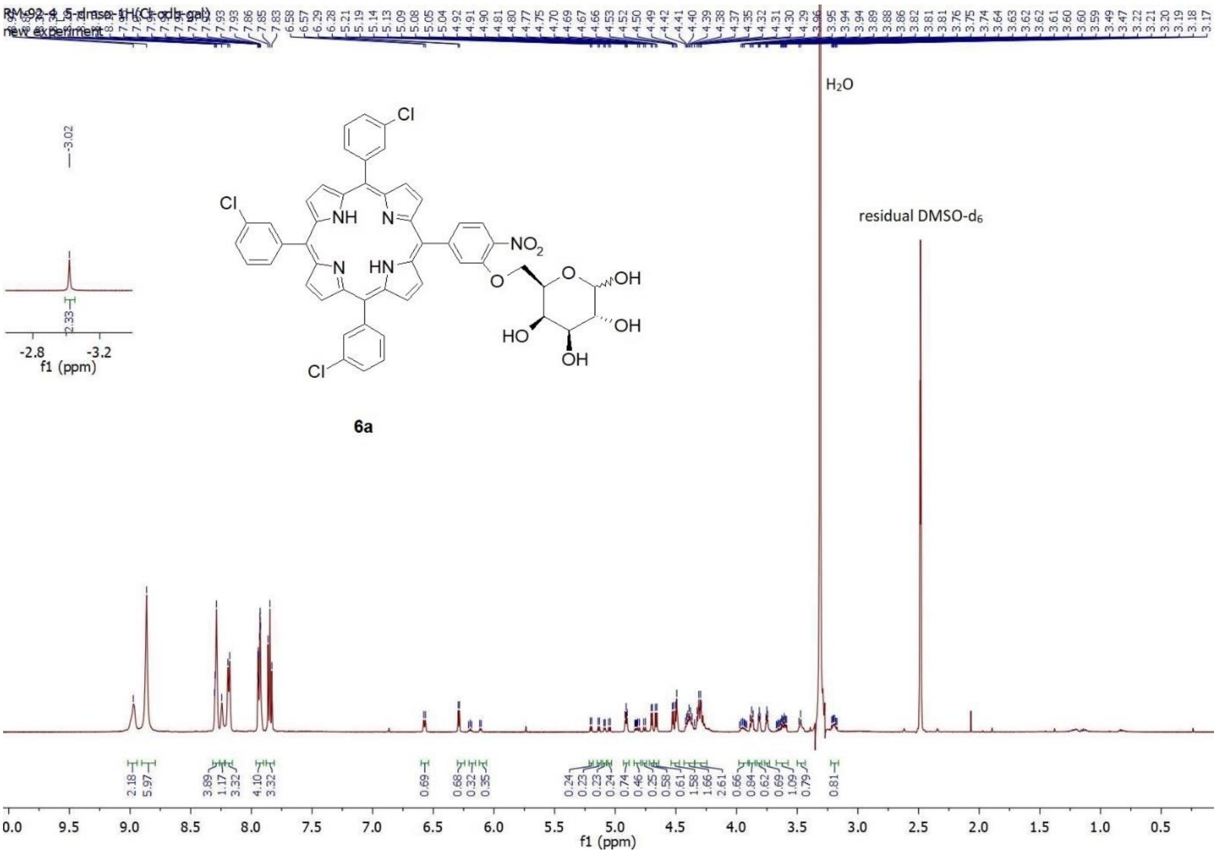

^{13}C NMR of **6a**

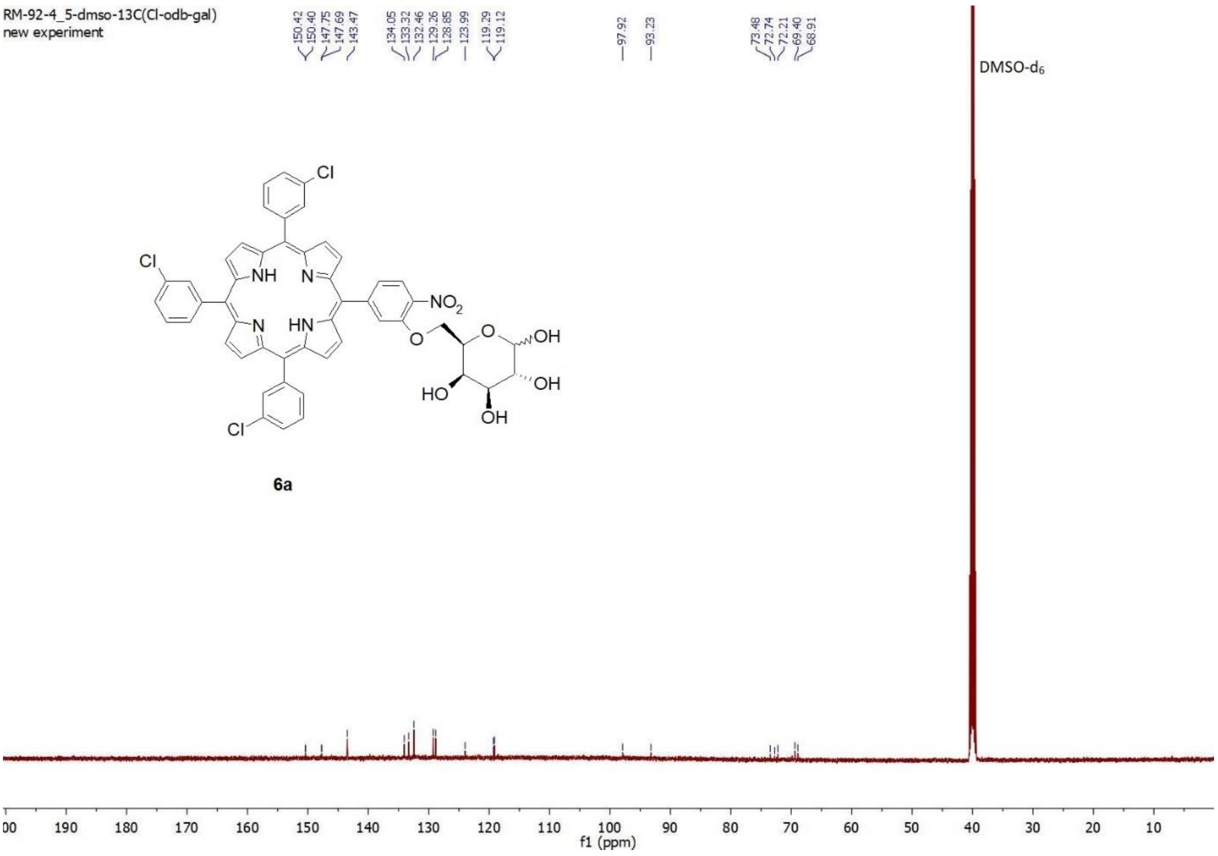

UV-VIS of **6a**

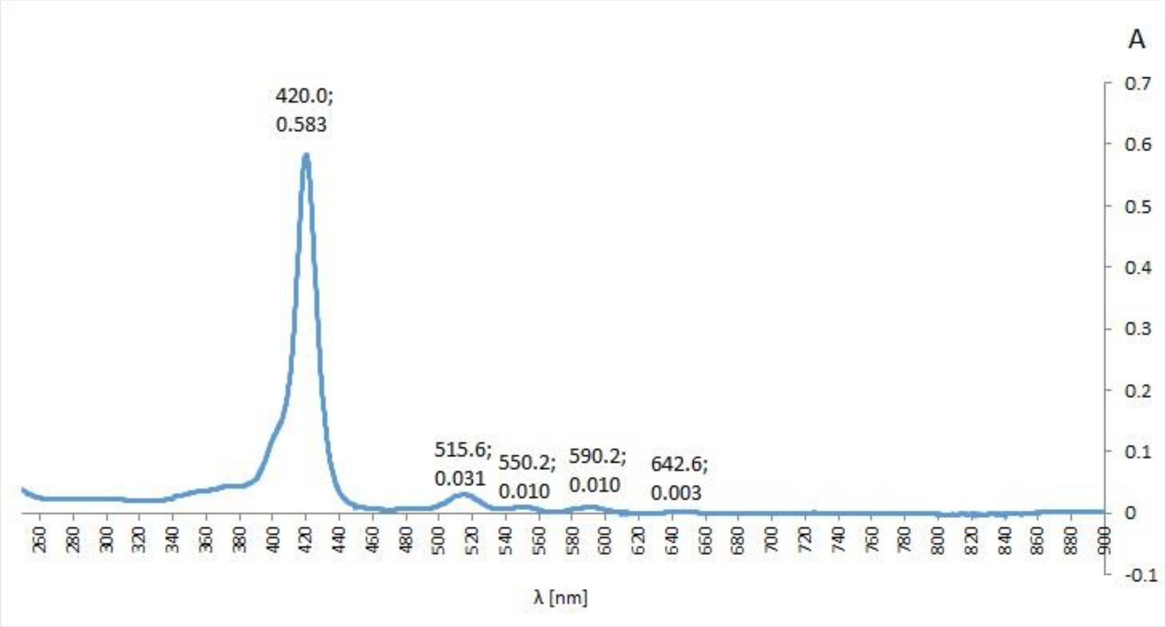

A
0.7
0.6
0.5
0.4
0.3
0.2
0.1
0
-0.1
420.0;
0.583
515.6;
0.031
550.2;
0.010
590.2;
0.010
642.6;
0.003
260
280
300
320
340
360
380
400
420
440
460
480
500
520
540
560
580
600
620
640
660
680
700
720
740
760
780
800
820
840
860
880
900
λ [nm]

^{1}H NMR of (*rac*)-**6m**

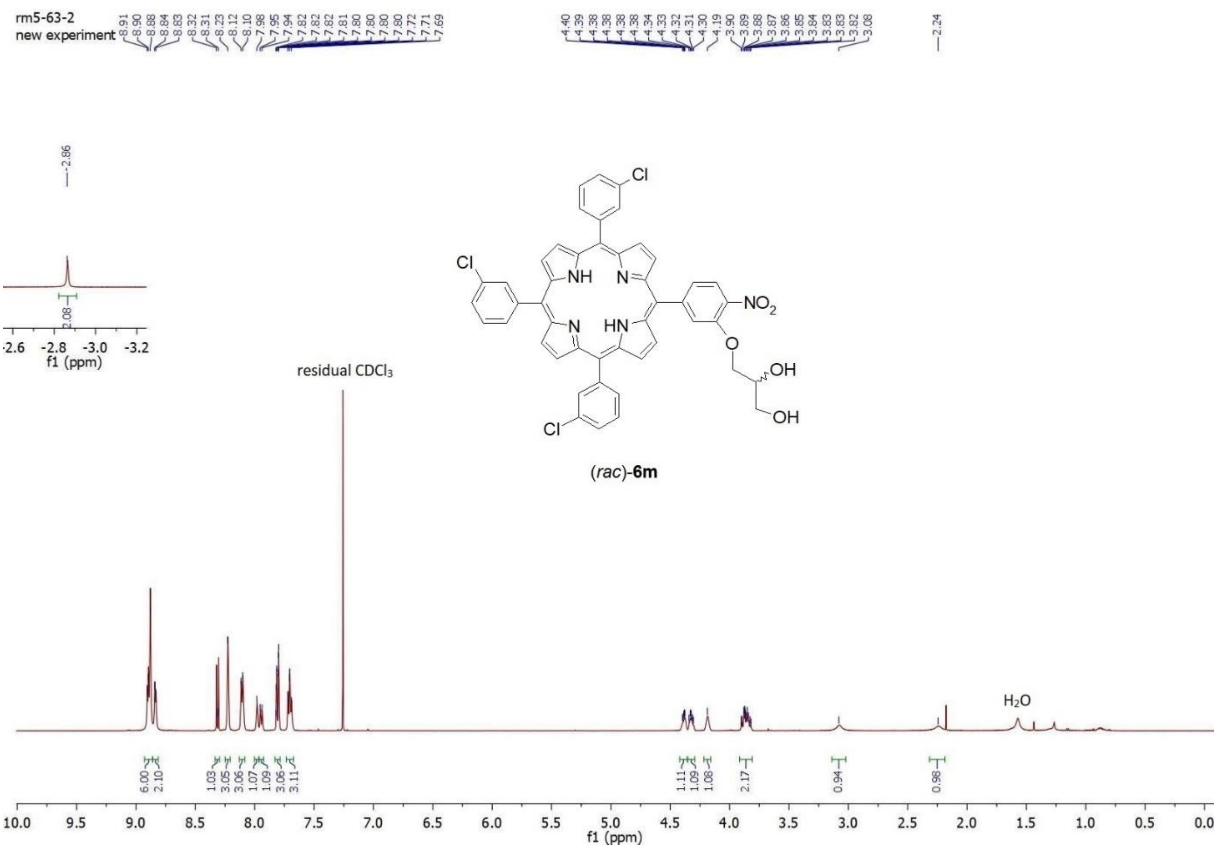

^{13}C NMR of (*rac*)-**6m**

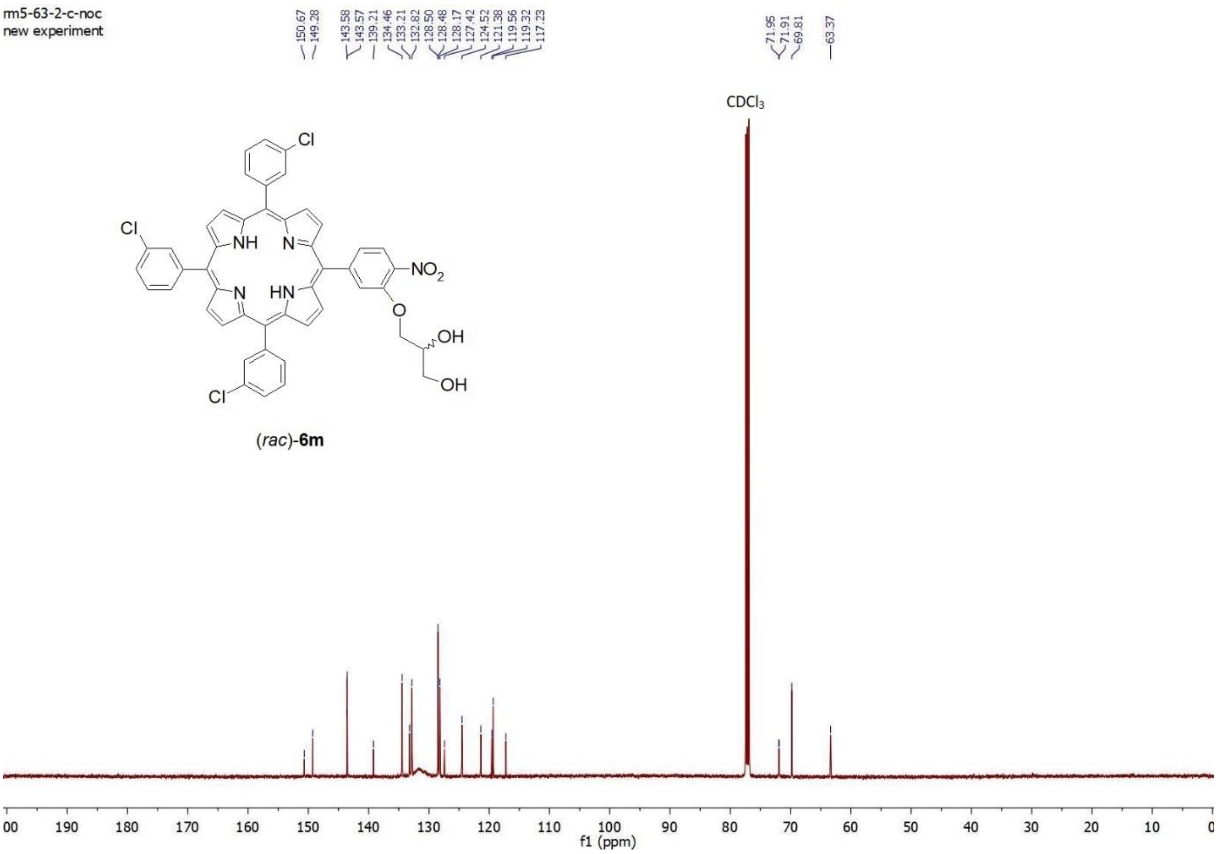

UV-VIS of (*rac*)-**6m**

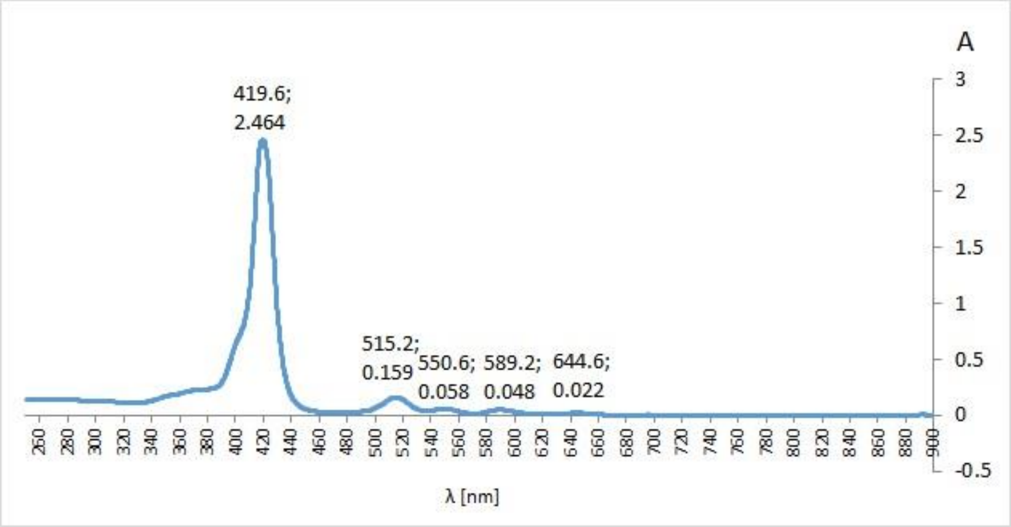

^{1}H NMR of **6o**

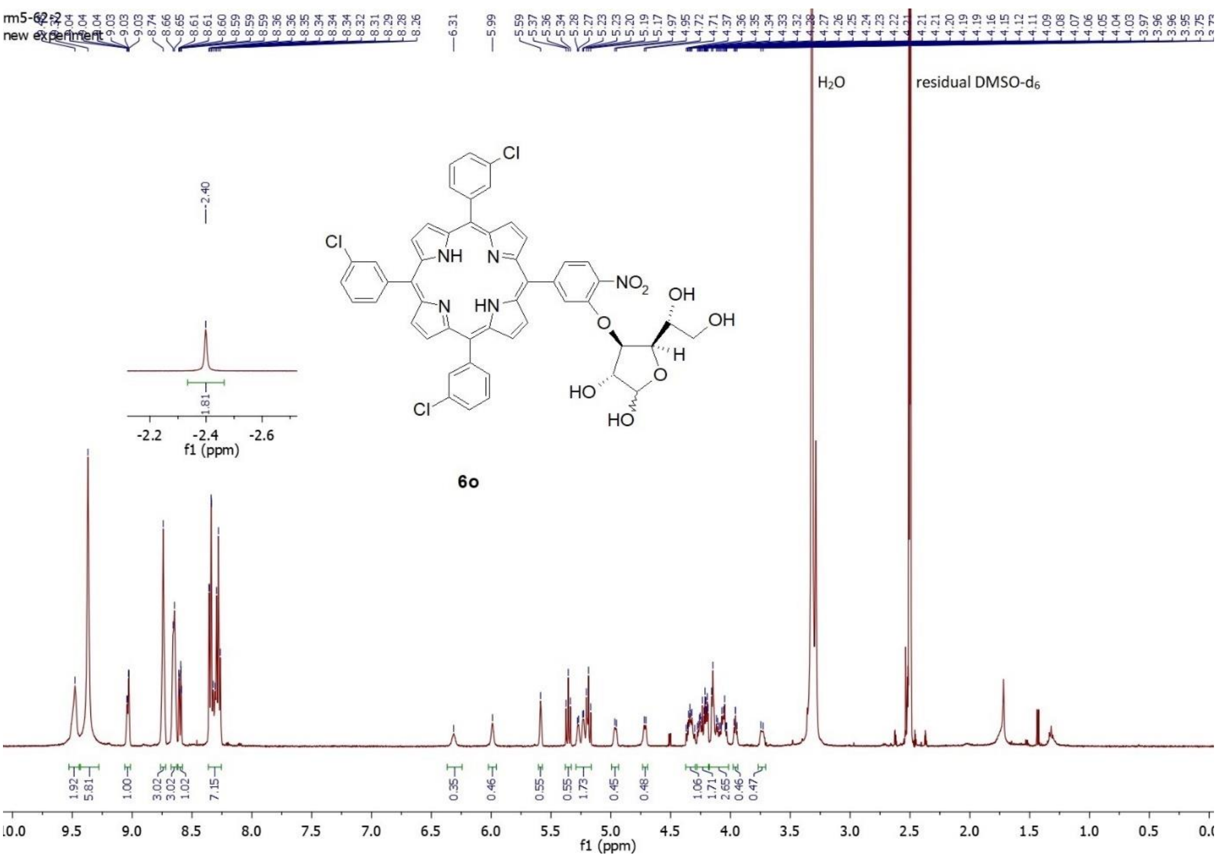

UV-VIS of **6o**

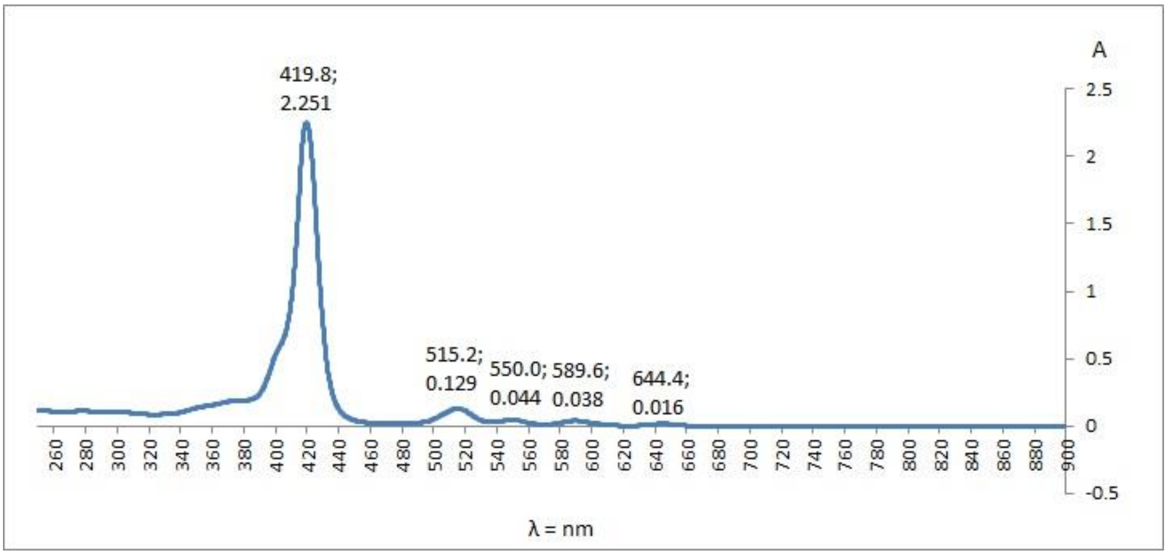

Supplement: Supplementary file 1 [file ijms-23-11321-s001.zip › ijms-1903663-supplementary.pdf]
